# Systematic Literature Reviews of Health-State Utility Values, Costs, and Healthcare Resource Use in Duchenne Muscular Dystrophy

**DOI:** 10.36469/001c.158537

**Published:** 2026-04-28

**Authors:** Chui-ying Yip, Eric N. Kemadjou, Daisy Stewart, Hannah Shapiro, Tom Macmillan

**Affiliations:** 1 F. Hoffmann-La Roche Ltd, Basel, Switzerland; 2 Source Health Economics, London, UK

**Keywords:** Duchenne muscular dystrophy, systematic review, utilities, economic, cost, burden

## Abstract

**Background:**

Duchenne muscular dystrophy (DMD) is a rare, X-linked inherited disorder starting in early childhood, characterized by progressive muscle loss and weakness, causing disability, loss of ambulation, and cardiopulmonary failure. Economic modeling is required to assess cost-effectiveness of new DMD therapies, but there are challenges from limitations and variations in currently available data.

**Objective:**

To assess the burden of DMD on patients, caregivers and healthcare systems, identifying inputs for cost-effectiveness models to inform economic assessments.

**Methods:**

Two systematic literature reviews (SLRs) were conducted. One SLR was performed to identify health state utility values (HSUV, original date 2019 with a 2023 update) and another to identify and cost data (original date 2023). Both SLRs were updated in 2024. Databases searched included Embase, MEDLINE, Centre for Reviews and Dissemination and EconLit, with additional hand-searching. PRISMA guidelines were followed. For each SLR, title/abstract and full-text screening were performed by two independent reviewers before data extraction. Validated quality assessment tools were used.

**Results:**

Across both SLRs, the burden increased as DMD progressed from early to late stages, indicated by decreasing HSUVs and increasing healthcare resource use and costs. The results highlighted large increases in burdens between early nonambulatory and late nonambulatory DMD in studies using the typical 4-state progression model. Recent publications using an 8-stage natural history model reported gradual increases in burden. There was heterogeneity between studies and a lack of long-term data in both SLRs.

**Discussion:**

The results highlighted the complex nature of progressive DMD, with heterogeneity and lack of long-term data across the studies. The findings suggest that HUI-3 may be the preferred tool for HSUV measurement in DMD, and the 8-stage natural history model may be preferable to typical 4-state models of DMD progression, to account for the observed heterogeneity and non-linear progression of this rare disease.

**Conclusions:**

These data indicate that the burden on patients, caregivers, and healthcare systems increases as DMD progresses. A wide range of inputs for economic modeling were identified, including insights into the way that the stages of DMD should be modeled to accurately reflect progression.

## INTRODUCTION

Duchenne muscular dystrophy (DMD) is an X-linked inherited disorder that starts in early childhood, caused by deficiency and/or loss of the functional dystrophin protein.^1^ A 2020 systematic review reported a global prevalence of DMD of 2.8 cases per 100 000 in the overall population and 7.1 cases per 100 000 males.^2^ DMD is characterized by progressive muscle loss and weakness, causing disability, loss of ambulation, and cardiopulmonary failure.^3^ Without respiratory support, median life expectancy ranges between 14.4 and 27.0 years.^4^ In the 1990s, ventilation support was introduced, increasing the median life expectancy of people with DMD, which now ranges between 21.0 to 39.6 years.^4^

DMD progression is typically defined in 4 stages: early ambulatory, late ambulatory, early nonambulatory, and late nonambulatory.^5^ People with DMD tend to reach the nonambulatory stage between 8 and 10 years of age, requiring a wheelchair or other mobility aids.^6^ After loss of ambulation, DMD progresses more rapidly, leading to upper extremity weakness, wasting of skeletal and cardiac muscle, and reduced respiratory function in the nonambulatory stages, which do not always occur in the same order.^3^ In light of the rapidity and variability of progression in the nonambulatory stages of DMD, a recent publication by Broomfield et al^7^ suggested a more granular 8-stage natural history model (NHM) of DMD progression. The NHM is structured around 8 health states that may not necessarily occur in order and that are grouped into 5 overarching stages (early and late ambulatory, transfer, and early and late nonambulatory).^7^

In DMD, the most significant clinical etiologies include muscle weakness, respiratory dysfunction, cardiomyopathy, bone fragility, and cognitive impairment.^8,9^ In addition, loss of ambulation reduces autonomy and the ability to perform day-to-day activities, which can lead to people with DMD feeling helpless, sad, frustrated, angry, and fearful.^10^ DMD also impacts the well-being of informal caregivers, such as parents and guardians, leading to impaired health-related quality of life (HRQoL), poor sleep quality, reduced family function, depression, pain, stress, sexual dysfunction, and negative impact on work life and productivity.^11^ However, HRQoL in both people with DMD and their caregivers is complex; both may report markedly better HRQoL scores than would be expected by the general population.^12–14^

DMD also presents a considerable economic burden for caregivers and the healthcare system. Caregivers experience financial toxicity from out-of-pocket costs and loss of income due to substantial care-related activities.^11^ The individual and societal costs of DMD increase steeply with disease progression, with lifetime costs estimated at €4.98 million per person with DMD in Germany (2025 euros)^15^ People with DMD require specialist care from multidisciplinary teams, who must be trained to manage DMD progression and its associated symptoms.^16^ The reported annual mean per-patient direct medical cost of DMD is highly variable and ranged between $7300 and $28 590 in a study of German, Italian, UK, and US patients (2012 international dollars).^17^ The direct medical costs may increase by a factor of 16 between the earliest and latest stages of DMD.^18^

The current standard of care for DMD is corticosteroid treatment, which can slow the progression of the disease,^6^ but this does not address the underlying genetic cause of DMD, so progression will still occur.^19^ In addition, corticosteroid therapy is linked to adverse events including bone fractures, excessive weight gain and hair growth, mood disorders, and cushingoid appearance.^20^

Recent treatments that aim to address the underlying causes of DMD include antisense oligonucleotides^21^ and adeno-associated virus gene therapies,^22^ which can modify the course of DMD progression. These have the potential to greatly increase patient HRQoL, thereby lowering the considerable burden on both patients and caregivers.^23^ Additionally, compared with current standard-of-care corticosteroids, these treatments may help to lessen the burden of DMD on healthcare resource utilization (HCRU) and costs to the healthcare system, owing to a reduction in long-term corticosteroid administration and specialist care. Health economic modeling is required to demonstrate whether these new therapies can be cost-effective, but there are challenges due to limitations and variations in the currently available data.

This article reports two systematic literature reviews (SLRs), the first being an update to the health state utility review published by Szabo et al^24^ and the second being a de novo HCRU and costs review. In combination, these SLRs aim to obtain a full picture of the burden of progressive DMD on patients, caregivers, and the healthcare system. In addition, these findings allow the gathering of inputs necessary to create a robust, reliable, and consistent economic model for the assessment of treatments for DMD.

## METHODS

DMD stages were reported using a number of different models, including 4-stage models with early/late ambulatory and nonambulatory stages, Brooke scoring,^25^ and the NHM.^7^ These models have been mapped together in **Supplementary Table S1**, to show where they conceptually align.

Both SLRs followed a pre-approved protocol that was registered with PROSPERO (CRD42023483771) and are reported in accordance with the Preferred Reporting Items for Systematic Reviews and Meta-Analysis (PRISMA) guidelines.

### Search Strings and Inclusion/Exclusion Criteria

The original SLR for health state utility values (HSUVs) was performed and published by Szabo et al.^24^ The methodology was modified for two updates conducted in 2023 and 2024.

Embase and MEDLINE databases (dates shown in **Supplementary Table S2**) were searched using the search strings shown in the **Supplementary Material**, and results were assessed against the inclusion/exclusion criteria shown in **Table 1.**

**Table 1.** Inclusion/Exclusion Criteria

**Characteristics**	**HSUV SLR**		**HCRU and Cost SLR**	
**Inclusion Criteria**	**Exclusion Criteria**	**Inclusion Criteria**	**Exclusion Criteria**	
Population	Patients of any age with DMD, with any pathogenic mutation, deletion, or duplication Caregivers/parents of patients with DMD Clinicians who manage patients with DMD “Layperson respondents:” Individuals who represent general population^a^ Mixed populations: If ≥80% of patients met inclusion/exclusion criteria or outcomes are reported separately for these patients	Mixed populations: If <80% of patients met inclusion/exclusion criteria or outcomes are not reported separately for these patients	Patients of any age with DMD, with any pathogenic mutation, deletion, or duplication Mixed populations: If ≥80% of patients met inclusion/exclusion criteria or outcomes are reported separately for these patients	Mixed populations: <80% of patients meet the inclusion/exclusion criteria or outcomes are not reported separately for these patients Studies of diagnosis/screening of patients pre-DMD diagnosis
Intervention / comparators	No restriction	N/A	No restriction	–
Outcomes	Utilities/disutilities/QALYs for health states or adverse events, including: Directly elicited utilities/preference values (eg, SG or TTO) Indirectly elicited utilities/preference values (eg, using the EQ-5D, HUI-3, SF-6D, DMD-QoL-8D) Mapping algorithms allowing data from disease-specific/generic HRQoL measures to be mapped to preference-based HSUVs, or data to be mapped from one population to another	Any general or disease-specific HRQoL measures with no associated utility values for health states	Measures of costs, including: Direct costs (medical and nonmedical) Indirect costs (including societal costs) Measures of healthcare resource use Cost drivers	Outcomes not listed
Date limits	2023 SLR update: 11 Jan 2019 – present 2024 SLR update: 12 Oct 2023 – present		2023 de novo SLR: 2014–present 2024 SLR update: 12 Oct 2023–present	

Abbreviations: DMD, Duchenne muscular dystrophy; DMD-QoL-8D, Duchenne Muscular Dystrophy Quality of Life 8-Dimensions; HCRU, healthcare resource use; HRQoL, health-related quality of life; HSUV, health state utility value; HUI-3, Health Utilities Index Mark–3; N/A, not applicable; NMA, network meta-analysis; QALYs, quality-adjusted life-years; SF-6D, Short-Form 6-Dimensions; SG, standard gamble; SLR, systematic literature review, TTO time trade-off.

Study types included were any reporting original HSUV or HCRU/cost data as applicable per SLR. Reviews/editorials/commentaries/letters, case reports, and in vitro/animal studies were excluded. Relevant SLRs/NMAs were included at title/abstract screening stage, so their bibliographic reference lists could be hand-searched for relevant studies.

^a^Layperson respondents were considered in case, for example, vignette-based exercises using members of the general population were identified as sources of utility estimates.

For the HCRU and cost SLR, Embase, MEDLINE, University of York Centre for Reviews and Dissemination DARE, Health Technology Assessment (HTA) and NHS EED databases, and EconLit were interrogated (dates shown in **Supplementary Table S3**) with the search strings in the **Supplementary Material,** and results were assessed against the inclusion/exclusion criteria shown in **Table 1.**

### Hand-Searching

Bibliographies of any included publications or identified SLRs were hand-searched to ensure the capture of all relevant sources. Additional hand-searching was conducted in the following sources:

- **Conference proceeding**s: Proceedings of relevant conferences (shown in **Supplementary Table S4**) held between 2020 and 2024 were searched via the conferences’ online platforms or downloadable abstract books.
- **HTA bodies**: Previous submission documents from the HTA agencies shown in **Supplementary Table S5** were reviewed for relevant data.

### Study Screening

For each SLR, title/abstract screening was performed by two independent reviewers using the inclusion/exclusion criteria, shown in **Table 1**. Discrepancies were resolved through discussion, with input from a third, senior reviewer if needed. Full-text papers were screened with the same process and same inclusion/exclusion criteria.

If multiple reports from a single study were identified, the earliest full-text manuscript reporting the primary outcome was designated the primary publication. Other citations for the same study were considered “linked” citations. Only linked citations offering unique information were included in the review; others were excluded. For example, if a conference abstract and a following manuscript reported the same data, the later published manuscript was prioritized and extracted, and the conference abstract was excluded, with the reason of “superseded.” If the data points were different between two linked citations, both citations were included and extracted.

For cases where a very large number of relevant publications were identified prior to data extraction, publications were prioritized using additional inclusion criteria, shown in **Supplementary Table S6**. Publications not meeting these criteria were deprioritized and did not undergo data extraction.

### Data Extraction

Data from the included publications were extracted into standardized data extraction tools in Microsoft Excel. Data extraction was performed by one reviewer and quality checked by a second reviewer, with input from a third, senior reviewer if needed.

### Additional Search for Health Economic Evaluations

An additional systematic search was conducted to identify economic evaluations to provide context and to assess modeling methods used to evaluate treatments for DMD. Embase, MEDLINE, and Econlit were searched between the date of database inception and 10 January 2024. The methodology and results for this SLR can be found in the **Online Supplementary Material**.

### Study Quality Assessment

Quality assessment (QA) was performed on full-text publications, using tools relevant to the data types being reported. Heterogeneity of identified studies was not formally assessed. Abstracts and posters did not undergo QA, as they do not typically report enough information to perform a full QA. A QA checklist from Papaioannou et al^26^ was used to assess publications identified in the HSUV SLR. A checklist from Molinier et al^27^ was used for the HCRU and cost SLR, as this checklist is most suitable for observational/nonrandomized cost-related studies.

## RESULTS

### Health State Utility Value Results

Publications from the SLR by Szabo et al^24^ were updated in 2023 and again in 2024. The findings from each iteration are described below.

In the Szabo et al review (database inception: 11 January 2019), 888 publications were identified, of which 8 publications, reporting on 5 unique studies, were selected for final inclusion.^24^

In the 2023 update to the SLR (11 January 2019–11 October 2023), 537 publications were identified of which 13 publications, reporting on 13 unique studies, were selected for final inclusion.

In the 2024 SLR update (12 October 2023–9 January 2024), 42 publications were identified, of which 3 publications, reporting on 3 unique studies were selected for final inclusion.

**Figure 1.**
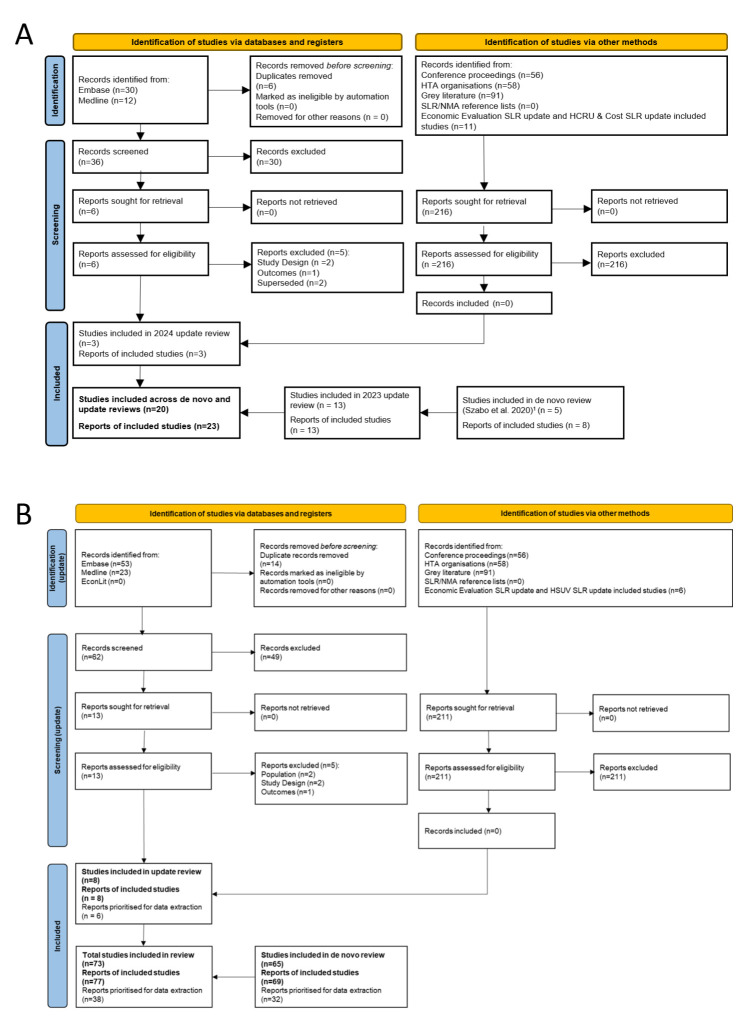
PRISMA Diagrams of Included Studies from All Iterations of the (**A**) HSUV SLRs and (**B**) HCRU SLRs Abbreviations: HCRU, healthcare resource use; HSUV, health state utility value; HTA, health technology assessment; NMA, network meta-analysis; PRISMA, Preferred Reporting Items for Systematic Reviews and Meta-Analyses; SLR, systematic literature review. ^⁠1⁠^Szabo et al. 2020.^24^

Across all 3 iterations of the SLR, 23 publications reporting on 20 unique studies were identified. PRISMA diagrams are shown in **Figure 1.**

HSUVs were reported for patients only in 17 publications,^12,28–41^ for both patients and caregivers in 5 publications,^17,42–45^ and for caregivers only in 1 publication.^46^

Six publications used patient-reported measures to derive utilities^29,33,34,38,42,44^; however, proxy individuals were often used to derive utilities for patients, as few utility instruments are validated for self-completion by children. The most commonly-used proxy respondents were caregivers,^12,17,29,31,34,37,38^ and caregivers helped patients complete the responses in several studies.^28,29,34,39,43^ Three publications reported both patient and caregiver-derived utilities.^34,38,47^ Two publications used clinician respondents to derive DMD utilities,^35,36^ three publications used layperson respondents,^32,40,45^ and one study used a combination of patients and layperson respondents to derive the utilities.^30^

Among the publications reporting utilities for patients, the majority used indirect methods to derive the utilities. Indirect methods included generic HRQoL measures (EuroQol 5-Dimension [EQ-5D] and derivatives [10 publications^29,31,34,38–42,44^], the Health State Utilities Index (HUI)-2 [4 publications^29,38^]; and the HUI-3 [9 publications^12,17,28,29,35,36,43^], as well as disease-specific measurements (DMD-QoL [3 publications^33,37,40^]). One study used direct time trade-off (TTO) methodology to derive utilities for patients,^32^ another study used general public TTO elicitation with health states derived from patient interviews,^45^ and one study inferred utilities based on heart failure utilities and HRQoL scores for DMD.^30^

Some studies reported HSUVs for the overall DMD population only,^28,38,41,44^ or a specific health state,^29,38,42^ but most studies reported HSUVs for multiple health states related to DMD progression. The number of health states reported and health state definitions varied between the studies. HSUVs according to 4 health states (most commonly the “typical” early ambulatory, late ambulatory, early nonambulatory, late nonambulatory states) were reported in 7 publications.^12,17,29,32,34,37,43^ Study definitions of the health states ranged from simple age-based definitions to complex definitions incorporating factors including ventilation support requirements and upper limb/hand-to-mouth function (ULF/HTMF), as well as ambulatory status; definitions used in individual studies are shown in **Supplementary Table S7**. Two publications reported HSUVs specifically according to ventilation support requirements,^12,43^ one publication according to New York Heart Association class,^30^ and one publication according to physical and mental health status.^12^ Additionally, some studies reported utilities across additional health states, taking into account multiple factors including ambulatory status, ULF/HTMF, ventilation requirements, and/or presence of cardiomyopathy.^29,31,33,36,39,45^ A transitionary health state between the late ambulatory and early nonambulatory health states was also included in one study as part of an 8-stage model.^39^

**Figure 2.**
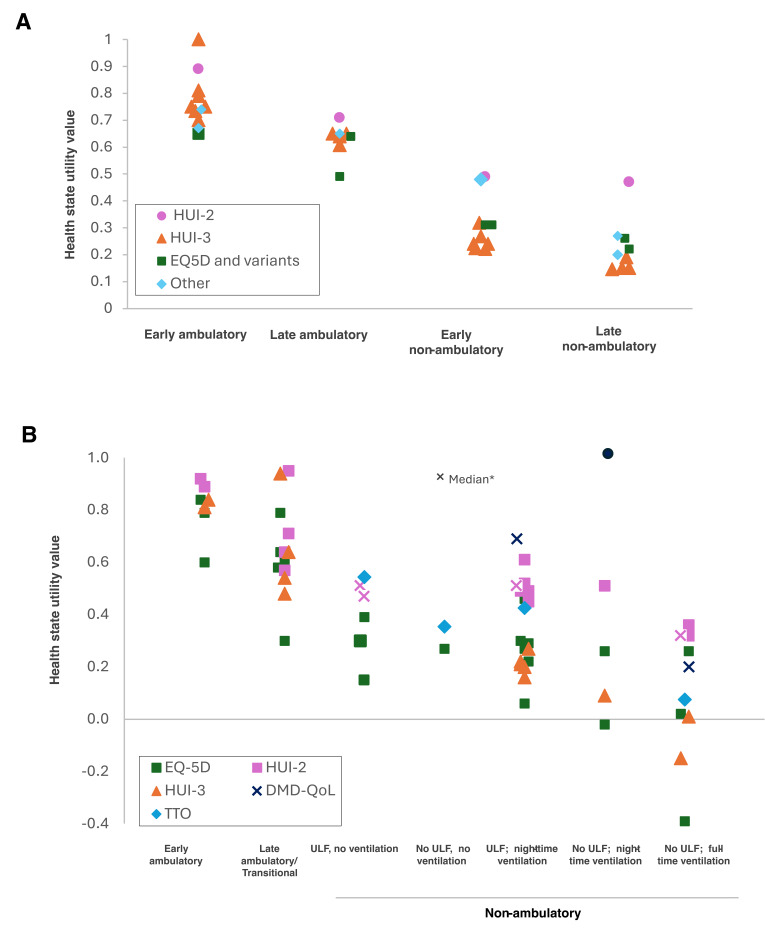
HSUV Data from Identified Publications Reporting Across Health States Reported HSUVs plotted to DMD health states using (**A**) 4 health states; (**B**) 7 health states; note late ambulatory and transitional health states from the 8-stage NHM have been combined; progression through nonambulatory stages may not be the same for each patient. Abbreviations: DMD-QoL, Duchenne Muscular Dystrophy Quality of Life (tool); EQ-5D, EuroQol 5 Dimension; HSUV, health state utility value; HUI, Health Utility Index; QoL, quality of life; TTO, time trade-off; ULF, upper limb function. *Median HSUV values are denoted with an *x* in the same color as the instrument used; all other plotted HSUVs are mean values. Note: These plotted HSUVs are reported from heterogeneous studies using a range of methodologies and varying health state definitions; the figures should be interpreted with this in mind.

To look at the impact of DMD progression on HSUVs, we plotted the reported HSUVs to health states based on the 4-stage and 8-stage models (**Figure 2**). Overall, studies showed decreasing utilities as patients progressed through health states in both the 4-state and 8-state models (**Figure 2A** and **2C,** respectively); however, the HSUVs for each health state were heterogeneous between studies (**Figure 2**). The heterogeneity may reflect differences in patient populations and study designs. Most studies were based on a cross-sectional design and did not assess the same patients through different health states, in addition to the variation in use of patient or proxy individuals to inform HSUVs, and the use of several different instruments to derive the HSUVs. It is important to note these factors when interpreting **Figure 2**. HSUVs mapped to the 4-health-state model according to the utility measure used is shown in **Figure 2A**. While there was heterogeneity between the different instruments used to estimate HSUVs, there was generally consistency within each instrument across health states, including the HUI-3 and EQ-5D, with decreasing utility values corresponding to progression through the 4-state model.

Of the 6 studies reporting HSUVs for caregivers,^17,42–46^ 3 used the EQ-5D or EQ-5D-3L instrument to derive utilities,^42,44,46^ 2 used the HUI-3 instrument,^17,43^ and 1 used TTO-based methodology.^45^ Caregiver HSUVs ranged from 0.71 in an international study of caregivers for adults with DMD using the EQ-5D^42^ to 0.87 in a study of caregivers of patients with DMD across health states in the Netherlands, also using the EQ-5D.^44^ Two studies reported HSUVs for caregivers across DMD health states, both using a 4-health state model,^43,46^ 1 using HUI-3,^43^ and 1 using EQ-5D-3L.^46^ In both studies, HSUVs were slightly lower for caregivers of patients in nonambulatory vs ambulatory health states: With EQ-5D-5L and HUI-3 respectively, mean HSUVs were 0.85 and 0.86 for early ambulatory DMD, 0.83 and 0.84 for late ambulatory, 0.77 and 0.78 for early nonambulatory, and 0.79 and 0.81 for caregivers of patents with late nonambulatory DMD.^43,46^ The one study that specifically reported caregiver HSUVs concluded that caring for a person with DMD is associated with substantial burden and impaired HRQoL.^46^ Half of the caregivers (383/770; 49.7%) reported moderate or extreme anxiety or depression, significantly higher than the rate for the general population of around the same age of approximately 21% (*P* < .001).

All HSUV data can be found in **Supplementary Table S7**. Results from the risk of bias assessment checklist can be found in **Supplementary Table S8.** Overall, the risk of bias was assessed to be low in the identified publications.

### Healthcare Resource Use and Cost

The HCRU and cost SLR was performed in 2 iterations: 1 de novo SLR up to October 2023 and a 2024 update. The findings from each iteration are described below.

For the original SLR (1 January 2014–11 October 2023), 1892 publications were identified, of which 69 publications reporting on 65 unique studies were selected for final inclusion.

In the update to the SLR (12 October 2023–9 January 2024 [aside from EconLit, for which the latest search date was 23 December 2023]), 76 publications were identified, of which 8 publications reporting on 8 unique studies were selected for final inclusion.

Across both iterations of this SLR, a total of 77 publications reporting on 73 unique studies were identified. A combined PRISMA diagram for all iterations is shown in **Figure 1.** These 77 studies were assessed against the study prioritization criteria (**Supplementary Table S6**), leading to a further 39 publications being deprioritized. Thirty-eight studies were prioritized for data extraction.^17,39,42,48–80^ Of these prioritized studies, 9 were multinational, 20 were from the United States, 4 from the United Kingdom, and 1 each from Scotland, Spain, Italy, and Germany.

Of the prioritized publications, 14 reported HCRU only^39,48,56,60,62,66,70,71,73,75,76,78,80^ and 7 reported costs only.^50–53,61,67,74^ An additional 17 publications reported both HCRU and costs.^17,42,48,49,54,55,57–59,63–65,68,69,72,77,79^ The majority of studies included patients with DMD of any age.

### Healthcare Resource Use

**Figure 3.**
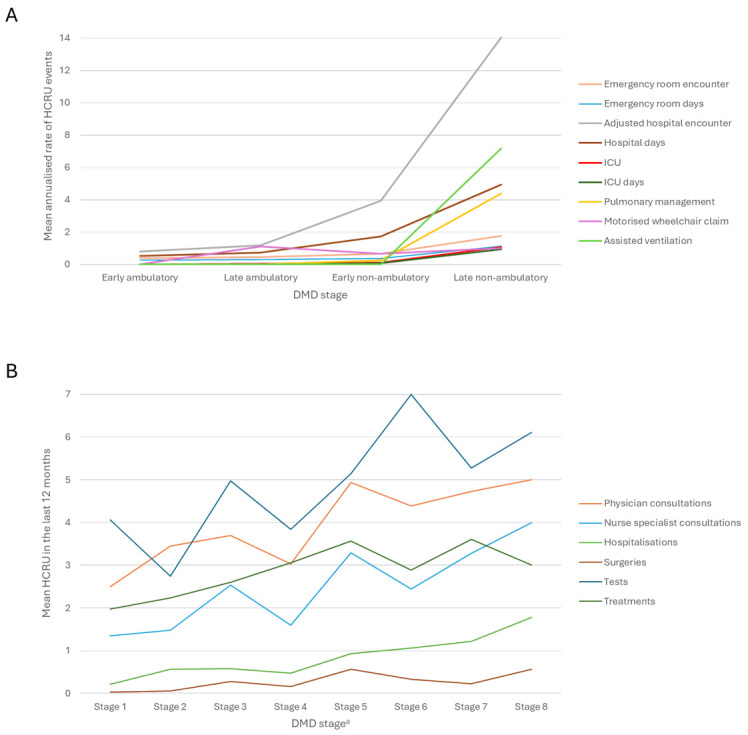
HCRU in 4-Stage and 8-Stage Models of Progressive DMD (**A**) Mean annualized rate of HCRU events as reported by Iff et al^65^ using the 4-state DMD progression model. (**B**) Mean HCRU in the last 12 months from Castro et al^39^ using the 8-stage NHM for DMD progression model.

HCRU was reported by 31 publications,^17,39,42,48,49,54–60,62–66,68–73,75–80^ including 4 that reported HCRU by 4 ambulatory stages^55,65,70,72^ and 1 study that used a more granular 8-stage model.^39^ Two publications reported increases in HCRU as patients progressed from early ambulatory to late nonambulatory DMD.^39,65^ The study by Iff et al^65^ stratified patients by the 4 typical DMD stages and reported small incremental increases in HCRU between early ambulatory, late ambulatory, and early nonambulatory stages but much larger increases from early nonambulatory to late nonambulatory stages (**Figure 3A**). This pattern of increase was seen for emergency room, hospital, and intensive care unit encounters and days spent in each of these settings, as well as HCRU for pulmonary management and ventilation. The study by Castro et al^39^ reported a more gradual increase in HCRU across an 8-stage progression model (**Figure 3B**), but, overall, both studies reported increases in HCRU as the stages of DMD progressed.

Studies highlighting the impact of DMD on the household and caregivers are shown in **Table 2.**^17,42,55,65,71,72,76,77,79^ The impact on patients was the focus of one study, which reported a 97.6% reduction in average working years over their lifetime compared with an average US male citizen,^68^ but the majority of studies reported other factors including the time caregivers spent on informal care duties,^42,71,76,79^ changes in caregiver employment status,^17,71,77^ and proportion of household income dedicated to care costs for a person with DMD.^79^ Four publications reported data on the loss of caregiver working hours or working years over their lifetime,^17,68,72,77^ with only one of these focused on DMD at different stages of progression.^72^ This publication reported substantial amounts of working hours lost by caregivers, with mean losses of 1 hour per week for caregivers of ambulatory patients, 10.7 hours per week for caregivers of patients who had been nonambulatory for 0 to 3 years, and 15 hours per week for caregivers of patients who had been nonambulatory for at least 4 years. In addition, the increased length of time since loss of ambulation can be considered a proxy for other DMD progression stages.^72^

**Table 2.** HCRU Impact of DMD on Households and Caregivers

**Publication (Year)**	**Population**	**Sample Size**	**HCRU**
Innis (2023)^68^	Patients with DMD and their caregivers	N/A	Average lifetime working years per individual: Patients: 0.86 General US male population: 35.78 Difference: 34.93 (97.6%) Caregivers: 20.05 General US population: 24.42 Difference: 4.37 (17.9%)
Cavazza (2016)^42^	Patients of any age with DMD and their caregivers	268 patients; 154 caregivers	Patients’ informal (nonprofessional) caregivers’ hours/week, mean (SD): France: 65.3 (40.1) Germany: 45.3 (67.3) Italy: 59.2 (62.2) Spain: 61.8 (71.7) UK: 62.7 (81.7)
Soelaeman (2021)^72^	Primary, female caregivers of male patients aged <18 years with DMD	86	Labor market outcomes (caregivers): Loss in work hours/week, mean (95% CI): Patients without LOA: -1.0 (3.2, 1.3) Patients with 0-3 years of LOA: -10.7 (-14.8, -6.7) Patients with ≥4 years of LOA: -15.0 (-18.5, -11.5) Loss in annualized work hours, mean (95% CI): Patients without LOA: -1.5 (-111.1, 108) Patients with 0-3 years of LOA: -459.0 (-658.2, -259.8) Patients with ≥4 years of LOA: -809.4 (-977, -641.8)
Flores (2020)^77^	Caregivers of DMD patients who were aged >18 years, 1st or 2nd relatives of patient, and living with patient	36 families of 38 patients	29 (80.5%) households suffered work changes, mothers being the ones making the adjustments in 25 cases and fathers in 21 cases. During previous month, average caregiver had to take 8.83 hours (SD, 13.7; range, 0-76) from work to care for the DMD patient. Approximately 22.2% of households reported that someone in family had to quit working to care for their child, and 17% had to change their job.
Landfeldt (2014)^17^	Patients aged ≥5 years with DMD and their caregivers	770 patient-caregiver pairs	Caregivers in employment, n (%): Germany: 102 (59) Italy: 73 (60) UK: 105 (55) USA: 189 (67) Caregivers with reduced working hours or who had stopped working completely because of their relative’s DMD, n (%): Germany: 74 (43) Italy: 35 (29) UK: 93 (49) USA: 77 (27)
Magliano (2014)^76^	Four 25-year-old patients with DMD who lived with ≥1 relative 18-80 years old	246	Caregivers (parents) in employment, n (%): 133 (54.1) Daily hours in caregiving by parents, mean (SD): 6.3 (4.1) Patients receiving economic welfare benefits, n (%): 177 (72.0) Patients attending rehabilitation programs, n (%) 203 (82.5)
Rodriguez (2022)^79^	Informal caregivers (parents) of children with DMD	74 (40 from Spain)	Hours spent on care per patient, average (mid-range): 44.62 (40) Percentage of annual income related to caregivers’ health expenses (Spain only), mean (SD): Expenses associated with services provided by health professionals for the child: 4.61 (9.61) Expenses related to assistive technology: 8.68 (17.23) Expenses associated with services provided by health professionals for the caregiver: 0.73 (2.05) HCRU, mid-range (%): Family income spent on health professionals for the child: 32.91 Household income spent on assistive technology: 33.63 Family income spent on health professionals for the caregiver: 34.49
Schreiber-Katz (2014)^55^	DMD patients aged ≥16 years and/or their caregivers (parents)	248	Subjectively care-related HCRU of caregivers, % caregivers: Care-related medical problems: 57 Medical treatment needed: 74 Medical rehabilitation needed: 12
Strober (2023b)^71^	Caregivers of patients with DMD	77 (48 Europe, 26 Japan, 3 US)	Hours of DMD care per week, mean (SD): 43.4 (32.2) Proportion of caregivers with a change to their employment due to caring for the patient with DMD: 35.3% Proportion of caregivers reducing working hours: 23.5% Proportion of patients with professional (paid) caregivers: 37.3% Work productivity and activity impairment of caregivers: Overall work impairment: 44.1% Absenteeism: 6.5% Presenteeism: 38.8% Activity impairment: 54.1%

Abbreviations: CI, confidence interval; DMD, Duchenne muscular dystrophy; HCRU, healthcare resource use; LOA, loss of ambulation; N/A, not applicable; SD, standard deviation.

All HCRU data can be found in **Supplementary Table S9**.

### Costs

Costs associated with DMD were reported as direct, indirect ,or total costs across 24 publications.^17,42,49–55,57–59,61,63–65,67–69,72,74,77,79^ Of these, 16 reported direct healthcare costs,^17,42,50–55,57,58,61,63,64,67,69,77^ Ten publications reported indirect costs.^17,42,52,55,61,67–69,72,79^ Sixteen publications reported total costs,^17,42,49,50,52–55,58,59,63–65,69,74^ of which 4 stratified total costs by the typical 4 ambulatory stages.^52,65,67,69^ These 4 publications indicate that total costs increase greatly as DMD progresses, and as with the HCRU data, reported much lower annual total costs for early/late ambulatory DMD, with large increases as DMD progressed to nonambulatory or late nonambulatory stages **Table 3**.^52,65,67,69^

**Table 3.** Summary of Publications Reporting Total Costs, Separated by DMD Ambulatory Stage and Impact on Individuals and the Household

**Publication**	**Population**	**N**	**Currency (Year)**	**Total Costs**
Costs separated by DMD ambulatory stage				
Landfeldt (2017a)^67^	Patients of any age with DMD	N/A	£ (2015)	**Average annual costs/patient in GBP, mean (SE)** Medical costs, Model 1: Initial DMDSAT score (full functional ability): £8340 (830) Per lost score (multiplier): 1.057 (1.005) Medical costs, Model 2: Early ambulatory: £10 670 (140) Late ambulatory: £11 190 (100) Early nonambulatory: £16 490 (290) Late nonambulatory: £27 590 (340) Medical costs, Model 3: No ventilation: £11 520 (60) Nighttime ventilation: £31 710 (590) Day and nighttime ventilation: £36 390 (840) Nonmedical costs, Model 1: Initial DMDSAT score (full functional ability): £9120 (860) Per lost score (multiplier): 1.04 (1.006) Nonmedical costs, Model 2: Early ambulatory: £9 740 (50) Late ambulatory: £11 420 (50) Early nonambulatory: £17 860 (110) Late nonambulatory: £16 810 (90) Nonmedical costs, Model 3: No ventilation: £12 660 (60) Night-time ventilation: £14 610 (240) Day and nighttime ventilation: £15 500 (190)
Iff (2022)^65^	Patients of any age with DMD	938	US$ (2020)	Average per-patient annualized total healthcare costs mean (SD): Early ambulatory: 17 688.48(104 157.74) Late ambulatory: 36 867.81(162 917.88) Early nonambulatory: 72 800.97(342 693.74) Late nonambulatory: 167 284.62(331 378.95) Average annual cost of medical care per patient weighted by the length of stay at each ambulatory stage: $71 451
Mujwara (2022)^69^	DMD patients aged 5 to <18 years	NR	US$ (2020)	Average annual total costs per patient by ambulatory stage: Early/late ambulatory: $18 178 Early nonambulatory: $244 959 Late nonambulatory: $356 619 Average total costs over 13 years: Direct costs: $664 660 Indirect costs: $1 155 662 Direct plus indirect costs: $1820 323
NICE (2016)^52,81^	Ambulatory patients with DMD	NR	£ (2014)	Total health state associated costs per cycle: Ambulatory: £9605 Nonambulatory: £23 600 Nonambulatory and ventilation-assisted: £23 600 Nonambulatory with scoliosis: £25 058-£46 043 Nonambulatory and ventilation-assisted with scoliosis: £25 058-£46 043
Personal and household costs				
Landfeldt (2014)^17^	Patients aged ≥5 years with DMD and their caregivers	770 patient-caregiver pairs	US$ (2012)	Household burden mean (95% CI): Germany: €70 190 (63 760 76 830) Italy: €58 440 (50 200 68 900) UK: £63 600 (58 790 68 370) US: $71 900 (65 520 81 520)
Reynolds (2023)^59^	Patients of any age with DMD and ≥2 medical or pharmaceutical insurance claims	163	US$ (2019)	Total out-of-pocket costs in US$ per 30-day supply median (IQR): Deflazacort (2017 vs 2019): 52 (17-72) vs 63 (39-139) Prednisone (2019): 5 (2-9)
Innis (2023)^68^	Patients with DMD and their caregivers	NR	US$ (NR)	Average income per individual over their lifetime, discounted 3% per annum: Patients: $32 902 US male population: $1 943 462 Difference: $1 910 560 (98.3%) Caregivers: $1 218 359 General US population: $1 383 924 Difference: $165 565 (12.0%)

Abbreviations: CI, confidence interval; DMD, Duchenne muscular dystrophy; DMDSAT, Duchenne Muscular Dystrophy Functional Ability Self-Assessment Tool; HCRU, healthcare resource use; IQR, interquartile range; N/A, not applicable; NR, not reported, SD, standard deviation; SE, standard error.

Studies reporting personal and household costs are also shown in **Table 3**. One study highlighted that males with DMD had a 98.3% reduction in lifetime earnings compared with an average male in the US general population ($32 902 vs $1 943 462, respectively).^68^ Caregivers would experience a 12.0% reduction in lifetime earnings compared with a member of the wider US population ($1 218 359 vs $1 383 924, respectively).^68^

Cost data are shown in **Supplementary Table S9**, and results from the risk of bias assessment checklist for the HCRU and cost publications are shown in **Supplementary Table S10.** Overall, the risk of bias was assessed to be low in the identified publications. Note that cost data were not standardized or inflation-adjusted; this will be done in future modeling and health economic assessments in which the data will be employed.

## DISCUSSION

These SLRs give a broad picture of the burden of DMD for patients, caregivers and the healthcare system, while providing a range of inputs for use in future economic modeling studies.

The HSUV SLR identified a wide range of studies reporting HSUVs for patients with DMD, which showed declining utilities across health states, highlighting the substantial impact on patients’ quality of life as DMD progresses **(Figure 2**). HSUVs were heterogeneous across studies within each health state; however, there was a decline in reported HSUVs across health states for almost every study, suggesting that the variation across studies was due to the heterogeneity in patient populations and study designs, including use of a variety of patient vs proxy respondents and different instruments to measure the utilities.

The number of different HSUV instruments used across studies likely reflects the fact that there is no consensus on the most appropriate instrument for DMD, as none sufficiently characterizes the experiences of patients living with DMD and their caregivers and how they changes over time.^47^ Instruments for assessing HSUVs such as EQ-5D and HUI are typically not specific to the condition being studied; it is therefore important to consider alignment of the instrument’s descriptive system to the condition.^82^ Audhya et al^29^ noted that the HUI-3 provided the broadest range of utility values for DMD compared with HUI-2 and EQ-5D, and it has been reported that the EQ-5D may lack content validity for measurements in DMD.^82^ In light of these findings, we suggest that HSUV measurements in DMD should be performed with HUI-3 as the preferred instrument.

Another factor that may cause differences in HSUV within the same health state is “response shift,” where some patients with DMD accommodate to their symptoms as they learn to adapt to their chronic condition.^13^ For example, a patient who has spent longer in one health state may report better HRQoL than a patient who has spent less time in the same health state. Use of proxy respondents including caregivers, clinicians, and laypersons to gather patient utility data may also have contributed to between-study heterogeneity; for example, layperson estimates may not reflect how a patient feels about aspects of their condition such as losing the ability to walk.^83^ Other studies in DMD HRQoL have reported moderate-to-poor agreement between patient self-reported and carer scores, often caused by different frames of reference, where patients with DMD experience “response shift,” as mentioned above, but parent/carer proxy scores are lower, framed through care-related stress and workload, and concerns about the patient’s decline.^84–86^

Another consideration is the variation in health state definitions and number of health states included across studies, taking note of our conceptual alignment across the different models, as shown in **Supplementary Table S1**. This reflects the evolution in health state definitions over time with more granular health state definitions and increasing numbers of health states included in studies with more recent publication dates. Castro et al,^39^ for example, used an 8-health-state model similar to the NHM described by Broomfield et al.^7^ This model was validated by clinicians and included input from patients and caregivers to capture the natural history of DMD.^7^ The additional stages within the nonambulatory health state provide additional granularity, considering loss of ULF/HTMF, increasing requirement for ventilation support, and development of cardiomyopathy as DMD progresses, and as such, the NHM more closely models the progression of DMD than previous 4-state models of the disease. The health states may not necessarily occur in order, reflecting the variability of an individual patient’s DMD progression after loss of ambulation The non-linearity of DMD progression may be another factor contributing to heterogeneity in reported HSUVs with in a disease state.

Studies identified in the HCRU SLR showed that the HCRU burden also increased as patients progress from early ambulatory to late nonambulatory DMD. In 2 studies that stratified patients into progression stages, there were trends of increased hospital and ICU admissions, and use of mobility aids. However, there were differences in the HCRU levels reported between progression stages, owing to the fact that Iff et al^65^ stratified patients by the 4 typical DMD stages, with a large increase in burden between early and late nonambulatory DMD, whereas Castro et al^39^ reported more gradual HCRU increases between the categories of their 8-stage NHM.

The pattern of cost increases reflected that observed in the studies reporting HCRU with the typical 4-state DMD model: small incremental increases between early ambulatory, late ambulatory, and early nonambulatory stages, with large cost increases at the late nonambulatory stage. Increases in indirect costs were also reported over the 4 health states, indicating the impact of DMD as it progresses on the household and caregivers, as well as patients. In this SLR, no cost studies were identified that employed the NHM model, but the pattern of cost increases may also follow the trends observed with HCRU, with more gradual stepwise increases in costs over the 8 stages of DMD progression.

A future economic model with a societal perspective should take these into account, because the evidence from this SLR indicates that a significant level of informal care is required by patients with DMD,^42,71,76,79^ causing financial toxicity for family members and caregivers, when they reduce their employment hours, retire early or change jobs.^17,68,72,77^ This financial toxicity is exacerbated by additional out-of-pocket costs associated with drug therapies, home modifications, and supportive technology such as mobility aids.^59^

These SLRs identified a wide range of data for HSUVs and HCRU/costs, which can be used to support future economic modeling. However, there was a lack of long-term data, alongside uncertainties and heterogeneity in the data identified. In light of this, future economic models should use the most robust data from the identified studies, selecting larger, high-quality publications to minimize uncertainty. In addition, modeled stages of DMD progression should align with clinician, caregiver, and patient experience, which is a strength of the 8-stage NHM due to the validation conducted with each of these stakeholders.^7^

These SLRs had limitations, including post-hoc prioritization of studies for HTA relevance, English-language-only study inclusion, and no formal publication bias assessment. Although searches were updated through 2024, a targeted January 2026 search identified additional studies but did not change overall conclusions. The search string and results are shown in **Supplementary Section 1.4.** A total of 198 records were identified, of which 5 reported relevant HSUV data,^41,83,87–89^ 3 reported HSUV data,^90–92^ 3 reported cost data^93–95^ and 1 additional study reported NHM-relevant methodology.^96^

New evidence on HSUVs included studies highlighting caregiver burden, variability in utility estimates, and limitations of EQ-5D in DMD.^41,83,87,89^ Comparative analyses supported HUI-3 as the preferred utility measure due to its broader sensitivity across disease stages.^88^ Recent HCRU and cost studies from the United States and Europe confirmed substantial and increasing healthcare and societal costs with DMD progression.^90,92,94^ Additional methodological guidance supported the use of more granular, multi-state NHMs, reinforcing the recommendation for 8-stage modeling in DMD.^96^

## CONCLUSIONS

In conclusion, these SLRs provide a broad picture of the burden of DMD, with data indicating that both patients and caregivers have a substantial utility burden as a consequence of DMD, which increases substantially as DMD progresses. Similarly, there is a high HCRU and cost burden of DMD to patients, caregivers, households and healthcare systems, with the biggest burden also seen at the later, nonambulatory, stages of DMD. New treatments that address the underlying cause of DMD can potentially increase patient and caregiver HRQoL and reduce the socioeconomic burden of DMD in the long term. These SLRs identified a wide range of inputs for economic modeling, as well as providing insights into more granular health state modeling which should be captured in economic models. Future economic modeling of DMD must accommodate the inherent uncertainties due to the heterogeneity of reported utilities, HCRU and costs.

### Disclosures

C.Y. and E.N.K. are employees of F. Hoffmann-La Roche Ltd. C.Y. is also a shareholder of F. Hoffmann-La Roche Ltd. D.S., H.S., and T.M. were employees of Source Health Economics at the time of conducting the SLR. Source Health Economics was funded by F. Hoffmann-La Roche Ltd. to conduct the SLRs and author this publication.

## Supplementary Material

Online Supplementary Material Appendix
