# Supplementary material for: Systematic Literature Reviews of Health-State Utility Values, Costs, and Healthcare Resource Use in Duchenne Muscular Dystrophy
Source: J Health Econ Outcomes Res. 2026 Apr 28;13(1):140–54. doi: 10.36469/001c.158537 (PMC13131909; doi:10.36469/001c.158537)
Supplement: Online Supplementary Material — Appendix [file jheor_2026_13_1_158537_336684.pdf]

Appendix

Table A1. Conceptual mapping of DMD progression models

Early/late DMD staging	Ambulatory / non-ambulatory (numbered)	Brooke score	NHM (Broomfield) stage(s)	Stage description
Early ambulatory	Ambulatory 1	1	NHM stage 1	Individuals who remain independently ambulatory with minimal upper limb impairment
Late ambulatory	Ambulatory 2	2	NHM stage 2	Individuals who remain ambulatory but show functional decline, including compensatory strategies for upper limb elevation
Early non-ambulatory	Non-ambulatory 1	3–4	NHM stages 3–4	Individuals who are wheelchair dependent but retain meaningful upper limb function for activities such as hand-to-mouth tasks. This includes patients who may still perform assisted transfers or brief supported standing and who are not yet ventilator dependent.
Late non-ambulatory	Non-ambulatory 2	5–6	NHM stages 5–8	Individuals with advanced disease characterised by loss of hand-to-mouth function and severe upper limb impairment, accompanied by respiratory involvement requiring night-time or full-time ventilatory support.

Natural History Model (NHM) stages refer to the Broomfield 8-stage model of Duchenne muscular dystrophy progression.[1] NHM stages were mapped to functional disease categories using shared clinical milestones, including loss of ambulation and progressive upper limb functional decline. As the NHM, study stratification terms, and Brooke scores were developed independently and are not formally cross-referenced, mappings represent conceptual and functional alignment rather than exact equivalence.

1.1. Electronic databases included

Table A2 Electronic databases included in the HSUV SLR updates

Database	Platform	2023 SLR update		2024 SLR update	
		Span of search	Date searched	Span of search	Date searched
Embase	Ovid	11th January 2019 to 11th October 2023	12th October 2023	12th October 2023 to 9th January 2024	10th January 2024
MEDLINE Daily, In-Process & Other Non-indexed citations, and e-		11th January 2019 to 11th October 2023	12th October 2023	12th October 2023 to 9th January 2024	10th January 2024

Database	Platform	2023 SLR update		2024 SLR update	
		Span of search	Date searched	Span of search	Date searched
pub ahead-of-print					

HSUV health state utility value, *SLR* systematic literature review.

Table A3 Electronic databases included in the de novo HCRU & cost SLR and SLR update

Database	Platform	2023 de novo SLR		2024 SLR update	
		Span of search	Date searched	Span of search	Date searched
Embase	Ovid	1st January 2014 to 11th October 2023	12th October 2023	12th October 2023 to 9th January 2024	10th January 2024
MEDLINE Daily, In-Process & Other Non-indexed citations, and e-pub ahead-of-print		1st January 2014 to 11th October 2023	12th October 2023	12th October 2023 to 9th January 2024	10th January 2024
CRD – DARE		1st January 2014 to 1st Quarter 2016[a]	12th October 2023	-	-
CRD – HTA database		1st January 2014 to 4th Quarter 2016[a]	12th October 2023	-	-
CRD – NHS EED		1st January 2014 to 1st Quarter 2016[a]	12th October 2023	-	-
EconLit		1st January 2014 to 9th October 2023	12th October 2023	12th October 2023 to 28th December 2023	10th January 2024

[a]The CRD databases are no longer included in the Cochrane library, from 7th August 2018. CRD are maintaining versions of DARE until at least 2021, with records published on DARE until 31st March 2015. More details available at: https://www.crd.york.ac.uk/CRDWeb/ and https://onlinelibrary.wiley.com/doi/abs/10.1002/jrsm.1235.
CRD Centre for Reviews and Dissemination, *DARE* Database of Abstracts of Reviews of Effects, *HCRU* healthcare resource use, *HTA* Health Technology Assessment, *NHS EED* National Health Service Economic Evaluation Database, *SLR* systematic literature review.

Table A4 List of conferences for hand-searching

Short conference name	Full conference name
ISPOR	International Society for Pharmacoeconomics and Outcomes Research Europe and United States of America (USA
HTAi	Health Technology Assessment international

ASGCT	Annual Meeting of the American Society of Gene & Cell Therapy
ESHG	European Society of Human Genetics Conference
AAN	American Academy of Neurology Annual Meeting
MDA	Muscular Dystrophy Association Clinical and Scientific Conference
EAN	European Academy of Neurology
WMS	World Muscle Society
ICNMD	International Congress on Neuromuscular Diseases

Table A5 List of HTA bodies for hand-searching

Country/Region	HTA body
England	National Institute for Health and Care Excellence (NICE)
Scotland	Scottish Medicines Consortium (SMC)
Wales	All Wales Medicines Strategy Group (AWMSG)
Ireland	National Centre for Pharmacoeconomics (NCPE)
Australia	Pharmaceutical Benefits Advisory Committee (PBAC)
Canada	Canadian Agency for Drugs and Technologies in Health (CADTH)
France	Haute Autorité de Santé (HAS)
Germany	German Institute for Quality and Efficiency in Health Care (IQWiG)
	Gemeinsamer Bundesausschuss (The Federal Joint Committee [G-BA])
USA	Institute for Clinical and Economic Review (ICER)

Table A6 Additional inclusion criteria[a]

Characteristics	Inclusion criteria
Country/region	• EU5 (UK, France, Germany, Italy, and Spain), USA, and Canada
Sample size	• ≥30 individuals
Publication type	• Full manuscripts and conference abstracts with posters identified
Date limits	• Studies reporting several relevant data points

[a]These inclusion criteria were applied when a large number of relevant records were identified prior to data extraction.

1.2. Search strings

1.2.1. Health state utility value systematic literature review

October 2023 SLR update

Database: Embase

Platform: Ovid

Date searched: 12/10/2023

Hits: 407

Study design filter: York Health Economics Consortium final search filters 1 (FSF1)[2]

#	Searches	Results
1	exp duchenne muscular dystrophy/	19919
2	(duchenne* or dmd* or nmdmd* or (muscular adj5 dystroph*) or dystrophinopath* or ((pseudo or hypertrophic or myopathic or progressive or pseudohypertrophic) adj3 dystroph*)).mp.	56355
3	1 or 2	56355
4	proxy/ or (caregiver or caregiv* or care-giv* or carer or carer* or caring or parent or mother or father or son or family).mp.	2045465
5	3 and 4	6335
6	3 or 5	56355
7	Quality-Adjusted Life Years/	35499
8	(quality adjusted or adjusted life year$).ti,ab,hw.	47538
9	(qaly$ or qald$ or qale$ or qtime$).ti,ab,hw.	26946
10	(illness state$1 or health state$1).ti,ab,hw.	14684
11	(hui or hui1 or hui2 or hui3).ti,ab,hw.	3187
12	(multiattribute$ or multi attribute$).ti,ab,hw.	1548
13	(utility adj3 (score$1 or valu$ or health$ or cost$ or measur$ or disease$ or mean or gain or gains or index$)).ti,ab,hw.	36442
14	utilities.ti,ab,hw.	15120
15	(eq 5$ or eq-5$ or eq5$ or euro qual or euroqual or euro qual5d$ or euroqual5d$ or euro qol or euroqol or euro qol5d$ or euroqol5d$ or euro quol or euroquol or euro quol5d$ or euroquol5d$ or eur qol or eurqol or eur qol5d$ or eur qol5d$ or eur?qul or eur?qul5d$ or euro$ quality of life or european qol).ti,ab,hw.	35287
16	(euro$ adj3 (5 d or 5d$ or 5-d$ or 5 dimension$ or 5dimension$ or 5 domain$ or 5domain$)).ti,ab,hw.	9545
17	(sf6$ or sf 6$ or sf six or short form 6$ or short form six$).ti,ab,hw.	5249
18	(time trade off$1 or time tradeoff$1 or tto or timetradeoff$1).ti,ab,hw.	3587
19	((qol or hrqol or quality of life).ti,hw. or *quality of life/) and ((qol or hrqol$ or quality of life) adj2 (increas$ or decrease$ or improv$ or declin$ or reduc$ or high$ or low$ or effect or effects or worse or score or scores or change$1 or impact$1 or impacted or deteriorat$)).ab.	180819
20	Cost-Benefit Analysis/ and (cost-effectiveness ratio$ and (perspective$ or life expectanc$)).ti,ab,hw.	1275
21	*quality of life/ and (quality of life or qol).ti.	111626
22	quality of life/ and ((quality of life or qol) adj3 (improv$ or chang$)).ti,ab,hw.	102653
23	quality of life/ and ((quality of life or qol) adj (score$1 or measure$1)).ti,ab,hw.	33498
24	quality of life/ and health-related quality of life.ti,ab,hw.	77203
25	quality of life/ and ec.fs.	61797
26	quality of life/ and (health adj3 status).ti,ab,hw.	36628
27	(quality of life or qol).ti,ab,hw. and Cost-Benefit Analysis/	11157
28	models,economic/	3292

#	Searches	Results
29	or/7-28	442132
30	6 and 29	1072
31	animal/	1635546
32	nonhuman/	7479740
33	exp animal experiment/	3089883
34	exp experimental animal/	824976
35	animal model/	1723068
36	exp rodent/	4044380
37	(rat or rats or mouse or mice).ti.	1615937
38	or/31-37	9990695
39	human/ and 38	2824067
40	38 not 39	7166628
41	30 not 40	1052
42	limit 41 to english language	1011
43	limit 42 to dd=20190111-20231012	163
44	limit 42 to dc=20190111-20231012	407
45	43 or 44	407

Database: MEDLINE, incorporating MEDLINE In-Process & Other non-indexed citations, MEDLINE e-pub ahead-of-print, and MEDLINE Daily

Platform: Ovid

Date searched: 12/10/2023

Hits: 130

Study design filter: York Health Economics Consortium final search filters 1 (FSF1)[2]

#	Searches	Results
1	exp Muscular Dystrophy, Duchenne/	7177
2	(duchenne* or dmd* or nmdmd* or (muscular adj5 dystroph*) or dystrophinopath* or ((pseudo or hypertrophic or myopathic or progressive or pseudohypertrophic) adj3 dystroph*)).mp.	39745
3	1 or 2	39745
4	proxy/ or (caregiver or caregiv* or care-giv* or carer or carer* or caring or parent or mother or father or son or family).mp.	1591419
5	3 and 4	3505
6	3 or 5	39745
7	Quality-Adjusted Life Years/	15850
8	(quality adjusted or adjusted life year$).ti,ab,kf.	23953

#	Searches	Results
9	(qaly$ or qald$ or qale$ or qtime$).ti,ab,kf.	14869
10	(illness state$1 or health state$1).ti,ab,kf.	8509
11	(hui or hui1 or hui2 or hui3).ti,ab,kf.	1999
12	(multiattribute$ or multi attribute$).ti,ab,kf.	1348
13	(utility adj3 (score$1 or valu$ or health$ or cost$ or measur$ or disease$ or mean or gain or gains or index$)).ti,ab,kf.	20466
14	utilities.ti,ab,kf.	9564
15	(eq-5d or eq5d or eq-5 or eq5 or euro qual or euroqual or euro qual5d or euroqual5d or euro qol or euroqol or euro qol5d or euroqol5d or euro quol or euroquol or euro quol5d or euroquol5d or eur qol or eurqol or eur qol5d or eur qol5d or eur?qul or eur?qul5d or euro$ quality of life or european qol).ti,ab,kf.	17738
16	(euro$ adj3 (5 d or 5d or 5 dimension$ or 5dimension$ or 5 domain$ or 5domain$)).ti,ab,kf.	6103
17	(sf36$ or sf 36$ or sf thirtysix or sf thirty six).ti,ab,kf.	26889
18	(time trade off$1 or time tradeoff$1 or tto or timetradeoff$1).ti,ab,kf.	2403
19	((qol or hrqol or quality of life).ti,kf. or *quality of life/) and ((qol or hrqol$ or quality of life) adj2 (increas$ or decrease$ or improv$ or declin$ or reduc$ or high$ or low$ or effect or effects or worse or score or scores or change$1 or impact$1 or impacted or deteriorat$)).ab.	54772
20	Cost-Benefit Analysis/ and (cost-effectiveness ratio$ and (perspective$ or life expectanc$)).ti,ab,kf.	5298
21	*quality of life/ and (quality of life or qol).ti.	64572
22	quality of life/ and ((quality of life or qol) adj3 (improv$ or chang$)).ti,ab,kf.	42286
23	quality of life/ and ((quality of life or qol) adj (score$1 or measure$1)).ti,ab,kf.	15823
24	quality of life/ and health-related quality of life.ti,ab,kf.	45528
25	quality of life/ and ec.fs.	10876
26	quality of life/ and (health adj3 status).ti,ab,kf.	11906
27	(quality of life or qol).ti,ab,hw. and Cost-Benefit Analysis/	21400
28	models,economic/	11089
29	(utility loss$ or disutilit$ or short form$ or shortform$ or SF-12 or SF12 or 15-d or 15d or fifteen-d or fifteend or 15 dimension* or fifteen dimension* or sf-6 or sf6 or sf-6d or sf6d or sf-six or sfsix or sf-sixd or sfsixd or 6 dimension* or six dimension*).ti,ab,kf.	60041
30	(Euroqol5 or EQoL-5D or EQol5 or EQol5D or EQ5D3L or sf-20 or sf20 or sf-16 or sf16 or sf-8 or sf8).ti,ab,kf.	1031
31	or/7-30	260368
32	6 and 31	345
33	animals/	7331458
34	exp animals, laboratory/	953198
35	exp animal experimentation/	10364
36	exp models, animal/	643344
37	exp rodentia/	3561157

#	Searches	Results
38	(rat or rats or mouse or mice).ti.	1447843
39	or/33-38	7447367
40	humans/ and 39	2212982
41	39 not 40	5234385
42	32 not 41	331
43	limit 42 to english language	311
44	limit 43 to ed=20190111-20231012	108
45	limit 43 to dt=20190111-20231012	122
46	44 or 45	130

January 2024 SLR update

Database: Embase

Platform: Ovid

Date searched: 10/01/2024

Hits: 30

Study design filter: York Health Economics Consortium final search filters 1 (FSF1)[2]

#	Searches	Results
1	exp duchenne muscular dystrophy/	20218
2	(duchenne* or dmd* or nmdmd* or (muscular adj5 dystroph*) or dystrophinopath* or ((pseudo or hypertrophic or myopathic or progressive or pseudohypertrophic) adj3 dystroph*)).mp.	57069
3	1 or 2	57069
4	proxy/ or (caregiver or caregiv* or care-giv* or carer or carer* or caring or parent or mother or father or son or family).mp.	2074308
5	3 and 4	6422
6	3 or 5	57069
7	Quality-Adjusted Life Years/	36382
8	(quality adjusted or adjusted life year$).ti,ab,hw.	48799
9	(qaly$ or qald$ or qale$ or qtime$).ti,ab,hw.	27618
10	(illness state$1 or health state$1).ti,ab,hw.	15022
11	(hui or hui1 or hui2 or hui3).ti,ab,hw.	3261
12	(multiattribute$ or multi attribute$).ti,ab,hw.	1585
13	(utility adj3 (score$1 or valu$ or health$ or cost$ or measur$ or disease$ or mean or gain or gains or index$)).ti,ab,hw.	37291
14	utilities.ti,ab,hw.	15414
15	(eq 5$ or eq-5$ or eq5$ or euro qual or euroqual or euro qual5d$ or euroqual5d$ or euro qol or euroqol or euro qol5d$ or euroqol5d$ or euro quol or euroquol or	36422

#	Searches	Results
	euro quol5d$ or euroquol5d$ or eur qol or eurqol or eur qol5d$ or eur qol5d$ or eur?qul or eur?qul5d$ or euro$ quality of life or european qol).ti,ab,hw.	
16	(euro$ adj3 (5 d or 5d$ or 5-d$ or 5 dimension$ or 5dimension$ or 5 domain$ or 5domain$)).ti,ab,hw.	9758
17	(sf6$ or sf 6$ or sf six or short form 6$ or short form six$).ti,ab,hw.	5318
18	(time trade off$1 or time tradeoff$1 or tto or timetradeoff$1).ti,ab,hw.	3653
19	((qol or hrqol or quality of life).ti,hw. or *quality of life/) and ((qol or hrqol$ or quality of life) adj2 (increas$ or decrease$ or improv$ or declin$ or reduc$ or high$ or low$ or effect or effects or worse or score or scores or change$1 or impact$1 or impacted or deteriorat$)).ab.	185342
20	Cost-Benefit Analysis/ and (cost-effectiveness ratio$ and (perspective$ or life expectanc$)).ti,ab,hw.	1307
21	*quality of life/ and (quality of life or qol).ti.	113817
22	quality of life/ and ((quality of life or qol) adj3 (improv$ or chang$)).ti,ab,hw.	105204
23	quality of life/ and ((quality of life or qol) adj (score$1 or measure$1)).ti,ab,hw.	34207
24	quality of life/ and health-related quality of life.ti,ab,hw.	78918
25	quality of life/ and ec.fs.	63449
26	quality of life/ and (health adj3 status).ti,ab,hw.	37255
27	(quality of life or qol).ti,ab,hw. and Cost-Benefit Analysis/	11305
28	models,economic/	3429
29	or/7-28	452514
30	6 and 29	1105
31	animal/	1645917
32	nonhuman/	7580812
33	exp animal experiment/	3130369
34	exp experimental animal/	837287
35	animal model/	1752485
36	exp rodent/	4087173
37	(rat or rats or mouse or mice).ti.	1628097
38	or/31-37	10104097
39	human/ and 38	2872848
40	38 not 39	7231249
41	30 not 40	1084
42	limit 41 to english language	1042
43	limit 42 to dd=20231012-20240110	16
44	limit 42 to dc=20231012-20240110	30
45	43 or 44	30

Database: MEDLINE, incorporating MEDLINE In-Process & Other non-indexed citations, MEDLINE e-pub ahead-of-print, and MEDLINE Daily

Platform: Ovid

Date searched: 10/01/2024

Hits: 12

Study design filter: York Health Economics Consortium final search filters 1 (FSF1) [2]

#	Searches	Results
1	exp Muscular Dystrophy, Duchenne/	7297
2	(duchenne* or dmd* or nmdmd* or (muscular adj5 dystroph*) or dystrophinopath* or ((pseudo or hypertrophic or myopathic or progressive or pseudohypertrophic) adj3 dystroph*)).mp.	40123
3	1 or 2	40123
4	proxy/ or (caregiver or caregiv* or care-giv* or carer or carer* or caring or parent or mother or father or son or family).mp.	1610722
5	3 and 4	3538
6	3 or 5	40123
7	Quality-Adjusted Life Years/	16057
8	(quality adjusted or adjusted life year$).ti,ab,kf.	24540
9	(qaly$ or qald$ or qale$ or qtime$).ti,ab,kf.	15183
10	(illness state$1 or health state$1).ti,ab,kf.	8654
11	(hui or hui1 or hui2 or hui3).ti,ab,kf.	2041
12	(multiattribute$ or multi attribute$).ti,ab,kf.	1390
13	(utility adj3 (score$1 or valu$ or health$ or cost$ or measur$ or disease$ or mean or gain or gains or index$)).ti,ab,kf.	20879
14	utilities.ti,ab,kf.	9732
15	(eq-5d or eq5d or eq-5 or eq5 or euro qual or euroqual or euro qual5d or euroqual5d or euro qol or euroqol or euro qol5d or euroqol5d or euro quol or euroquol or euro quol5d or euroquol5d or eur qol or eurqol or eur qol5d or eur qol5d or eur?qul or eur?qul5d or euro$ quality of life or european qol).ti,ab,kf.	18219
16	(euro$ adj3 (5 d or 5d or 5 dimension$ or 5dimension$ or 5 domain$ or 5domain$)).ti,ab,kf.	6273
17	(sf36$ or sf 36$ or sf thirtysix or sf thirty six).ti,ab,kf.	27216
18	(time trade off$1 or time tradeoff$1 or tto or timetradeoff$1).ti,ab,kf.	2441
19	((qol or hrqol or quality of life).ti,kf. or *quality of life/) and ((qol or hrqol$ or quality of life) adj2 (increas$ or decrease$ or improv$ or declin$ or reduc$ or high$ or low$ or effect or effects or worse or score or scores or change$1 or impact$1 or impacted or deteriorat$)).ab.	56046
20	Cost-Benefit Analysis/ and (cost-effectiveness ratio$ and (perspective$ or life expectanc$)).ti,ab,kf.	5427
21	*quality of life/ and (quality of life or qol).ti.	65023
22	quality of life/ and ((quality of life or qol) adj3 (improv$ or chang$)).ti,ab,kf.	43506
23	quality of life/ and ((quality of life or qol) adj (score$1 or measure$1)).ti,ab,kf.	16123
24	quality of life/ and health-related quality of life.ti,ab,kf.	46415

#	Searches	Results
25	quality of life/ and ec.fs.	10880
26	quality of life/ and (health adj3 status).ti,ab,kf.	12117
27	(quality of life or qol).ti,ab,hw. and Cost-Benefit Analysis/	21674
28	models,economic/	11105
29	(utility loss$ or disutilit$ or short form$ or shortform$ or SF-12 or SF12 or 15-d or 15d or fifteen-d or fifteend or 15 dimension* or fifteen dimension* or sf-6 or sf6 or sf-6d or sf6d or sf-six or sfsix or sf-sixd or sfsixd or 6 dimension* or six dimension*).ti,ab,kf.	61062
30	(Euroqol5 or EQoL-5D or EQol5 or EQol5D or EQ5D3L or sf-20 or sf20 or sf-16 or sf16 or sf-8 or sf8).ti,ab,kf.	1044
31	or/7-30	265461
32	6 and 31	357
33	animals/	7371128
34	exp animals, laboratory/	955512
35	exp animal experimentation/	10401
36	exp models, animal/	645790
37	exp rodentia/	3580278
38	(rat or rats or mouse or mice).ti.	1456289
39	or/33-38	7489117
40	humans/ and 39	2228401
41	39 not 40	5260716
42	32 not 41	343
43	limit 42 to english language	323
44	limit 43 to ed=20231012-20240110	8
45	limit 43 to dt=20231012-20240110	11
46	44 or 45	12

1.2.2. Healthcare resource use and cost systematic literature review

October 2023 de novo SLR

Database: Embase

Platform: Ovid

Date searched: 12/10/2023

Hits: 1,310

Study design filter: based on an adapted version of the McMaster 'Costs – Medline' filter[3]

#	Searches	Results
1	exp duchenne muscular dystrophy/	19919

#	Searches	Results
2	(duchenne* or dmd* or nmdmd* or (muscular adj5 dystroph*) or dystrophinopath* or ((pseudo or hypertrophic or myopathic or progressive or pseudohypertrophic) adj3 dystroph*)).mp.	56355
3	1 or 2	56355
4	exp "cost of illness"/	21358
5	exp "health care cost"/	343080
6	exp productivity/	50296
7	exp health care Utilisation/	96069
8	(cost* or resource* or expense* or employment or productivity).tw.	1750274
9	((drug* or healthcare or health care or health resource or health service or health services) adj3 (use* or utili?ation)).ti,ab.	385989
10	(cost adj3 illness).ti,ab.	3792
11	absenteeism.ti,ab. or exp absenteeism/	23087
12	presenteeism.ti,ab. or exp presenteeism/	3654
13	or/4-12	2289404
14	3 and 13	2282
15	animal/	1635546
16	nonhuman/	7479740
17	exp animal experiment/	3089883
18	exp experimental animal/	824976
19	animal model/	1723068
20	exp rodent/	4044380
21	(rat or rats or mouse or mice).ti.	1615937
22	or/15-21	9990695
23	human/ and 22	2824067
24	22 not 23	7166628
25	14 not 24	2030
26	limit 25 to english language	1961
27	limit 26 to yr="2014-Current"	1310

Database: MEDLINE, incorporating MEDLINE In-Process & Other non-indexed citations, MEDLINE e-pub ahead-of-print, and MEDLINE Daily

Platform: Ovid

Date searched: 12/10/2023

Hits: 544

Study design filter: based on an adapted version of the McMaster 'Costs – Medline' filter [3]

#	Searches	Results
1	exp Muscular Dystrophy, Duchenne/	7177

#	Searches	Results
2	(duchenne* or dmd* or nmdmd* or (muscular adj5 dystroph*) or dystrophinopath* or ((pseudo or hypertrophic or myopathic or progressive or pseudohypertrophic) adj3 dystroph*)).mp.	39745
3	1 or 2	39745
4	exp "Cost of Illness"/	33535
5	exp Health Care Costs/	72202
6	(cost* or resource* or expense* or employment or productivity).tw.	1357144
7	((drug* or healthcare or health care or health resource or health service or health services) adj3 (use* or utili?ation)).ti,ab.	269443
8	(cost adj3 illness).ti,ab.	2483
9	exp Absenteeism/	9791
10	absenteeism.ti,ab.	7254
11	exp Presenteeism/	598
12	presenteeism.ti,ab.	1822
13	or/4-12	1631239
14	3 and 13	1059
15	animals/	7331458
16	exp animals, laboratory/	953198
17	exp animal experimentation/	10364
18	exp models, animal/	643344
19	exp rodentia/	3561157
20	(rat or rats or mouse or mice).ti.	1447843
21	or/15-20	7447367
22	humans/ and 21	2212982
23	21 not 22	5234385
24	14 not 23	922
25	limit 24 to english language	886
26	limit 25 to yr="2014-Current"	544

Database: Centre for Reviews and Dissemination (CRD) databases - Database of Abstracts of Reviews of Effects (DARE), Health Technology Assessment Database (HTAD), and National Health Service Economic Evaluation Database (NHS EED)

Platform: Ovid

Date searched: 12/10/2023

Study design filter: none

Hits: 27

#	Searches	Results
1	exp Muscular Dystrophy, Duchenne/	6
2	(duchenne* or dmd* or nmdmd* or (muscular adj5 dystroph*) or dystrophinopath* or ((pseudo or hypertrophic or myopathic or progressive or pseudohypertrophic) adj3 dystroph*)).mp.	35
3	1 or 2	35
4	limit 3 to english language	31
5	limit 4 to yr="2014-Current"	27

Database: EconLit

Platform: Ovid

Date searched: 28/09/2023

Study design filter: none

Hits: 11

#	Searches	Results
1	(duchenne* or dmd* or nmdmd* or (muscular adj5 dystroph*) or dystrophinopath* or ((pseudo or hypertrophic or myopathic or progressive or pseudohypertrophic) adj3 dystroph*)).mp.	17
2	limit 1 to english	17
3	limit 2 to yr="2014-Current"	11

January 2024 SLR update

Database: Embase

Platform: Ovid

Date searched: 10/01/2024

Hits: 53

Study design filter: based on an adapted version of the McMaster 'Costs – Medline' filter[3]

#	Searches	Results
1	exp duchenne muscular dystrophy/	20218
2	(duchenne* or dmd* or nmdmd* or (muscular adj5 dystroph*) or dystrophinopath* or ((pseudo or hypertrophic or myopathic or progressive or pseudohypertrophic) adj3 dystroph*)).mp.	57069
3	1 or 2	57069
4	exp "cost of illness"/	21517
5	exp "health care cost"/	347255
6	exp productivity/	50854

#	Searches	Results
7	exp health care Utilisation/	98024
8	(cost* or resource* or expense* or employment or productivity).tw.	1787424
9	((drug* or healthcare or health care or health resource or health service or health services) adj3 (use* or utili?ation)).ti,ab.	392194
10	(cost adj3 illness).ti,ab.	3857
11	absenteeism.ti,ab. or exp absenteeism/	23326
12	presenteeism.ti,ab. or exp presenteeism/	3729
13	or/4-12	2333599
14	3 and 13	2335
15	animal/	1645917
16	nonhuman/	7580812
17	exp animal experiment/	3130369
18	exp experimental animal/	837287
19	animal model/	1752485
20	exp rodent/	4087173
21	(rat or rats or mouse or mice).ti.	1628097
22	or/15-21	10104097
23	human/ and 22	2872848
24	22 not 23	7231249
25	14 not 24	2078
26	limit 25 to english language	2008
27	limit 26 to dd=20231012-20240110	24
28	limit 26 to dc=20231012-20240110	53
29	27 or 28	53

Database: MEDLINE, incorporating MEDLINE In-Process & Other non-indexed citations, MEDLINE e-pub ahead-of-print, and MEDLINE Daily

Platform: Ovid

Date searched: 10/01/2024

Hits: 23

Study design filter: based on an adapted version of the McMaster ‘Costs – Medline’ filter [3]

#	Searches	Results
1	exp Muscular Dystrophy, Duchenne/	7297
2	(duchenne* or dmd* or nmdmd* or (muscular adj5 dystroph*) or dystrophinopath* or ((pseudo or hypertrophic or myopathic or progressive or pseudohypertrophic) adj3 dystroph*)).mp.	40123

#	Searches	Results
3	1 or 2	40123
4	exp "Cost of Illness"/	33822
5	exp Health Care Costs/	72511
6	(cost* or resource* or expense* or employment or productivity).tw.	1385004
7	((drug* or healthcare or health care or health resource or health service or health services) adj3 (use* or utili?ation)).ti,ab.	273339
8	(cost adj3 illness).ti,ab.	2518
9	exp Absenteeism/	9815
10	absenteeism.ti,ab.	7346
11	exp Presenteeism/	608
12	presenteeism.ti,ab.	1846
13	or/4-12	1662375
14	3 and 13	1081
15	animals/	7371128
16	exp animals, laboratory/	955512
17	exp animal experimentation/	10401
18	exp models, animal/	645790
19	exp rodentia/	3580278
20	(rat or rats or mouse or mice).ti.	1456289
21	or/15-20	7489117
22	humans/ and 21	2228401
23	21 not 22	5260716
24	14 not 23	943
25	limit 24 to english language	907
26	limit 25 to ed=20231012-20240110	8
27	limit 25 to dt=20231012-20240110	21
28	26 or 27	23

Database: EconLit

Platform: Ovid

Date searched: 10/01/2023

Study design filter: none

Hits: 0

#	Searches	Results
1	(duchenne* or dmd* or nmdmd* or (muscular adj5 dystroph*) or dystrophinopath* or ((pseudo or hypertrophic or myopathic or progressive or pseudohypertrophic) adj3 dystroph*)).mp.	17
2	limit 1 to english	17
3	limit 2 to yr="2023-Current"	0

1.3. Search and results of 2026 targeted literature review

The following search string was used to interrogate Pubmed on 12th January 2026:

("Duchenne Muscular Dystrophy"[Mesh] OR "Duchenne muscular dystrophy"[tiab] OR DMD[tiab]) AND ("Quality of Life"[Mesh] OR "Health Utilities"[Mesh] OR "Costs and Cost Analysis"[Mesh] OR "Health Care Costs"[Mesh] OR "Health Services"[Mesh] OR "Resource Utilization"[tiab] OR HCRU[tiab] OR cost*[tiab] OR economic*[tiab] OR burden[tiab] OR utility[tiab] OR utilities[tiab] OR "EQ-5D"[tiab] OR "Health Utilities Index"[tiab] OR HUI[tiab]) AND (2024:3000[pdat]) AND (humans[Mesh])

One-hundred and ninety-eight records were identified, of which the following studies were selected for reporting DMD-specific HSUV, HCRU or cost data, and any additional updates to the natural history model:

Search	First Author	Year	Title
HSUV	Domaradzki[4]	2024	Quality of life and caregiving burden associated with parenting a person with Duchenne/Becker muscular dystrophy in Poland
	Xu[5]	2024	Assessing validity of the EQ-5D-5L proxy in children and adolescents with Duchenne muscular dystrophy or spinal muscular atrophy
	Xu[6]	2025	A Qualitative Examination of the Content Validity of the EQ-5D-5L and EQ-5D-Y-3L in Adult and Paediatric Patients With Duchenne Muscular Dystrophy
	Do[7]	2024	Cataloging health state utility estimates for Duchenne muscular dystrophy and related conditions
	Szabo[8]	2025	The EQ-5D and Health Utilities Index for assessing health-related quality-of-life impact in Duchenne muscular dystrophy: Evaluating the relevance and interpretation of descriptive systems from patient and caregiver perspectives.
HCRU	Posner[9]	2025	Real-world treatment and health care utilization among patients with Duchenne muscular dystrophy by race and ethnicity in a Medicaid population
	Rudolfsen[10]	2024	Burden of Disease of Duchenne Muscular Dystrophy in Denmark - A National Register-Based Study of Individuals with Duchenne Muscular Dystrophy and their Closest Relatives
	Morgan[11]	2024	Epidemiology and healthcare resource utilisation associated with Duchenne muscular dystrophy.
Cost	Innis[12]	2025	Household costs in the United States for accommodating functional impairments associated with Duchenne muscular dystrophy: results from a caregiver survey
	Diesing[13]	2025	Epidemiology, disease burden and costs of Duchenne muscular dystrophy in Germany: an observational, retrospective health claims data analysis
	Ghawaa[14]	2025	The Utilization, Reimbursement, and Cost of Targeted Therapies for Duchenne Muscular Dystrophy (DMD) in US Medicaid Programs: A Descriptive Trend Analysis from 2017 to 2022
Other[a]	Broomfield[15]	2024	Modeling the multi-state natural history of rare diseases with heterogeneous individual patient data: A simulation study

[a]The “Other” category includes methodological studies that were identified with additional information on the Natural History Model.

2. Data from all included studies

Table A7 Summary of all HSUV studies

Publication	Study design	Country/countries	Instrument/method used to derive utilities	Population: source, and to whom the utility is relevant	Results<br>Health state and utility value, mean (95% CI) [SD]	
**De novo SLR (n=8)**						
Cavazza, 2016[16]	Cross-sectional	Bulgaria, France, Germany, Hungary, Italy, Spain, Sweden, UK	EQ-5D	Patient - patient	Adults with DMD	0.24 [NR]
					Bulgarian adults with DMD	0.66 [0.08]
					French adults with DMD	0.5 [NR]
					German adults with DMD	0.09 [0.05]
					Hungarian adults with DMD	0.31 [0.03]
					Italian adults with DMD	0.19 [0.37]
					Spanish adults with DMD	-0.17 [0.33]
					Swedish adults with DMD	-0.71 [0.41]
					UK adults with DMD	-0.08 [0.07]
				Caregiver - caregiver	Overall	0.71 [NR]
					Bulgaria	0.51 [0.38]
					France	0.66 [0.36]
					Germany	0.69 [0.34]
					Hungary	0.69 [0.33]
					Italy	0.78 [0.27]
					Spain	0.61 [0.42]
					Sweden	0.71 [0.08]
					UK	0.72 [0.37]
			HUI-3		Early ambulatory	0.75

Publication	Study design	Country/countries	Instrument/method used to derive utilities	Population: source, and to whom the utility is relevant	Results<br>Health state and utility value, mean (95% CI) [SD]	
Landfeldt, 2014[17]	Cross-sectional	Germany, Italy, UK, USA		Caregiver - patient	Late ambulatory	0.65
					Early non-ambulatory	0.24
					Late non-ambulatory	0.15
		Germany			Overall	0.45 (0.41, 0.51)
					Early ambulatory	0.8
					Late ambulatory	0.73
					Early non-ambulatory	0.26
					Late non-ambulatory	0.13
		Italy			Overall	0.52 (0.45, 0.58)
					Early ambulatory	0.89
					Late ambulatory	0.72
					Early non-ambulatory	0.21
					Late non-ambulatory	0.13
		UK			Overall	0.43 (0.39, 0.47)
					Early ambulatory	0.66
					Late ambulatory	0.58
					Early non-ambulatory	0.25
					Late non-ambulatory	0.13
		USA			Overall	0.45 (0.42, 0.49)
					Early ambulatory	0.72
					Late ambulatory	0.63
					Early non-ambulatory	0.24
					Late non-ambulatory	0.18
			EQ-5D-3L		Germany	0.79 (0.76-0.82)

Publication	Study design	Country/countries	Instrument/method used to derive utilities	Population: source, and to whom the utility is relevant	Results<br>Health state and utility value, mean (95% CI) [SD]	
		Germany, Italy, UK, USA		Caregiver - caregiver	Italy	0.84 (0.81-0.86)
					UK	0.82 (0.79-0.84)
					US	0.81 (0.78-0.83)
Landfeldt, 2015[18]	Cross-sectional	UK	HUI-3	Patient/caregiver - patient	Initial score (full functional ability)	0.879 SE: 0.037
					Per lost score (multiplier)	0.905 SE: 1.003
					Early ambulatory	0.699 SE: 0.036
					Late ambulatory	0.607 SE: 0.029
					Early non-ambulatory	0.224 SE: 0.014
					Late non-ambulatory	0.146 SE: 0.01
					No ventilation support	0.518 SE: 0.027
					Night-time ventilation	0.129 SE: 0.017
					day and night-time ventilation	0.051 SE: 0.01
				Caregiver - caregiver	Initial score (full functional ability)	0.862 SE: 0.016
					Per lost score (multiplier)	0.995 SE: 1.001
					Early ambulatory	0.858 SE: 0.017
					Late ambulatory	0.839 SE: 0.017
					Early non-ambulatory	0.784 SE: 0.021
					Late non-ambulatory	0.81 SE: 0.018
					No ventilation support	0.837 SE: 0.014
					Night-time ventilation	0.775 SE: 0.03
					day and night-time ventilation	0.774 SE: 0.033
Landfeldt, 2016a[19]	Cross-sectional	German, Italian, UK, USA	HUI-3	Caregiver - patient	Patient completed PedsQL without caregiver help	0.42
					Patient completed PedsQL with caregiver help	0.52

Publication	Study design	Country/countries	Instrument/method used to derive utilities	Population: source, and to whom the utility is relevant	Results<br>Health state and utility value, mean (95% CI) [SD]	
					Early ambulatory (approximate age 5–7y)	0.75
					Late ambulatory (approximate age 8–11y)	0.65
					Early non-ambulatory (approximate age 12–15y)	0.24
					Late non-ambulatory (approximate 16y of age or older)	0.15
					Non-ambulatory	0.19
					Current health excellent	0.62
					Current health very good	0.5
					Current health good	0.37
					Current health fair	0.22
					Current health poor	0
					Current mental health: Happy and interested in life	0.56
					Current mental health: Somewhat happy	0.36
					Current mental health: Somewhat unhappy	0.22
					Current mental health: Very unhappy or so unhappy that life was not worthwhile	0.04
					On ventilation	0.1
Landfeldt, 2016b[20]	Cross-sectional	Germany, Italy, UK, and USA	EQ-5D-3L	Caregiver - caregiver	Overall	0.81
					Early ambulatory	0.85 (0.82, 0.88)
					Late ambulatory	0.83

Publication	Study design	Country/countries	Instrument/method used to derive utilities	Population: source, and to whom the utility is relevant	Results<br>Health state and utility value, mean (95% CI) [SD]	
					Early non-ambulatory	0.77 (0.74, 0.75)
					Late non-ambulatory	0.79
					Care recipient health, excellent	0.88 (0.85, 0.9)
					Care recipient health, very good	0.83
					Care recipient health, good	0.77
					Care recipient health, fair/poor	0.71
					Care recipient mental health, happy and interested in life	0.84
					Care recipient mental health, somewhat happy	0.79
					Care recipient mental health, somewhat unhappy	0.69
					Care recipient mental health, very unhappy	0.57 (0.39, 0.74)
Landfeldt, 2018[21]	Cross-sectional	UK and USA	HUI-3	Patient/caregiver - patient	Overall	0.36 [0.28]
Magnetta, 2018[22]	Cost-effectiveness analysis	NR	Utility inferred based on heart failure utilities and DMD HRQoL scores	Unknown - patient	NYHA Class I	0.699
					NYHA Class II	0.63
					NYHA Class IIII	0.55
					NYHA Class IV	0.435
Pangalila, 2012[23]	Cross-sectional	Netherlands	EQ-5D	Patient - patient	Overall	0.44 [0.13]
				Caregiver - caregiver	Overall	0.87 [0.17]
**October 2023 SLR update (n=13)**						
Andreozzi, 2022[24]	Cross-sectional	Portugal	EQ-5D-3L	Caregiver - patient	All DMD patients	0.05 [0.33]
					Ambulant patients	0.51 [0.28]

Publication	Study design	Country/countries	Instrument/method used to derive utilities	Population: source, and to whom the utility is relevant	Results<br>Health state and utility value, mean (95% CI) [SD]	
					Non-ambulant patients	-0.05 [0.24]
					Non-ambulant patients on full-time ventilation	-0.2 [0.22]
					Non-ambulant patients with non-full-time ventilation	-0.05 [0.21]
					Non-ambulant patients with no ventilation support	0.03 [0.29]
Audhya, 2022a[25]<br>*Superseded by Audhya 2023b[26]*	Cross-sectional	USA	HUI-2	Patient - patient	Early ambulatory (with preserved upper limb function without ventilation or CM)	Median: 0.96 (IQR: 0.86–0.99)
			HUI-2 and HUI-3	Caregiver - patient	Early ambulatory (with preserved upper limb function without ventilation or CM)	Median: 0.85
			HUI-2	Patient - patient	Non-ambulatory with loss of upper limb function, nighttime and daytime ventilation, and symptomatic CM	Median: 0.38
			EQ-5D-5L	Caregiver - patient	Non-ambulatory with loss of upper limb function, nighttime and daytime ventilation, and symptomatic CM	Median: -0.26
			HUI-3	Patient - patient	Non-ambulatory with preserved upper limb function, and without ventilation or CM	Median: 0.16
			HUI-2	Patient - patient	Non-ambulatory with preserved upper limb function, and without ventilation or CM	Median: 0.61

Publication	Study design	Country/countries	Instrument/method used to derive utilities	Population: source, and to whom the utility is relevant	Results<br>Health state and utility value, mean (95% CI) [SD]	
			NR	Patient - patient	Late ambulatory	Median: 0.67
					Early non-ambulatory	Median: 0.51
					Late non-ambulatory	Median: 0.35 (IQR: 0.34–0.36)
Audhya, 2022b[27]	Cross-sectional	USA	TTO	Layperson - patient	Preserved/mildly impaired ambulatory function	0.74 [0.29]
					Moderately/severely impaired ambulatory function	0.65 [0.33]
					Non-ambulatory with preserved/mildly impaired upper limb function and non-invasive ventilation	0.48 [0.34]
					Non-ambulatory with severe loss of upper limb function and non-invasive ventilation	0.27 [0.31]
Audhya, 2023a[28]	Prospective clinical trial	Argentina, Belgium, Brazil, Canada, Chile, Czech Republic, Denmark, France, Germany, Italy, Japan, Korea, the Netherlands, Norway, Poland, Russian Federation, Spain, Taiwan, and Turkey	HUI-3	Caregiver - patient	Ambulatory patients - Baseline	
					Ambulatory patients - Week 24	0.82 [0.19]
					Ambulatory patients - Week 48 (or early withdrawal)	0.77 [0.24]
			HUI-2		Ambulatory patients - Baseline	0.75 [0.22]
					Ambulatory patients - Week 24	0.87 [0.13]
					Ambulatory patients - Week 48 (or early withdrawal)	0.84 [0.15]
Bever, 2023[29]	Cross-sectional	USA (preference weights from UK)	DMD-QoL	Patient - patient	Ambulatory with preserved upper limb function	Median: 0.77 (IQR: 0.73-0.86)

Publication	Study design	Country/countries	Instrument/method used to derive utilities	Population: source, and to whom the utility is relevant	Results<br>Health state and utility value, mean (95% CI) [SD]	
					Non-ambulatory with mildly impaired upper limb function without daytime ventilation or symptomatic CM	Median: 0.69 (IQR: 0.58-0.82)
					Non-ambulatory with loss of upper limb function, symptomatic CM and night and daytime ventilation state	Median: 0.2 (IQR: -0.20-0.41)
		USA (preference weights from Canada)	HUI-2		Ambulatory with preserved upper limb function	Median: 0.96 (IQR: 0.86-0.99)
					Non-ambulatory with mildly impaired upper limb function without daytime ventilation or symptomatic CM	Median: 0.51 (IQR: 0.44-0.55)
					Non-ambulatory with loss of upper limb function, symptomatic CM and night and daytime ventilation state	Median: 0.32 (IQR: 0.23-0.42)
Crossnohere, 2020[30] *(superseded by Crossnohere 2021)*[31]	Cross-sectional	Australia, Belgium, Canada, France, Italy, Netherlands, UK, and USA	EQ-5D-3L	Patient/caregiver - patient	Early-ambulatory	0.57 (0.52, 0.63)
					Late-ambulatory	0.45 (0.36, 0.55)
					Early non-ambulatory	0.23 (0.19, 0.28)
					Late non-ambulatory	0.18 (0.24, 0.33)
Crossnohere, 2021[31]	Cross-sectional	Australia, Belgium, Canada, Netherlands, UK, and USA<br>(USA tariff applied)	EQ-5D-3L	Patient - patient	Overall	0.4 [0.19]
			EQ-5D-Proxy	Caregiver - patient	Overall	0.27 [0.2]
			EQ-5D-Proxy (patients aged ≥12y), EQ-5D-Y (patients aged ≤11y)	Caregiver - patient	Overall	0.5 [0.35]

Publication	Study design	Country/countries	Instrument/method used to derive utilities	Population: source, and to whom the utility is relevant	Results<br>Health state and utility value, mean (95% CI) [SD]	
			EQ-5D-3L (patients), EQ-5D-Proxy caregivers of patients aged ≥12y), EQ-5D-Y (caregivers of patients aged ≤11y)	Patient/caregiver - patient	Early ambulatory	0.65 [0.17]
					Late ambulatory	0.49 [0.23]
					Early non-ambulatory	0.31 [0.2]
					Late non-ambulatory	0.26 [0.16]
Gallop, 2020[32]	Cross-sectional	UK	TTO	Layperson - patient	HS1: FVC greater than or equal to 50%, no assisted ventilation, with HTMF	0.544
					HS2: FVC greater than or equal to 50%, no assisted ventilation, without HTMF	0.354
					HS3/4: FVC 30% to less than 50%; night-time assisted ventilation, with/without HTMF<br>HS5: FVC less than 30% full-time assisted ventilation, no HTMF	0.425
					HS5: FVC less than 30% full-time assisted ventilation, no HTMF	0.075
				Layperson - caregiver	HS1 (NR)	0.639
					HS5 (NR)	0.393
Landfeldt, 2020[33]	Observational	Sweden	HUI-3	Clinician - patient	Ambulatory treated with ataluren + BSC	0.9315
					Non-ambulatory treated with ataluren + BSC	0.3179

Publication	Study design	Country/countries	Instrument/method used to derive utilities	Population: source, and to whom the utility is relevant	Results<br>Health state and utility value, mean (95% CI) [SD]	
					Non-ambulatory treated with ataluren + BSC	0.2672
					Ambulatory treated with BSC	0.6174
					Non-ambulatory treated with BSC	0.1643
					Non-ambulatory treated with BSC	0.0913
Landfeldt, 2022[34]	Observational	Germany, Ireland, Latvia, Sweden, and the UK	HUI-3	Clinician - patient	Ambulatory treated with ataluren + BSC	1.0000
					Non-ambulatory, not yet requiring ventilation support (pFVC: ≥50%) treated with ataluren + BSC	0.3179
					Non-ambulatory, requiring night-time ventilation support (pFVC: 30%–50%) treated with ataluren + BSC	0.1643
					Non-ambulatory, requiring full-time ventilation support (pFVC: <30%; or FVC <1 liter) treated with ataluren + BSC	-0.0732
					Ambulatory treated with BSC	0.7337
					Non-ambulatory, not yet requiring ventilation support (pFVC: ≥50%) treated with BSC	0.2672
					Non-ambulatory, requiring night-time ventilation support (pFVC: 30%–50%) treated with BSC	0.0913

Publication	Study design	Country/countries	Instrument/method used to derive utilities	Population: source, and to whom the utility is relevant	Results<br>Health state and utility value, mean (95% CI) [SD]	
					Non-ambulatory, requiring full-time ventilation support (pFVC: <30%; or FVC <1 liter) treated with BSC	-0.1163
Rowen, 2021[35]	Observational	UK	EQ-5D-5L	Layperson - layperson	Layperson respondent	0.792
			EQ-5D-5L	Layperson - patient	Participants with both DMD-QoL-8D and EQ-5D-5L data (EQ-5D-5L score)	0.206 [0.346]
			DMD-QoL-8D	Layperson - patient	Participants with both DMD-QoL-8D and EQ-5D-5L data (DMD-QoL-8D score)	0.675 [0.256]
Shehata, 2023[36]	Cross-sectional	Egypt	DMD-QOL	Caregiver - patient	Overall	0.43 [0.31]
					Early-ambulatory	0.67 [0.24]
					Late non-ambulatory	0.2 [0.23]
					Non-ambulatory with confinement to bed	0.21 [0.17]
Szabo, 2023a[37]	Cross-sectional	USA	EQ-5D	Patient - patient	Non-ambulatory with mildly impaired upper limb function, without nighttime/daytime ventilation or symptomatic CM	Median: 0.3 (IQR: 0.24-0.35)
			EQ-5D	Caregiver - patient		Median: 0.15 (IQR: 0.07-0.18)
			HUI-2	Patient - patient		Median: 0.51 (IQR: 0.44-0.52)
			HUI-2	Caregiver - patient		Median: 0.47 (IQR: 0.47-0.55)
Xu, 2023[38]	Cross-sectional	China	EQ-5D-5L	Caregiver - patient	Patients with DMD	0.42 [0.37]

Publication	Study design	Country/countries	Instrument/method used to derive utilities	Population: source, and to whom the utility is relevant	Results<br>Health state and utility value, mean (95% CI) [SD]	
**January 2024 SLR update (n=3)**						
Audhya, 2023b[26]	Cross-sectional	USA	HUI-2	Patient - patient	Early ambulatory	0.89 [0.13]
			HUI-3			0.81 [0.22]
			EQ-5D-5L			0.79 [0.2]
			HUI-2		Early ambulatory - preserved upper limb, no daytime ventilation, without symptomatic CM	0.92 [0.08]
			HUI-3			0.84 [0.2]
			EQ-5D-5L			0.84 [0.13]
			HUI-2		Early ambulatory - mildly impaired upper limb, no daytime ventilation, without symptomatic CM	0.57 [NA]
			HUI-3			0.48 [NA]
			EQ-5D-5L			0.3 [NA]
			HUI-2		Late ambulatory	0.71 [0.24]
			HUI-3			0.64 [0.32]
			EQ-5D-5L			0.64 [0.3]
			HUI-2		Late ambulatory - transitional, preserved upper limb, no daytime ventilation, without symptomatic CM	0.64 [0.22]
			HUI-3			0.54 [0.31]
			EQ-5D-5L			0.59 [0.33]
			HUI-2		Late ambulatory - transitional, mildly impaired upper limb, no daytime ventilation, without symptomatic CM-transitional, preserved upper limb, no daytime ventilation, without symptomatic CM	0.95 [0.07]
			HUI-3			0.94 [0.09]
			EQ-5D-5L			0.79 [0.16]
			HUI-2		Early non-ambulatory	0.49 [0.12]

Publication	Study design	Country/countries	Instrument/method used to derive utilities	Population: source, and to whom the utility is relevant	Results<br>Health state and utility value, mean (95% CI) [SD]	
			HUI-3			0.22 [0.14]
			EQ-5D-5L			0.31 [0.13]
			HUI-2		Early non-ambulatory - preserved upper limb, no daytime ventilation, without symptomatic CM	0.61 [0.11]
			HUI-3			0.16 [0.33]
			EQ-5D-5L			0.46 [0.1]
			HUI-2		Early non-ambulatory - mildly impaired upper limb, no daytime ventilation, without symptomatic CM	0.49 [0.12]
			HUI-3			0.21 [0.12]
			EQ-5D-5L			0.3 [0.14]
			HUI-2		Early non-ambulatory - mildly impaired upper limb, no daytime ventilation, with symptomatic CM	0.45 [0.1]
			HUI-3			0.27 [0.14]
			EQ-5D-5L			0.29 [0.07]
			HUI-2		Late non-ambulatory	0.47 [0.1]
			HUI-3			0.15 [0.15]
			EQ-5D-5L			0.22 [0.15]
			HUI-2		Late non-ambulatory - moderately impaired upper limb, no daytime ventilation, without symptomatic CM	0.49 [0.05]
			HUI-3			0.22 [0.11]
			EQ-5D-5L			0.22 [0.15]
			HUI-2		Late non-ambulatory - moderately impaired upper limb, no daytime ventilation, with symptomatic CM	0.52 [0.05]
			HUI-3			0.2 [0.02]
			EQ-5D-5L			0.27 [0.08]
			HUI-2			0.49 [0.07]

Publication	Study design	Country/countries	Instrument/method used to derive utilities	Population: source, and to whom the utility is relevant	Results<br>Health state and utility value, mean (95% CI) [SD]	
			HUI-3		Late non-ambulatory - moderately impaired upper limb, nighttime and daytime ventilation, without symptomatic CM	0.16 [0.15]
			EQ-5D-5L			0.25 [0.14]
			HUI-2		Late non-ambulatory - loss of upper limb function, no daytime ventilation, without symptomatic CM	0.51 [NA]
			HUI-3			0.09 [NA]
			EQ-5D-5L			0.26 [NA]
			HUI-2		Late non-ambulatory - loss of upper limb function, nighttime and daytime ventilation, without symptomatic CM	0.36 [0.01]
			HUI-3			0.01 [0]
			EQ-5D-5L			0.26 [0.01]
			HUI-2		Late non-ambulatory - loss of upper limb function, nighttime and daytime ventilation, with symptomatic CM	0.32 [0.27]
			HUI-3			-0.15 [0.11]
			EQ-5D-5L			0.02 [0.34]
Castro, 2023[39]	Prospective observational	USA and Spain	EQ-5D-5L	Patient/caregiver - patient	Early ambulatory	0.60
					Late ambulatory	0.62
					Transitional - cannot walk 10m, cannot stand from supine but can remain standing	0.58
					Non-ambulatory - HTMF, no ventilator, unable to stand	0.39
					Non-ambulatory - no HTMF, no ventilator, Brooke >4	0.27
					Non-ambulatory - HTMF, nighttime ventilation, Brooke ≤4	0.06

Publication	Study design	Country/countries	Instrument/method used to derive utilities	Population: source, and to whom the utility is relevant	Results<br>Health state and utility value, mean (95% CI) [SD]	
					Non-ambulatory - no HTMF, nighttime ventilation, Brooke >4	-0.02
					Non-ambulatory - full ventilation, FVC <30%	-0.39
Szabo, 2023b[29]	Prospective observational	USA	EQ-5D-5L	Patient/caregiver - patient	Patients with DMD	0.4 [0.32]
			HUI-2			0.61 [0.21]
			HUI-3			0.39 [0.31]

Table A8 Results of the quality assessment of HSUV studies using the checklist published by Papaioannou et al. (2013) [87] (n=15 full-text publications)

Publication	Audhya, 2023a[28]	Audhya, 2023b[26]	Andreozzi, 2022[24]	Cavazza, 2016[16]	Crossnohere, 2021[31]	Landfeldt, 2014, 2015, 2016[17-19]	Landfeldt, 2018[21]	Landfeldt, 2020[33]	Landfeldt, 2022[34]	Pangalila, 2011[23]	Rowen, 2021[35]	Shehata, 2023[36]	Xu, 2023[38]
1. Sample size ≥100?	0	0	0	1	1	1	1	0	0	1	1	0	1
2. Appropriate and robust respondent selection and recruitment?	1	1	1	1	1	1	1	1	1	1	1	1	1
3. Inclusion/ exclusion criteria clearly specified?	1	1	1	1	1	1	1	1	1	1	0	0	1
4. Response rate ≥60%?	1	1	1	0	1	0	0	1	1	0	1	1	1
5. Reporting of attrition/loss to follow-up? (For longitudinal studies only)	N/A	N/A	N/A	N/A	N/A	N/A	N/A	N/A	N/A	N/A	N/A	N/A	N/A
6. Amount and nature of missing data described?	1	1	1	1	0	1	0	1	1	1	0	1	1
7. Appropriate-ness of measure (based on the review authors' judgment)	1	1	1	1	1	1	1	1	1	1	1	1	1
Total score	5/6	5/6	5/6	5/6	5/6	5/6	4/6	5/6	5/6	5/6	4/6	4/6	6/6

HSUV health state utility value, *N/A* not applicable.

Table A9 Summary of all included HCRU and costs studies

Publication	Patient population	Sample size	Currency (year)	Direct costs (medical and non-medical)	Indirect costs	Total costs and cost drivers	Resource use
Bach, 2015[40]<br>Retrospective cohort analysis	Patients with DMD who were continuously ventilator-dependent	122	USD (NR)	Average personal cares cost per patient<br><br>SNF:<br>- $650 per day<br>- $237,250 per year<br><br>Home LPN/RN:<br>- $45 per hour<br>- $262,000 per year for 16 hours per day<br>- $393,000 per year for 24 hours per day<br><br>RN aides:<br>- $16 per hour<br><br>PASs:<br>- $10 per hour<br>- $30,000 per year for 8 hours per day<br>- $90,000 per year for 24 hours per day<br><br>Respiratory equipment rental:<br>- $800 per month<br>- $9,600 per year<br><br>Average hourly care costs per continuously ventilator-dependent patient:<br><br>Patients using CNVS:<br>- At an institution: $27.08 ($650 per day)<br>- At home with LPN/RN caregivers: $45 + $1.25 (respiratory equipment rental)<br>- At home with LPN/RN aides: $16 + $1.25 (respiratory equipment rental)	–	Total medical and personal care costs, invasive vs. non-invasive management by case:<br>- Case 1 (Pre-CMV to age 19 conventional management, CTMV ages 19-48 conventional management): $7.06 million<br>- Case 2 (Pre-CMV to age 22 non-invasive management, CNVS ages 23-48 non-invasive management): $1.65 million<br>- Case 3 (Pre-CMV to age 27 non-invasive management, CNVS ages 27-43 non-invasive management): $495,760<br>- Case 4 (Pre-CMV to age 24 conventional management, CNVS ages 24-39 non-invasive management): $237,417<br>- Case 5 (Pre-CMV to age 28 conventional to non-invasive management, CNVS ages 28-31 non-invasive management): $204,285<br><br>Total care costs for continuously ventilator-dependent patients:<br><br>Patients using CNVS (n=93):<br>- At an institution: $3,321,500<br>- At home with LPN/RN caregivers: $23,908,230	The 93 CNVS users had 53 Hospitalisations for respiratory complications (0.07 Hospitalisations per year), necessitating 38 intubations for 726 patient-years of CNVS

Publication	Patient population	Sample size	Currency (year)	Direct costs (medical and non-medical)	Indirect costs	Total costs and cost drivers	Resource use
				- At home with PASs: $12 + $1.25 (respiratory equipment rental)<br>- At home with no caregivers: $1.25 (respiratory equipment rental)<br>- At assisted-living residences with PASs: $6.88<br><br>Patients using CTMV:<br>- At an institution: $27.08 ($650 per day)<br>- At home with LPN/RN caregivers: $45<br>- At home with PASs: $10<br><br>Average medical and personal care costs, invasive vs. non-invasive management by case:<br><br>Case 1 (Pre-CMV to age 19 conventional management):<br>- Manual WCs: $8,000<br>- Motorised WCs: $24,000<br>- Miscellaneous equipment: $1,870<br><br>Case 1 (CTMV ages 19-48 conventional management):<br>- LPN/RN: $2,271,360<br>- ARF admission: $28,275<br>- Hospitalisation: $113,099<br>- Manual WCs: $12,000<br>- Motorised WCs: $36,000<br>- Ventilator rental: $96,000<br>- SNF ventilator unit: $4,507,750<br><br>Case 2 (Pre-CMV to age 22 non-invasive management):<br>- Manual WCs: $8,000<br>- Motorised WCs: $24,000<br>- Miscellaneous equipment: $1,330<br>- Ventilator rental: $28,800		- At home with LPN/RN aides: $903,857<br>- At home with PASs: $25,421,739<br>- At assisted-living residences with PASs: $1,638,120<br>- All: $55,193,246<br><br>Patients using CTMV (n=25):<br>- At an institution: $30,557<br>- At home with LPN/RN caregivers: $23,165,820<br>- At home with PASs: $32,850<br>- All: $53,756,470<br><br>Total respiratory equipment costs for continuously ventilator-dependent patients:<br><br>Patients using CNVS (n=93):<br>- At an institution: $3,321,500<br>- At home with LPN/RN caregivers: $1,002,240<br>- At home with LPN/RN aides: $192,960<br>- At home with PASs: $3,460,800<br>- At home with no caregivers: $2,187,810<br>- At assisted-living residences with PASs: $261,120<br><br>Total respiratory equipment and care costs for continuously ventilator-dependent patients:	

Publication	Patient population	Sample size	Currency (year)	Direct costs (medical and non-medical)	Indirect costs	Total costs and cost drivers	Resource use
				- Cough assist: $4,750<br><br>Case 2 (CNVS ages 23-48 non-invasive management):<br>- Waiver: $1,470,000<br>- Hospitalisation: $113,099<br><br>Case 3 (Pre-CMV to age 27 non-invasive management):<br>- Manual WCs: $12,000<br>- Motorised WCs: $36,000<br>- Miscellaneous equipment: $1,410<br>- Ventilator rental: $9,600<br>- Cough assist: $4,750<br><br>Case 3 (CNVS ages 27-43 non-invasive management):<br>- Waiver: $432,000<br><br>Case 4 (Pre-CMV to age 24 conventional management):<br>- Manual WCs: $12,000<br>- Motorised WCs: $36,000<br>- Miscellaneous equipment: $420<br>- ARF admission: $28,275<br>- Hospitalisation: $26,322<br><br>Case 4 (CNVS ages 24-39 non-invasive management):<br>- LPN/RN: $134,400<br><br>Case 5 (Pre-CMV to age 28 conventional to non-invasive management):<br>- Manual WCs: $12,000<br>- Motorised WCs: $36,000<br>- Miscellaneous equipment: $2,100<br>- ARF admission: $28,275<br>- Hospitalisation: $13,160<br>- LTAC: $60,000<br>- Ventilator rental: $19,200		Patients using CNVS (n=93):<br>- At home with LPN/RN caregivers: $24,910,470<br>- At home with LPN/RN aides: $1,096,817<br>- At home with PASs: $28,882,539<br>- At home with no caregivers: $2,187,810<br>- At assisted-living residences with PASs: $1,899,240<br>- All: $62,298,376	

Publication	Patient population	Sample size	Currency (year)	Direct costs (medical and non-medical)	Indirect costs	Total costs and cost drivers	Resource use
				- Cough assist: $4,750<br><br>Case 5 (CNVS ages 28-31 non-invasive management):<br>- Ventilator rental: $28,800			
Cavazza, 2016[16]<br>Cross-sectional	Patients of any age with DMD and their caregivers	268 patients and 154 caregivers	EUR (2012)	Average annual costs/patient in EUR, mean (SD)<br><br>Healthcare costs:<br><br>Drugs:<br>- France: NR<br>- Germany: 75 (127)<br>- Italy: 321 (224)<br>- Spain: 198 (515)<br>- UK: 123 (185)<br><br>Medical tests:<br>- France: 404 (413)<br>- Germany: 121 (85)<br>- Italy: 219 (181)<br>- Spain: 290 (218)<br>- UK: 685 (770)<br><br>Medical visits:<br>- France: 3,322 (2,633)<br>- Germany: 3,342 (2,947)<br>- Italy: 1,863 (2,329)<br>- Spain: 3,549 (3,873)<br>- UK: 2,144 (2,819)<br><br>Hospitalisations:<br>- France: 16,071 (22,728)<br>- Germany: 1,672 (3,118)<br>- Italy: 708 (911)<br>- Spain: 1,382 (4,327)<br>- UK: 535 (1,893)<br><br>Health material:<br>- France: NR<br>- Germany: 14,517 (17,756)<br>- Italy: 6,612 (9,636)	Average annual costs/patient in EUR, mean (SD):<br><br>Healthcare transport:<br>- France: NR<br>- Germany: 52 (260)<br>- Italy: 23 (132)<br>- Spain: 32 (139)<br>- UK: 62 (206)<br><br>Non-healthcare transport:<br>- France: 630 (297)<br>- Germany: 185 (243)<br>- Italy: 150 (202)<br>- Spain: 100 (157)<br>- UK: 356 (1,317)<br><br>Main informal carer:<br>- France: 23,194 (6,054)<br>- Germany: 19,629 (27,787)<br>- Italy: 18,518 (18,012)<br>- Spain: 16,622 (18,330)<br>- UK: 17,123 (20,873)<br><br>Other informal	Average annual total direct healthcare costs/patient, mean (SD):<br>- France: 19,797 (25,774)<br>- Germany: 19,779 (19,012)<br>- Italy: 9,744 (10,002)<br>- Spain: 8,954 (7,442)<br>- UK: 3,887 (5,535)<br><br>Average annual total direct non-healthcare costs/patient, mean (SD):<br>- France: 38,907 (13,342)<br>- Germany: 35,492 (39,922)<br>- Italy: 31,518 (32,408)<br>- Spain: 25,154 (27,056)<br>- UK: 30,771 (36,942)<br><br>Average annual total direct costs (healthcare and non-healthcare)/patient, mean (SD):<br>- France: 58,704 (12,432)<br>- Germany: 55,270 (45,334)<br>- Italy: 41,262 (36,049)<br>- Spain: 34,108 (28,405)<br>- UK: 34,658 (36,393)<br><br>Average annual total cost/patient, mean (SD):<br>- France: 58,704 (12,432)<br>- Germany: 55,270 (45,334)<br>- Italy: 41,547 (35,811)<br>- Spain: 34,603 (28,023)<br>- UK: 34,658 (36,393)<br><br>Cost drivers:	Patients' informal (non-professional) caregivers' hours/week, mean (SD):<br>- France: 65.3 (40.1)<br>- Germany: 45.3 (67.3)<br>- Italy: 59.2 (62.2)<br>- Spain: 61.8 (71.7)<br>- UK: 62.7 (81.7)

Publication	Patient population	Sample size	Currency (year)	Direct costs (medical and non-medical)	Indirect costs	Total costs and cost drivers	Resource use
				- Spain: 3,503 (4,562)<br>- UK: 338 (240)<br><br>Non-healthcare costs:<br><br>Professional carer:<br>- France: NR<br>- Germany: 5,516 (14,943)<br>- Italy: 75 (703)<br>- Spain: 883 (6,665)<br>- UK: 819 (3,477)<br><br>Social services:<br>- France: 4,860 (6,873)<br>- Germany: 2,732 (9,122)<br>- Italy: 655 (1,902)<br>- Spain: 1,493 (3,612)<br>- UK: 579 (1,997)	carers:<br>- France: 10,223 (14,458)<br>- Germany: 7,430 (16,017)<br>- Italy: 12,120 (16,441)<br>- Spain: 6,056 (12,406)<br>- UK: 11,893 (19,119)<br><br>Sick leave:<br>- France: NR<br>- Germany: NR<br>- Italy: 285 (2,659)<br>- Spain: 48 (362)<br>- UK: NR<br><br>Early retirement:<br>- France: NR<br>- Germany: NR<br>- Italy: NR<br>- Spain: 447 (3,019)<br>- UK: NR<br><br>Labor productivity losses (patients):<br>- France: NR<br>- Germany: NR<br>- Italy: 285 (1,330)<br>- Spain: 495 (1,690)<br>- UK: NR	- The main drivers of direct healthcare costs were medical devices or tools for patient mobility, in some countries (for example, Germany and Italy)<br>- The main driver of direct non-healthcare costs was informal care, in all countries	
Conway, 2022[41]<br>Cross-sectional	Patients with DMD with health insurance claims. Projected per-patient	670 estimated individual healthcare encounters	USD (2018)	Average annual costs for ambulatory stage (5-10 years) per patient<br><br>Neuromuscular:<br>- Monitoring: 426<br>- Testing: 318	–	Total costs for ambulatory stage (5-10 years) per patient<br><br>Neuromuscular:<br>- Monitoring: 2,556<br>- Testing: 1,908	Among the 670 total individual health-care encounters, 290 were specialty outpatient visits with an average of 15 visits per year

Publication	Patient population	Sample size	Currency (year)	Direct costs (medical and non-medical)	Indirect costs	Total costs and cost drivers	Resource use
	cumulative costs were for ages 5 to 25 years.			- Intervention (prednisone): 36<br>- Intervention (deflazacort): 85,776<br><br>Rehabilitation: 252<br><br>Respiratory:<br>- Monitoring: 213<br>- Testing: 398<br><br>Cardiac:<br>- Monitoring: 213<br>- Testing: 1,060<br>- Intervention (lisinopril): 3<br><br>Orthopedic and bone heath:<br>- Monitoring: 213<br>- Testing: 249<br><br>Gastrointestinal and nutritional: 522<br><br>Average annual costs for nonambulatory stage (11-24 years):<br><br>Neuromuscular:<br>- Monitoring: 426<br>- Testing: 318<br>- Intervention (prednisone): 56<br>- Intervention (deflazacort): 132,179<br><br>Rehabilitation: 244<br><br>Respiratory:<br>- Monitoring: 426<br>- Testing: 1,293<br><br>Cardiac:<br>- Monitoring: 213<br>- Testing: 1,471<br>- Intervention (lisinopril): 4		- Intervention (prednisone): 109<br>- Intervention (deflazacort): 257,329<br><br>Rehabilitation: 1,514<br><br>Respiratory:<br>- Monitoring: 1,278<br>- Testing: 2,390<br><br>Cardiac:<br>- Monitoring: 1,278<br>- Testing: 6,358<br>- Intervention (lisinopril): 9<br><br>Orthopedic and bone heath:<br>- Monitoring: 1,278<br>- Testing: 1,494<br><br>Gastrointestinal and nutritional: 3,132<br><br>Total costs for nonambulatory stage (11-24 years):<br><br>Neuromuscular:<br>- Monitoring: 5,964<br>- Testing: 4,452<br>- Intervention (prednisone): 785<br>- Intervention (deflazacort): 1.9 million<br><br>Rehabilitation: 3,666<br><br>Respiratory:<br>- Monitoring: 5,964<br>- Testing: 18,102<br>- Interventions: 15,600<br><br>Cardiac:	

Publication	Patient population	Sample size	Currency (year)	Direct costs (medical and non-medical)	Indirect costs	Total costs and cost drivers	Resource use
				Orthopedic and bone heath:<br>- Monitoring: 426<br>- Testing: 212<br><br>Gastrointestinal and nutritional: 522		- Monitoring: 2,982<br>- Testing: 20,588<br>- Intervention (lisinopril): 52<br><br>Orthopedic and bone heath:<br>- Monitoring: 5,964<br>- Testing: 2,970<br>- Intervention: 57,000<br><br>Gastrointestinal and nutritional: 7,308<br><br>Total direct costs for full 20-year period (5-25 years):<br><br>Neuromuscular:<br>- Monitoring: 8,520<br>- Testing: 6,360<br>- Intervention (prednisone): 894<br>- Intervention (deflazacort): 2.1 million<br><br>Rehabilitation: 5,180<br><br>Respiratory:<br>- Monitoring: 7,242<br>- Testing: 20,492<br>- Interventions: 15,600<br><br>Cardiac:<br>- Monitoring: 4,260<br>- Testing: 26,946<br>- Intervention (lisinopril): 61<br><br>Orthopedic and bone heath:<br>- Monitoring: 7,242<br>- Testing: 4,464<br>- Intervention: 57,000<br><br>Gastrointestinal and nutritional: 10,440	

Publication	Patient population	Sample size	Currency (year)	Direct costs (medical and non-medical)	Indirect costs	Total costs and cost drivers	Resource use
						Total expected out-of-pocket payment for full 20-year period (5-25 years):<br><br>Neuromuscular<br>- Monitoring: 1,000<br>- Testing: 2,000<br>- Intervention (prednisone): 789<br>- Intervention (deflazacort): 17,340<br><br>Rehabilitation: 70<br><br>Respiratory<br>- Monitoring: 850<br>- Testing: 543<br><br>Cardiac<br>- Monitoring: 400<br>- Testing: 4,752<br>- Intervention (lisinopril): 41<br><br>Orthopedic and bone heath<br>- Monitoring: 748<br>- Testing: 650<br><br>Gastrointestinal and nutritional: 800<br><br>Total cumulative preventive monitoring (office visits and testing costs: 101,146<br><br>Over a 20-year period, estimated total cumulative cost per patient for selected components of the recommended management of DMD: $174 701 if treated with prednisone and over $2.0 million if treated with deflazacort.	

Publication	Patient population	Sample size	Currency (year)	Direct costs (medical and non-medical)	Indirect costs	Total costs and cost drivers	Resource use
Donaldson, 2021[42]<br>Retrospective chart review	Patients aged 15 years or older with DMD	85	–	–	–	–	Medical characteristics, % (95% CI):<br>- Steroid use sometime: 76.5 (66.0–85.0)<br>- Taking SSRI/anxiolytic: 29.4 (20.0–40.3)<br>- Receiving mental health care: 15.3 (8.4–24.7)<br><br>Community resources, % (95% CI):<br><br>Vocational rehabilitation:<br>- Yes: 3.5 (07–10.0)<br>- No: 51.8 (40.66–62.7)<br>- Not documented: 44.7 (33.9–55.9)<br><br>Personal care attendant:<br>- Yes: 49.4 (38.4–60.5)<br>- No: 30.6 (21.0–41.5)<br>- Not documented: 20.0 (12.1–30.0)<br><br>Patient employment, % (95% CI):<br>- Employed: 12.9 (6.6–22.0)<br>- Volunteering: 8.2 (3.4–16.2)<br>- Not documented: 81.2 (71.2–88.9)
Flores, 2020[43]<br>Cross-sectional	Caregivers of DMD patients who were aged over 18 years old, first or second relatives of the patient, and living with the patient	36 families of 38 patients	EUR (NR)	Monthly medical costs incurred by families, % (n=36):<br>- Any (n=35): 97.2<br><€50: 52.8<br>€50-100: 33.3<br>>€100: 11.1<br><br>Monthly formal care costs incurred by families, n (%) (n=36):<br>- Any: 11 (30.5)<br><€250: 7 (19.4)<br>€250-500: 3 (8.3)	–	–	29 (80.5%) households suffered work changes, mothers being the ones making the adjustments in 25 cases and fathers in 21 cases.<br><br>Employment impact to mothers, n (%):<br>- No change: 10 (30.3)<br>- Quit work: 7 (19.4)<br>- Employment change: 1

Publication	Patient population	Sample size	Currency (year)	Direct costs (medical and non-medical)	Indirect costs	Total costs and cost drivers	Resource use
				>€500: 1 (2.7)<br><br>Average monthly formal care cost per family: €88.06 (SD: 189.2)			(2.8)<br>- Timetable change: 14 (38.9)<br>- Paid holidays request: 7 (19.4)<br>- Unpaid holidays request: 3 (8.3)<br><br>Employment impact to fathers, n (%):<br>- No change: 15 (45.4)<br>- Quit work: 1 (2.8)<br>- Employment change: 5 (13.8)<br>- Timetable change: 13 (36.1)<br>- Paid holidays request: 12 (33.3)<br>- Unpaid holidays request: 7 (19.4)<br><br>During the previous month, the average caregiver had to take 8.83 hours (SD: 13.7, range: 0-76) from work to care for the DMD patient. Approximately 22.2% of households reported that someone in he family had to quit working to care for their child, and 17% had to change their job.
Hurvitz, 2021[44]<br>Cross-sectional	Patients ≥1 year of age with DMD prescribed NIV	29	–	–	–	–	Hospital utilisation in the 12 months preceding the clinic visit, n (%):<br>- Respiratory tract illness: 6 (20.7)<br>- Antimicrobial: 14 (48.3)<br>- Emergency room visit: 7 (24.1)<br>- Hospitalisation: 9 (31.0)<br><br>NIV mode, n (%):<br>- Bilevel positive airway

Publication	Patient population	Sample size	Currency (year)	Direct costs (medical and non-medical)	Indirect costs	Total costs and cost drivers	Resource use
							pressure in spontaneous timed mode: 24 (83)<br>- Volume assured pressure support: 5 (17)<br><br>NIV usage 30-day report, mean (SD):<br>- Night with any use %: 82.8 (32.1)<br>- Nights used >4 h (%): 79.4 (34.6)<br>- Nights used <4 h (%): 20.6 (34.6)<br>- Usage on total nights (hours): 7.4 (3.8)<br>- Usage on nights used (hours): 8.0 (3.5)
Iff, 2022[45]<br>Retrospective cohort study	Patients of any age with DMD	938	USD (2020)	–	–	Average per-patient annualised total healthcare costs, mean (SD):<br>- Early ambulatory: $17,688.48 ($104,157.74)<br>- Late ambulatory: $36,867.81 ($162,917.88)<br>- Early non-ambulatory: $72,800.97 ($342,693.74)<br>- Late non-ambulatory: $167,284.62 ($331,378.95)<br><br>Average annual cost of medical care per patient, weighted by the length of stay at each ambulatory stage: $71,451	Annualised rate of HCRU events by ambulatory stage, mean (SD):<br><br>Adjusted emergency room encounter:<br>- Early ambulatory: 0.38 (0.90)<br>- Late ambulatory: 0.44 (1.55)<br>- Early non-ambulatory: 0.65 (2.37)<br>- Late non-ambulatory: 1.76 (6.68)<br><br>Emergency room days:<br>- Early ambulatory 0.25 (0.53)<br>- Late ambulatory 0.29 (0.95)<br>- Early non-ambulatory 0.38 (1.26)<br>- Late non-ambulatory 1.11 (6.18)<br><br>Adjusted hospital encounter:

Publication	Patient population	Sample size	Currency (year)	Direct costs (medical and non-medical)	Indirect costs	Total costs and cost drivers	Resource use
							- Early ambulatory 0.79 (1.80)<br>- Late ambulatory 1.19 (3.08)<br>- Early non-ambulatory 3.95 (18.17)<br>- Late non-ambulatory 14.05 (104.39)<br><br>Hospital days:<br>- Early ambulatory 0.51 (1.07)<br>- Late ambulatory 0.72 (1.77)<br>- Early non-ambulatory 1.73 (4.54)<br>- Late non-ambulatory 4.95 (11.69)<br><br>Adjusted ICU:<br>- Early ambulatory 0.01 (0.07)<br>- Late ambulatory 0.04 (0.37)<br>- Early non-ambulatory 0.10 (0.71)<br>- Late non-ambulatory 1.09 (9.25)<br><br>ICU days:<br>- Early ambulatory 0.00 (0.05)<br>- Late ambulatory 0.02 (0.21)<br>- Early non-ambulatory 0.09 (0.59)<br>- Late non-ambulatory 0.92 (6.99)<br><br>Pulmonary management:<br>- Early ambulatory 0.00 (0.00)<br>- Late ambulatory 0.00 (0.00)<br>- Early non-ambulatory 0.23 (1.15)<br>- Late non-ambulatory 4.39 (6.64)

Publication	Patient population	Sample size	Currency (year)	Direct costs (medical and non-medical)	Indirect costs	Total costs and cost drivers	Resource use
							Motorised wheelchair claim:<br>- Early ambulatory 0.00 (0.05)<br>- Late ambulatory 1.10 (3.50)<br>- Early non-ambulatory 0.66 (1.39)<br>- Late non-ambulatory 1.01 (1.95)<br><br>Scoliosis:<br>- Early ambulatory 0.00 (0.00)<br>- Late ambulatory 0.00 (0.00)<br>- Early non-ambulatory 0.84 (2.77)<br>- Late non-ambulatory 1.84 (6.30)<br><br>Cardiac management:<br>- Early ambulatory 0.45 (1.12)<br>- Late ambulatory 0.83 (2.21)<br>- Early non-ambulatory 1.45 (2.91)<br>- Late non-ambulatory 2.09 (6.78)<br><br>Tracheostomy:<br>- Early ambulatory 0.00 (0.00)<br>- Late ambulatory 0.00 (0.00)<br>- Early non-ambulatory 0.00 (0.00)<br>- Late non-ambulatory 9.13 (20.58)<br><br>Cough assist device:<br>- Early ambulatory 0.00 (0.00)<br>- Late ambulatory 0.28 (1.64)<br>- Early non-ambulatory 0.33 (1.26)

Publication	Patient population	Sample size	Currency (year)	Direct costs (medical and non-medical)	Indirect costs	Total costs and cost drivers	Resource use
							- Late non-ambulatory 1.16 (2.26)<br><br>Assisted ventilation:<br>- Early ambulatory 0.00 (0.00)<br>- Late ambulatory 0.00 (0.00)<br>- Early non-ambulatory 0.00 (0.00)<br>- Late non-ambulatory 7.18 (11.81)
Iff, 2023[46]<br>Retrospective cohort study	Male patients with DMD <40 years old at diagnosis or initiation of treatment	778	–	–	–	–	Exposure to steroid treatment, n (%):<br>- Eteplirsen-treatment: pre-index period 122 (31.4), follow-up 135 (34.7)<br>- Control: pre-index period 127 (32.6), follow-up 159 (40.8)<br><br>Pre-index period HCRU:<br><br>Length of pre-index period in months, mean (SD):<br>- Eteplirsen treated: 10.57 (1.76)<br>- Control: 10.63 (1.67)<br><br>Hospital encounters:<br>Yearly average care-adjusted number, mean (SD):<br>- Eteplirsen treated: 3.70 (7.75)<br>- Control: 3.73 (7.77)<br>Yearly average days, mean (SD):<br>- Eteplirsen treated: 0.42 (1.09)<br>- Control: 0.39 (1.15)<br><br>ICU:<br>Yearly average care-adjusted

Publication	Patient population	Sample size	Currency (year)	Direct costs (medical and non-medical)	Indirect costs	Total costs and cost drivers	Resource use
							number, mean (SD):<br>- Eteplirsen treated: 0.13 (1.13)<br>- Control: 0.05 (0.51)<br>Yearly average days, mean (SD):<br>- Eteplirsen treated: 0.12 (1.05)<br>- Control: 0.05 (0.43)<br><br>Pulmonary management:<br>Yearly average number, mean (SD):<br>- Eteplirsen treated: 0.96 (3.46)<br>- Control: 0.85 (3.85)<br><br>Motorised wheelchair:<br>Yearly average number, mean (SD):<br>- Eteplirsen treated: 0.82 (2.30)<br>- Control: 0.77 (2.19)<br><br>Ever have Motorised wheelchair, n (%):<br>- Eteplirsen treated: 94 (24.16)<br>- Control: 97 (24.94)<br><br>Scoliosis:<br>Yearly average number, mean (SD):<br>- Eteplirsen treated: 0.92 (6.04)<br>- Control: 0.53 (1.89)<br><br>Cardiac management:<br>Yearly average number, mean (SD):<br>- Eteplirsen treated: 1.49 (2.93)<br>- Control: 1.40 (2.70)

Publication	Patient population	Sample size	Currency (year)	Direct costs (medical and non-medical)	Indirect costs	Total costs and cost drivers	Resource use
							Tracheostomy:<br>Yearly average number, mean (SD):<br>- Eteplirsen treated: 0.98 (11.07)<br>- Control: 0.80 (7.24)<br><br>Cough assist device:<br>Yearly average number, mean (SD):<br>- Eteplirsen treated: 0.41 (1.73)<br>- Control: 0.45 (1.93)<br><br>Assisted ventilation:<br>Yearly average number, mean (SD):<br>- Eteplirsen treated: 0.99 (6.78)<br>- Control: 0.76 (6.41)<br><br>Observed pre-index period and estimated follow-up average rates of HCRU events, mean (SD):<br><br>Care-adjusted hospital encounter:<br>- Observed annualised pre-index period rates: Treated 3.70 (7.75), Control 3.73 (7.77)<br>- Annualised follow-up rates: Treated 4.17 (0.45), Control 6.08 (0.76)<br><br>Care-adjusted ER:<br>- Observed annualised pre-index period rates: Treated 0.71 (2.20), Control 0.65 (2.02)<br>- Annualised follow-up rates:

Publication	Patient population	Sample size	Currency (year)	Direct costs (medical and non-medical)	Indirect costs	Total costs and cost drivers	Resource use
							Treated 0.62 (0.08), Control 0.91 (0.09)<br><br>Assisted ventilation:<br>- Observed annualised pre-index period rates: Treated 0.99 (6.78), Control 0.76 (6.41)<br>- Annualised follow-up rates: Treated 1.87 (0.19), Control 3.06 (0.53)<br><br>Cardiac management:<br>- Observed annualised pre-index period rates: Treated 1.49 (2.93), Control 1.40 (2.70)<br>- Annualised follow-up rates: Treated 1.39 (0.10), Control 1.76 (0.13)<br><br>Cough assist device:<br>- Observed annualised pre-index period rates: Treated 0.41 (1.73), Control 0.45 (1.93)<br>- Annualised follow-up rates: Treated 0.67 (0.08), Control 0.76 (0.10)<br><br>ER days:<br>- Observed annualised pre-index period rates: Treated 0.42 (1.09), Control 0.39 (1.15)<br>- Annualised follow-up rates: Treated 0.36 (0.05), Control 0.53 (0.06)<br><br>Hospital days:<br>- Observed annualised pre-index period rates: Treated 1.97 (4.12), Control 1.82

Publication	Patient population	Sample size	Currency (year)	Direct costs (medical and non-medical)	Indirect costs	Total costs and cost drivers	Resource use
							(3.47)<br>- Annualised follow-up rates: Treated 1.89 (0.18), Control 2.86 (0.35)<br><br>Care-adjusted ICU:<br>- Observed annualised pre-index period rates: Treated 0.13 (1.13), Control 0.05 (0.51)<br>- Annualised follow-up rates: Treated 0.25 (0.05), Control 0.27 (0.07)<br><br>ICU days:<br>- Observed annualised pre-index period rates: Treated 0.12 (1.05), Control 0.05 (0.43)<br>- Annualised follow-up rates: Treated 0.22 (0.04), Control 0.24 (0.06)<br><br>Motorised wheelchair:<br>- Observed annualised pre-index period rates: Treated 0.82 (2.30), Control 0.77 (2.19)<br>- Annualised follow-up rates: Treated 0.94 (0.09), Control 0.96 (0.07)<br><br>Pulmonary management:<br>- Observed annualised pre-index period rates: Treated 0.96 (3.46), Control 0.85 (3.85)<br>- Annualised follow-up rates: Treated 1.33 (0.18), Control 1.98 (0.29)<br><br>Scoliosis:<br>- Observed annualised pre-

Publication	Patient population	Sample size	Currency (year)	Direct costs (medical and non-medical)	Indirect costs	Total costs and cost drivers	Resource use
							index period rates: Treated 0.92 (6.04), Control 0.53 (1.89)<br>- Annualised follow-up rates: Treated 0.99 (0.21), Control 1.24 (0.34)<br><br>Tracheostomy:<br>- Observed annualised pre-index period rates: Treated 0.98 (11.07), Control 0.80 (7.24)<br>- Annualised follow-up rates: Treated 0.90 (0.17), Control 6.50 (3.47)
Iff, 2020[47]<br>Cross-sectional	Patients with DMD	301 etiplirsen-treated and 301 untreated matched patients	–	–	–	–	Health events observed in matched sample at baseline (baseline yearly average rates):<br>DMD-related medical events per patient, mean (SD):<br><br>Primary scenario (>6 months follow-up), eteplirsen-treated patients vs untreated patients:<br>- Adjusted Emergency Room: 0.53 (1.72) vs 0.53 (1.85)<br>- Emergency Room Days: 0.34 (0.99) vs 0.32 (1.27)<br>- Adjusted Hospital Encounter: 3.43 (7.42) vs 3.12 (10.44)<br>- Hospital Days: 1.89 (3.94) vs 1.79 (5.81)<br>- Adjusted ICU: 0.15 (1.26) vs 0.07 (0.73)<br>- ICU Days: 0.14 (1.18) vs 0.06 (0.64)<br>- Pulmonary Management: 0.94 (3.47) vs 0.76 (2.70)<br>- Motorised Wheelchair: 0.82

Publication	Patient population	Sample size	Currency (year)	Direct costs (medical and non-medical)	Indirect costs	Total costs and cost drivers	Resource use
							(2.36) vs 0.83 (2.33)<br>- Scoliosis: 1.04 (6.79) vs 0.85 (5.35)<br>- Cardiac Management: 1.56 (3.13) vs 1.38 (2.89)<br>- Tracheostomy: 0.76 (9.07) vs 0.60 (6.42)<br>- Cough Assist Device 0.44 (1.78) vs 0.28 (1.47)<br>- Assisted Ventilation: 0.78 (4.86) vs 0.58 (4.37)<br><br>Sensitivity scenario (>3 months follow-up), eteplirsen-treated patients vs untreated patients:<br>- Adjusted Emergency Room: 0.52 (1.67) vs 0.42 (1.48)<br>- Emergency Room Days: 0.34 (0.97) vs 0.24 (0.72)<br>- Adjusted Hospital Encounter: 3.39 (7.25) vs 3.56 (10.36)<br>- Hospital Days: 1.89 (3.88) vs 1.88 (5.66)<br>- Adjusted ICU: 0.14 (1.21) vs 0.09 (0.72)<br>- ICU Days: 0.13 (1.14) vs 0.07 (0.62)<br>- Pulmonary Management: 1.05 (3.72) vs 0.97 (4.04)<br>- Motorised Wheelchair: 0.80 (2.30) vs 0.63 (1.92)<br>- Scoliosis: 1.06 (6.61) vs 0.84 (4.65)<br>- Cardiac Management: 1.54 (3.05) vs 1.44 (2.78)<br>- Tracheostomy: 0.70 (8.74) vs 0.70 (5.54)<br>- Cough Assist Device 0.41 (1.72) vs 0.23 (1.25)<br>- Assisted Ventilation: 1.09 (7.35) vs 0.63 (3.90)

Publication	Patient population	Sample size	Currency (year)	Direct costs (medical and non-medical)	Indirect costs	Total costs and cost drivers	Resource use
							Adjusted health events in follow-up periods (yearly average rates based on Poisson model estimates): DMD-related medical events, mean (SE):<br><br>Primary scenario (>6 months follow-up), eteplirsen-treated patients vs untreated patients:<br>- Adjusted Emergency Room: 0.060 (0.009) vs 0.101 (0.012)<br>- Emergency Room Days: 0.038 (0.006) vs 0.057 (0.007)<br>- Adjusted Hospital Encounter: 0.361 (0.045) vs 0.597 (0.106)<br>- Hospital Days: 0.168 (0.019) vs 0.255 (0.025)<br>- Adjusted ICU: 0.023 (0.005) vs 0.054 (0.017)<br>- ICU Days: 0.021 (0.004) vs 0.047 (0.014)<br>- Pulmonary Management: 0.089 (0.011) vs 0.173 (0.034)<br>- Scoliosis: 0.092 (0.021) vs 0.110 (0.018)<br>- Cardiac Management: 0.127 (0.010) vs 0.187 (0.016)<br>- Tracheostomy: 0.072 (0.015) vs 0.351 (0.116)<br>- Assisted Ventilation: 0.124 (0.016) vs 0.210 (0.032)<br><br>Sensitivity scenario (>3 months follow-up), eteplirsen-treated patients vs untreated

Publication	Patient population	Sample size	Currency (year)	Direct costs (medical and non-medical)	Indirect costs	Total costs and cost drivers	Resource use
							patients:<br>- Adjusted Emergency Room: 0.061 (0.009) vs 0.098 (0.019)<br>- Emergency Room Days: 0.035 (0.006) vs 0.065 (0.016)<br>- Adjusted Hospital Encounter: 0.376 (0.048) vs 0.599 (0.105)<br>- Hospital Days: 0.178 (0.020) vs 0.260 (0.030)<br>- Adjusted ICU: 0.023 (0.005) vs 0.039 (0.014)<br>- ICU Days: 0.021 (0.004) vs 0.034 (0.011)<br>- Pulmonary Management: 0.100 (0.013) vs 0.206 (0.033)<br>- Scoliosis: 0.086 (0.020) vs 0.141 (0.027)<br>- Cardiac Management: 0.128 (0.010) vs 0.159 (0.013)<br>- Tracheostomy: 0.064 (0.015) vs 0.358 (0.168)<br>- Assisted Ventilation: 0.145 (0.017) vs 0.261 (0.040)
Innis, 2023[48]<br>Cost of illness analysis	Patients with DMD and their caregivers	–	USD (NR)	–	–	Average income per individual over their lifetime<br><br>Undiscounted:<br>Patients: $57,355<br>General US male population: $6,469,403<br>- Difference: $6,412,047 (99.1%)<br>Caregivers: $2,046,976<br>General US population: $2,325,372<br>- Difference: $278,396 (12.0%)	Average working years per individual over their lifetime:<br><br>Patients: 0.86<br>General US male population: 35.78<br>- Difference: 34.93 (97.6%)<br><br>Caregivers: 20.05<br>General US population: 24.42<br>- Difference: 4.37 (17.9%)

Publication	Patient population	Sample size	Currency (year)	Direct costs (medical and non-medical)	Indirect costs	Total costs and cost drivers	Resource use
						Discounted 3% per annum:<br>Patients: $32,902<br>US male population: $1,943,462<br>- Difference: $1,910,560 (98.3%)<br>Caregivers: $1,218,359<br>General US population: $1,383,924<br>- Difference: $165,565 (12.0%)	
Klimchak, 2021[49]<br>Cross-sectional	Male patients with DMD aged ≤30 years old	3971	USD (2018)	Monthly medical costs per patient, median (IQR)<br><br>Medications:<br>- Commercial $333 ($38-$2,437)<br>- Medicaid $211 ($12-$1,533)<br><br>Inpatient:<br>- Commercial $0 ($0-$117)<br>- Medicaid $0 ($0-$93)<br><br>Outpatient:<br>- Commercial $939 ($382-$1,948)<br>- Medicaid $728 ($192-$2,301)	–	Total direct medical costs per patient, median (IQR):<br>- Commercial: $1,883 ($657-$6,796)<br>- Medicaid: $1,735 ($367-$5,281)	Monthly resource use, median (IQR)<br><br>Distinct medications dispensed:<br>- Commercial: DMD 2.4 (1.5-4.4)<br>- Medicaid: DMD 3.5 (1.9-6.6)<br><br>Any Hospitalisation, n (%):<br>- Commercial: DMD 368 (25.9)<br>- Medicaid: DMD 535 (31.1)<br><br>Hospitalisations among those with ≥1 Hospitalisation:<br>- Commercial: DMD 0.0 (0.0-0.1)<br>- Medicaid: DMD 0.0 (0.0-0.1)<br><br>Length of stay per Hospitalisation (days):<br>- Commercial: DMD 4.0 (2.0-7.0)<br>- Medicaid: DMD 4.3 (2.0-8.2)<br><br>Any ED visit, n(%):

Publication	Patient population	Sample size	Currency (year)	Direct costs (medical and non-medical)	Indirect costs	Total costs and cost drivers	Resource use
							- Commercial: DMD 679 (47.8)<br>- Medicaid: DMD 1,113 (64.6)<br><br>ED visits among those with ≥1 visit:<br>- Commercial: DMD 0.1 (0.0-0.1)<br>- Medicaid: DMD 0.1 (0.0-0.1)<br><br>Outpatient visits:<br>- Commercial: DMD 1.4 (0.7-3.1)<br>- Medicaid: DMD 3.8 (1.3-13.9)<br><br>≥1 GP visit, n (%):<br>- Commercial: DMD 916 (64.5)<br>- Medicaid: DMD 1,179 (68.5), Comparison 2,670 (49.5)<br><br>GP visits among those with ≥1 GP visit:<br>- Commercial: DMD 0.1 (0.0-0.2), Comparison 0.0 (0.0-0.1)<br>- Medicaid: DMD 0.1 (0.0-0.4)<br><br>≥1 specialist visit, n (%):<br>- Commercial: DMD 1,378 (97.0)<br>- Medicaid: DMD 1,293 (75.1)<br><br>Specialist visits among those with ≥1 specialist visit:<br>- Commercial: DMD 0.5 (0.3-0.9)<br>- Medicaid: DMD 0.2 (0.0-0.5)

Publication	Patient population	Sample size	Currency (year)	Direct costs (medical and non-medical)	Indirect costs	Total costs and cost drivers	Resource use
Klimchak, 2023[50]<br>Cost-effectiveness analysis	4-year-old patients with DMD		USD (2021)	Delandistrogene moxeparvovec treatment unit costs:<br>- IV infusion (up to 1 hour): $69.21<br>- IV infusion (additional hours): $21.46<br>- Prednisone for pre-infusion immunosuppressant: $22.79<br>- Laboratory monitoring: $3.00<br><br>Steroid unit costs (comparator):<br>Prednisone/prednisolone: $0.05/mg	–	Maximum discounted* delandistrogene moxeparvovec lifetime treatment cost: $1,038,093<br><br>Total discounted* lifetime direct medical costs (non-treatment costs):<br>- Delandistrogene moxeparvovec: $1,164,783<br>- SOC only: $1,105,932<br>- Incremental: $58,851<br><br>*3% per annum	–
Landfeldt, 2017a[51]<br>Cost-effectiveness analysis	Patients of any age with DMD	–	GBP (2015)	Average annual costs/patient in GBP, mean (SE)<br><br>Medical costs:<br>Model 1:<br>- Initial DMDSAT score (full functional ability): 8,340 (830)<br>- Per lost score (multiplier): 1.057 (1.005)<br><br>Model 2:<br>- Early ambulatory: 10,670 (140)<br>- Late ambulatory: 11,190 (100)<br>- Early non-ambulatory: 16,490 (290)<br>- Late non-ambulatory: 27,590 (340)<br><br>Model 3:<br>- No ventilation: 11,520 (60)<br>- Night-time ventilation: 31,710 (590)<br>- Day- and night-time ventilation: 36,390 (840)<br><br>Non-medical costs:<br>Model 1:<br>- Initial DMDSAT score (full	Average annual costs/patient, mean in GBP (SE):<br><br>Patients' productivity:<br>Model 1:<br>- Initial DMDSAT score (full functional ability): 14,230 (1,540)<br>- Per lost score (multiplier): NA<br><br>Model 2:<br>- Early ambulatory: 0 (0)<br>- Late ambulatory: 0 (0)<br>- Early non-ambulatory: 0 (0)<br>- Late non-ambulatory: 14,230 (1,540)<br><br>Model 3:<br>- No ventilation:	–	–

Publication	Patient population	Sample size	Currency (year)	Direct costs (medical and non-medical)	Indirect costs	Total costs and cost drivers	Resource use
				functional ability): 9,120 (860)<br>- Per lost score (multiplier): 1.04 (1.006)<br><br>Model 2:<br>- Early ambulatory: 9,740 (50)<br>- Late ambulatory: 11,420 (50)<br>- Early non-ambulatory: 17,860 (110)<br>- Late non-ambulatory: 16,810 (90)<br><br>Model 3:<br>- No ventilation: 12,660 (60)<br>- Night-time ventilation: 14,610 (240)<br>- Day- and night-time ventilation: 15,500 (190)	14,230 (1,540)<br>- Night-time ventilation: 14,230 (1,540)<br>- Day- and night-time ventilation: 14,230 (1,540)<br><br>Caregivers' productivity:<br>Model 1:<br>- Initial DMDSAT score (full functional ability): 6,360 (740)<br>- Per lost score (multiplier): 1.037 (1.006)<br><br>Model 2:<br>- Early ambulatory: 7,180 (190)<br>- Late ambulatory: 8,340 (150)<br>- Early non-ambulatory: 12,810 (370)<br>- Late non-ambulatory: 11,240 (260)<br><br>Model 3:<br>- No ventilation: 9,160 (120)<br>- Night-time ventilation: 10,490 (420)<br>- Day- and night-time ventilation: 12,860 (640)		

Publication	Patient population	Sample size	Currency (year)	Direct costs (medical and non-medical)	Indirect costs	Total costs and cost drivers	Resource use
Landfeldt, 2014[17]<br><br>Cross-sectional	Patients aged ≥5 years with DMD and their caregivers	770 patient-caregiver pairs	USD (2012)	Per-patient annual costs in USD, mean (95% CI)<br><br>Hospital admissions (emergency and respite care):<br>- Germany (n=173): 2,080 (1,020, 4,950)<br>- Italy (n=122): 1,420 (900, 2,470)<br>- UK (n=191): 2,300 (1,500, 3,720)<br>- USA (n=284): 2,220 (900, 5,050)<br><br>Visits to physicians and other healthcare practitioners:<br>- Germany: 3,850 (3,410, 4,340)<br>- Italy: 2,590 (1,970, 3,440)<br>- UK: 8,230 (6,360, 13,150)<br>- USA: 18,210 (15,450, 22,260)<br><br>Tests and assessments:<br>- Germany: 2,400 (2,180, 2,680)<br>- Italy: 600 (530, 690)<br>- UK: 1,580 (1,450, 1,750)<br>- USA: 2,860 (2,660, 3,070)<br><br>Medications:<br>- Germany: 1,020 (770, 2,000)<br>- Italy: 1,550 (890, 4,650)<br>- UK: 930 (820, 1,070)<br>- USA: 2,070 (1,720, 2,710)<br><br>Aids, devices, and investments (e.g. home adaptations for wheelchair accessibility):<br>- Germany: 5,560 (4,160, 7,460)<br>- Italy: 1,850 (970, 4,450)<br>- UK: 7,520 (5,690, 9,790)<br>- USA: 7,930 (6,210, 10,260)<br><br>Per-patient annual household costs, mean (95% CI):<br><br>Total out-of-pocket payments:	Per-patient annual costs in USD, mean (95% CI):<br><br>Non-medical community services (home help, personal assistants, nannies, and transportation services):<br>- Germany: 8,920 (6,890, 12,400)<br>- Italy: 2,740 (1,640, 5,380)<br>- UK: 19,250 (13,240, 28,670)<br>- USA: 7,610 (6,030, 9,790)<br><br>Informal care:<br>- Germany: 18,530 (16,440, 20,580)<br>- Italy: 13,160 (11,270, 15,280)<br>- UK: 14,340 (13,030, 15,990)<br>- USA: 13,370 (12,060, 14,930)<br><br>Intangible costs (costs due to pain, anxiety, social handicap, etc.):<br>- Germany: 45,160 (40,650, 49,850)<br>- Italy: 37,830 (30,220, 41,760)<br>- UK: 45,770 (42,070, 49,670)	Total per-patient annual costs in USD, mean (95% CI):<br><br>Direct costs:<br>- Germany: 42,360 (38,640, 46,880)<br>- Italy: 23,920 (20,420, 28,300)<br>- UK: 54,160 (47,310, 63,510)<br>- USA: 54,270 (48,740, 62,220)<br><br>Direct and indirect costs:<br>- Germany: 63,140 (57,600, 69,710)<br>- Italy: 42,140 (36,940, 47,730)<br>- UK: 72,870 (64,350, 84,150)<br>- USA: 75,820 (69,350, 85,270)<br><br>Intangible costs:<br>- Germany: 45,860 (41,630, 50,160)<br>- Italy: 37,980 (32,400, 43,550)<br>- UK: 46,080 (42,360, 50,050)<br>- USA:45,080 (41,100, 48,260)<br><br>Burden of illness:<br>- Germany: 109,000 (100,390, 119,510)<br>- Italy: 80,120 (71,030, 89,190)<br>- UK: 118,950 (108,280, 132,710)<br>- USA: 120,910 (111,460, 130,770)	Caregivers in employment, n (%):<br>- Germany: 102 (59)<br>- Italy: 73 (60)<br>- UK: 105 (55)<br>- USA: 189 (67)<br><br>Caregivers with reduced working hours or who had stopped working completely because of their relative's DMD, n (%):<br>- Germany: 74 (43)<br>- Italy: 35 (29)<br>- UK: 93 (49)<br>- USA: 77 (27)

Publication	Patient population	Sample size	Currency (year)	Direct costs (medical and non-medical)	Indirect costs	Total costs and cost drivers	Resource use
				- Germany: 5,940 (4,240, 8,990)<br>- Italy: 7,550 (3,600, 16,470)<br>- UK: 3,490 (2,220, 5,570)<br>- USA: 14,390 (10,300, 22,970)<br><br>Insurance premiums:<br>- Germany: 150 (60, 290)<br>- Italy: 10 (0, 30)<br>- UK: 10 (0, 30)<br>- USA: 6,210 (2,820, 14,580)<br><br>Copayments for medical services:<br>- Germany: 90 (60, 140)<br>- Italy: 1,160 (130, 450)<br>- UK: 60 (30, 140)<br>- USA: 930 (750, 1,140)<br><br>Copayments for medications:<br>- Germany: 490 (240, 1,450)<br>- Italy: 1,490 (350, 4,440)<br>- UK: 100 (60, 140)<br>- USA: 1,470 (1,120, 2,070)<br><br>Copayments for community services:<br>- Germany: 380 (190, 870)<br>- Italy: 650 (300, 2,480)<br>- UK: 140 (60, 290)<br>- USA: 710 (360, 1,630)<br><br>Out-of-pocket payments for investments:<br>- Germany: 4,830 (3,150, 7,670)<br>- Italy: 4,250 (480, 2,350)<br>- UK: 3,180 (2,020, 5,710)<br>- USA: 5,060 (3,130, 8,540)	- USA: 45,080 (41,100, 48,260)<br><br>Productivity losses:<br>- Germany: 20,770 (17,670, 24,250)<br>- Italy: 18,220 (15,430, 21,380)<br>- UK: 18,700 (16,280, 21,150)<br>- USA: 21,550 (18,490, 24,720)<br><br>Per-patient annual household costs, mean (95% CI):<br><br>Income loss:<br>- Germany: 1,190 (730, 1,880)<br>- Italy: 620 (310, 1,130)<br>- UK: 750 (440, 1,200)<br>- USA: 840 (500, 1,360)<br><br>Loss of leisure time:<br>- Germany: 17,910 (16,210, 20,110)<br>- Italy: 12,440 (10,710, 14,980)<br>- UK: 13,590 (12,410, 14,980)<br>- USA: 11,700 (10,520, 12,630)	Household burden:<br>- Germany: 70,190 (63,760, 76,830)<br>- Italy: 58,440 (50,200, 68,900)<br>- UK: 63,600 (58,790, 68,370)<br>- USA: 71,900 (65,520, 81,520)<br><br>Estimated national burden of illness using the most recent DMD prevalence estimates:<br>- Germany: 278,058,000<br>- Italy: 154,465,000<br>- UK: 200,478,000<br>- USA: 1,217,373,000	

Publication	Patient population	Sample size	Currency (year)	Direct costs (medical and non-medical)	Indirect costs	Total costs and cost drivers	Resource use
Landfeldt, 2015[52]<br>Cross-sectional	Patients aged ≥5 years with DMD and their caregivers	770 patient-caregiver pairs	–	–	–	–	Patients with ventilation support, n (%):<br>- Germany (n=173): 26 (15)<br>- Italy (n=122): 24 (20)<br>- UK (n=191): 35 (18)<br>- USA (n=284): 41 (14)<br><br>Visit to GP or pediatrician during the last six months, n (%):<br>- Germany: 115 (66)<br>- Italy: 42 (34)<br>- UK: 119 (62)<br>- USA: 171 (60)<br><br>Patients taking glucocorticoids who had visited a neuromuscular specialist during the last six months, n (%):<br>- Germany: 56 (72)<br>- Italy: 21 (27)<br>- UK: 81 (68)<br>- USA: 131 (62)<br><br>Patients with scoliosis who had visited a physiotherapist during the last six months, n (%):<br>- Germany: 62 (80)<br>- Italy: 25 (50)<br>- UK: 29 (55)<br>- USA: 38 (48)<br><br>Patients with scoliosis that had visited an orthopedist during the last six months, n (%):<br>- Germany: 29 (37)<br>- Italy: 9 (18)<br>- UK: 12 (23)<br>- USA: 23 (29)

Publication	Patient population	Sample size	Currency (year)	Direct costs (medical and non-medical)	Indirect costs	Total costs and cost drivers	Resource use
							Patients meeting guidelines for pulmonary evaluations (including FVC and peak cough flow, once every six months at a minimum for non-ambulatory patients), n (%):<br>- Germany: 76 (81)<br>- Italy: 37 (66)<br>- UK: 51 (61)<br>- USA: 78 (62)<br><br>Patients with access to any orthosis, n (%):<br>- Germany: 50 (29)<br>- Italy: 79 (65)<br>- UK: 125 (65)<br>- USA: 199 (70)<br><br>Patients with access to standing devices, n (%):<br>- Overall (n=770): 93 (12)<br>- Early ambulatory patients (n=155): 0 (0)<br>- Late ambulatory patients (n=256): 15 (6)<br>- Early non-ambulatory patients (n=154): 50 (32)<br>- Late non-ambulatory patients (n=205): 28 (14)<br><br>Wheelchair access among late ambulatory patients, n (%):<br>- Germany (n=49): 27 (55)<br>- Italy (n=35): 9 (26)<br>- UK (n=62): 47 (76)<br>- USA (n=110): 64 (58)<br><br>Approximately 315 (88%) of all non-ambulatory patients reported having access to a power wheelchair

Publication	Patient population	Sample size	Currency (year)	Direct costs (medical and non-medical)	Indirect costs	Total costs and cost drivers	Resource use
							Patients meeting all absolute care recommendations, % (95% CI):<br>- Germany: 25 (18, 31)<br>- Italy: 9 (4, 14)<br>- UK: 37 (30, 44)<br>- USA: 30 (25, 35)<br><br>Patients meeting all absolute care recommendations, n (%):<br>- Early ambulatory patients: 76 (49)<br>- Late ambulatory patients: 76 (30)<br>- Early non-ambulatory patients: 24 (16)<br>- Late non-ambulatory patients: 33 (16)
Landfeldt, 2017b[53]<br>Cost of illness analysis	Patients with DMD	NA	EUR (2012)	–	–	Total annual mortality cost of DMD, mean (95% CI) million EUR:<br>- Germany: 335 (334, 337)<br>- Italy: 248 (247, 250)<br>- UK: 267 (266, 269)<br>- USA: 1,208 (1,202, 1,214)<br><br>Total annual societal economic burden of DMD, mean (95% CI) million EUR:<br>- Germany: 613 (591, 640)<br>- Italy: 403 (385, 420)<br>- UK: 468 (450, 491)<br>- USA: 2,425 (2,330, 2,525)<br><br>Total annual societal economic burden of DMD per patient in EUR, mean:<br>- Germany: 240,000<br>- Italy: 209,000	–

Publication	Patient population	Sample size	Currency (year)	Direct costs (medical and non-medical)	Indirect costs	Total costs and cost drivers	Resource use
						- UK: 278,000<br>- USA: 241,000	
Lang, 2019[54]<br>Restrospective, single-centre study	Patients with DMD	405	USD (NR)	Unit costs:<br>- MRI cardiac with and without contrast: $351.74<br>- MRI cardiac without contrast: $270.77<br><br>Estimated average cost per CMR study:<br>- Conservative cohort (conservative GBCA administration policy adopted from September 2017): $339<br>- Historical cohort (treated between September 2016 - August 2017): $351<br><br>Estimated average cost per CMR study in final 7 months of analysis (conservative cohort): $324	–	–	Initiation of new cardiac medication, n (%):<br>- Conservative cohort (conservative GBCA administration policy adopted from September 2017): 45 (24%)<br>- Historical cohort (treated between September 2016 - August 2017): 48 (22%)<br><br>New or increased dosage of cardiac medication, n (%)<br>- Conservative cohort: 73 (39%)<br>- Historical cohort: 68 (31%)<br><br>Total scan time, minutes:<br>- Conservative cohort: 35.3 (+/- 9.02)<br>- Historical cohort: 39.0 (+/- 8.08)
Lin, 2019 (updated 2022)[55]<br>Cost-utility analysis	Patients with DMD	–	USD (2018)	Drug costs per mg:<br>- Prednisone: $0.05<br>- Deflazacort: $6.19<br>- Eteplirsen: $16<br><br>Annual treatment cost per patient:<br>- Prednisone (0.75mg/kg/day): $550<br>- Deflazacort (0.9mg/kg/day): $81,400<br>- Eteplirsen (30 mg/kg/week): $1,002,000	Adverse event costs per patient per year, base-case cost [range used in PSA (50-150%)]:<br>- Cataracts: $75 [$38-$113]<br>- Cataract Surgery: $3,434 [$1,717-$5,152]<br>- Weight Gain: $75 [$38-$113]<br>- Cushingoid: $75 [$38-$113]<br>- Behavioral Change: $75 [$38-$113]	–	–

Publication	Patient population	Sample size	Currency (year)	Direct costs (medical and non-medical)	Indirect costs	Total costs and cost drivers	Resource use
					- Fractures: $7,661 [$3,831-$11,492]		
Magliano, 2014[56]<br>Cross-sectional	4-25 year old patients with DMD who lived with at least one relative 18-80 years old	246	–	–	–	–	Caregivers (parents) in employment, n (%): 133 (54.1)<br><br>Daily hours in caregiving by parents, mean (SD): 6.3 (4.1)<br><br>Patients receiving economic welfare benefits, n (%): 177 (72.0)<br><br>Patients on drug treatment, n (%): 288 (84.6)<br>- Corticosteroids: 168 (68.3)<br>- Bone metabolism drugs: 92 (37.4)<br>- Cardiologic drugs: 80 (32.5)<br>- Gastric drugs: 48 (19.5%)<br>- Neurological drugs: 5 (2.0)<br>- Pulmonary drugs: 4 (1.6)<br><br>Patients attending rehabilitation programs, n (%) 203 (82.5)
Mujwara, 2022[57]<br>Cost of illness analysis	DMD patients aged 5 to <18 years	–	USD (2020)	Average cost per patient in USD (10th-90th percentile):<br>- 12 months pre-LOA: 74,744 (21,000-110,000)<br>- 12 months post-LOA: 106,460 (29,000-160,000)<br><br>Average increase in cost per patient 12 months post-LOA vs 12 months pre-LOA: 63,043<br><br>Average costs per patient over 13 years, without a hypothetical treatment that delayed LOA by 4 years vs with the treatment:	Average increase in cost in USD per patient 12 months post-LOA vs 12 months pre-LOA: 163,738<br><br>Average annual costs per patient over 13 years, without a hypothetical treatment that delayed LOA by 4 years vs with the	Average annual total costs in USD per patient by ambulatory stage:<br>- Early/late ambulatory: 18,178<br>- Early non-ambulatory: 244,959<br>- Late non-ambulatory: 356,619<br><br>Average total costs over 13 years, without a hypothetical treatment that delayed LOA by 4 years vs with the treatment:	Average work loss per patient (hours):<br>- Without treatment: 3,465<br>- With treatment: 935<br>- Incremental: 2,530

Publication	Patient population	Sample size	Currency (year)	Direct costs (medical and non-medical)	Indirect costs	Total costs and cost drivers	Resource use
				Facility inpatient:<br>- Without treatment: 23,199<br>- With treatment: 13,261<br>- Incremental: 9,938<br><br>Facility Outpatient:<br>- Without treatment: 146,833<br>- With treatment: 118,445<br>- Incremental: 28,388<br><br>Professional:<br>- Without treatment: 49,653<br>- With treatment: 44,995<br>- Incremental: 4,658<br><br>Other:<br>- Without treatment: 284,136<br>- With treatment: 84,832<br>- Incremental: 199,304<br><br>Other Prescription Drugs:<br>- Without treatment: 8,663<br>- With treatment: 8,230<br>- Incremental: 433<br><br>Outpatient Pharmacy:<br>- Without treatment: 22,869<br>- With treatment: 16,745<br>- Incremental: 6,125<br><br>Office Administered Drugs:<br>- Without treatment: 2,368<br>- With treatment: 939<br>- Incremental: 1,429<br><br>Ambulance:<br>- Without treatment: 17,482<br>- With treatment: 6,103<br>- Incremental: 11,380<br><br>Durable Medical Equipment:<br>- Without treatment: 109,457	treatment:<br><br>Loss of income:<br>- Without treatment: 103,589<br>- With treatment: 28,107<br>- Incremental: 75,482<br><br>Intangible costs (reduced QoL):<br>- Without treatment: 518,128<br>- With treatment: 160,823<br>- Incremental: 357,304<br><br>Loss in leisure time:<br>- Without treatment: 153,833<br>- With treatment: 41,740<br>- Incremental: 112,093<br><br>Non-medical costs (e.g., aids, investments):<br>- Without treatment: 104,265<br>- With treatment: 28,290<br>- Incremental: 75,974<br><br>Non-medical	Direct costs:<br>- Without treatment: 664,660<br>- With treatment: 356,072<br>- Incremental: 308,589<br><br>Indirect costs:<br>- Without treatment: 1,155,662<br>- With treatment: 333,807<br>- Incremental: 821,856<br><br>Direct plus indirect costs:<br>- Without treatment: 1,820,323<br>- With treatment: 689,878<br>- Incremental: 1,130,444<br><br>Scenario analysis - average total cost savings over 13 years, with a hypothetical treatment that delayed LOA by 2 years vs without the treatment:<br>- Direct costs: 98,244<br>- Indirect costs: 327, 477	

Publication	Patient population	Sample size	Currency (year)	Direct costs (medical and non-medical)	Indirect costs	Total costs and cost drivers	Resource use
				- With treatment: 62,521<br>- Incremental: 46,936<br><br>Sub-total:<br>- Without treatment: 664,660<br>- With treatment: 356,072<br>- Incremental: 308,589	community services:<br>- Without treatment: 100,057<br>- With treatment: 27,149<br>- Incremental: 72,909<br><br>Informal care:<br>- Without treatment: 175,791<br>- With treatment: 47,698<br>- Incremental: 128,093		
NICE, 2016 (HST3)[58]<br>Cost-consequence analysis	Ambulatory patients with DMD	–	GBP (2014)	Ataluren list price:<br>- £2,532 per box of 30 x 125mg sachets<br>- 30 x 250mg sachets: £5,064<br>- 30 x 1000mg sachets: £20,256<br><br>Costs of treatment/patient:<br>- Price of the technology (average 8 year old, weighing 26kg): £675.20 per day; £246,448 per year<br>- Administration cost: £0<br>- Training cost: £0<br>- Other costs (monitoring, tests etc): £0<br><br>Health state associated direct costs per cycle:<br>Ambulatory: £1,633<br>Non-ambulatory: £4,012<br>Non-ambulatory and ventilation-assisted: £4,012<br>Non-ambulatory with scoliosis: £4,012	Health-state-associated indirect costs per cycle:<br>- Ambulatory: £7,972<br>- Non-ambulatory: £19,588<br>- Non-ambulatory and ventilation-assisted: £19,588<br>- Non-ambulatory with scoliosis: £19,588<br>- Non-ambulatory and ventilation-assisted with scoliosis: £19,588	Total health state associated costs per cycle:<br>- Ambulatory: £9,605<br>- Non-ambulatory: £23,600<br>- Non-ambulatory and ventilation-assisted: £23,600<br>- Non-ambulatory with scoliosis: £25,058 - £46,043<br>- Non-ambulatory and ventilation-assisted with scoliosis: £25,058 - £46,043	–

Publication	Patient population	Sample size	Currency (year)	Direct costs (medical and non-medical)	Indirect costs	Total costs and cost drivers	Resource use
				- Surgery costs: £20,986<br>- Surgery follow-up costs: £1,458<br>Non-ambulatory and ventilation-assisted with scoliosis: £4,012:<br>- Surgery costs: £20,986<br>- Surgery follow-up costs: £1,458<br><br>Base case costs included in sensitivity analysis:<br>- Ambulatory: £1,633<br>- Non-ambulatory: £4,012<br>- Non-ambulatory and ventilation-assisted: £4,012<br>- Non-ambulatory with scoliosis: £5,470<br>- Non-ambulatory and ventilation-assisted with scoliosis: £5,470<br><br>Discounted costs by health state - ataluren:<br>- Ambulatory: £4,984,263<br>- Non-ambulatory: £9,774<br>- Non-ambulatory and ventilation-assisted: £521<br>- Non-ambulatory with scoliosis: £37,961<br>- Non-ambulatory and ventilation-assisted with scoliosis: £60,021<br><br>Discounted costs by health state - BSC:<br>- Ambulatory: £29,752<br>- Non-ambulatory: £34,657<br>- Non-ambulatory and ventilation-assisted: £520<br>- Non-ambulatory with scoliosis: £96,964<br>- Non-ambulatory and ventilation-assisted with scoliosis: £73,314<br><br>Costs by category of cost per patient - ataluren:<br>- Technology cost: £4,919,878			

Publication	Patient population	Sample size	Currency (year)	Direct costs (medical and non-medical)	Indirect costs	Total costs and cost drivers	Resource use
				- Health state cost: £172,662<br><br>Costs by category of cost per patient - BSC:<br>- Technology cost: £0<br>- Health state cost: £235,207			
NICE submission, 2022 (HST22)[59]<br>Cost-effectiveness analysis	Ambulatory patients with DMD	–	GBP (2021)	Ataluren list price by sachet size:<br>- 125 mg: £84.40<br>- 250 mg: £168.80<br>- 1,000 mg: £675.20<br><br>Costs of ataluren treatment per patient per 3-month cycle:<br>- Ambulatory patient, based on average weight 35.5kg (assuming 95% compliance): £80,536<br>- Non-ambulatory patient, based on average weight 39.5kg (assuming 85% compliance): £78,609<br>- Administration cost: £0<br>- Training cost: £0<br>- Other costs (monitoring, tests etc): £0	–	Total ataluren treatment cost per patient per 3-month cycle:<br>- Ambulatory patient: £80,536<br>- Non-ambulatory patient: £78,609	–
Posner, 2023[60]<br>Retrospective cohort study	DMD patients aged ≤40 years	561	USD (NR)	–	–	Annual all-cause healthcare costs in USD by patients' race, mean (SD):<br><br>Baseline:<br>- White (n=360): 84,292<br>- Black (n=50): 40,448<br>- Hispanic (n=33): 62,488<br>- Unknown/other (n=118): 36,121<br><br>12-month follow up:<br>- White: 108,895 (346,934)<br>- Black: 59,501 (85,758)<br>- Hispanic: 61,199 (67,021)<br>- Unknown/other: 65,247 (119,733)	Annual HCRU during follow up by patients' race, n (%):<br><br>Any DMD-related device or procedure:<br>- White: 263 (73.1)<br>- Black: 35 (70.0)<br>- Hispanic: 26 (78.8)<br>- Unknown/other: 90 (76.3)<br><br>Ambulation devices or procedure:<br>- White: 204 (56.7)<br>- Black: 29 (58.0)<br>- Hispanic: 22 (66.7)<br>- Unknown/other: 70 (59.3)<br><br>Ventilation devices or

Publication	Patient population	Sample size	Currency (year)	Direct costs (medical and non-medical)	Indirect costs	Total costs and cost drivers	Resource use
							procedures:<br>- White: 141 (39.2)<br>- Black: 18 (36.0)<br>- Hispanic: 18 (54.6)<br>- Unknown/other: 50 (42.4)<br><br>Top five devices/procedures:<br><br>Wheelchair and accessories:<br>- White: 134 (37.2)<br>- Black: 17 (34.0)<br>- Hispanic: 15 (45.5)<br>- Unknown/other: 48 (40.7)<br><br>Power wheelchair and accessories:<br>- White: 119 (33.1)<br>- Black: 16 (32.0)<br>- Hispanic: 14 (42.4)<br>- Unknown/other: 47 (39.8)<br><br>Other ventilation devices:<br>- White: 87 (24.2)<br>- Black: 14 (28.0)<br>- Hispanic: 12 (36.4)<br>- Unknown/other: 38 (32.2)<br><br>Tracheostomy:<br>- White: 76 (21.1)<br>- Black: 6 (12.0)<br>- Hispanic: 11 (33.3)<br>- Unknown/other: 26 (22.0)<br><br>Ankle/foot orthoses:<br>- White: 59 (16.4)<br>- Black: 11 (22.0)<br>- Hispanic: 8 (24.2)<br>- Unknown/other: 21 (17.8)
Reynolds, 2023[61]<br>Retrospective cohort study	Patients of any age with DMD and ≥2 medical or	163	USD (2019)	–	–	Total out-of-pocket costs in USD per 30-day supply, median (IQR):<br>- Deflazacort (2017 vs	Insurance plan type, n (%) patients:<br>- Exclusive provider organisation: 11 (7)

Publication	Patient population	Sample size	Currency (year)	Direct costs (medical and non-medical)	Indirect costs	Total costs and cost drivers	Resource use
	pharmaceutical insurance claims					2019): 52 (17-72) vs 63 (39-139)<br>- Prednisone (2019): 5 (2-9)<br><br>Standardised total costs per 30-day supply, median (IQR):<br>- Deflazacort: 5,705 (5,134-7,324)<br>- Prednisone: 8 (5-11)	- Health maintenance organisation: 28 (18)<br>- Indemnity: 0 (0)<br>- Other: 19 (12)<br>- Point of service: 89 (55)<br>- Preferred provider organisation: 14 (9)<br>- Multiple plans: 2 (1)<br>- Missing/unknown: 0 (0)<br><br>Insurance plan type, n (%) patients:<br>- Commercial: 126 (77)<br>- Medicare: 37 (23)<br>- Missing/unknown: 0 (0)<br><br>Highly deductible plan status, n (%) patients:<br>- Health retirement account: 3 (3)<br>- Health savings account: 25 (22)<br>- No health retirement account or health savings account: 86 (74)<br>- Multiple plans: 2 (2)<br>- Missing/unknown: 47<br><br>Utilisation in 2019, n (%) patients:<br>- Deflazacort: 17 (20)<br>- Eteplirsen: 0 (0)<br>- Prednisone: 69 (80)
Rodriguez, 2022[62]<br>Cross-sectional	Informal caregivers (parents) of children with DMD	74 (40 from Spain)	EUR (NR)	–	Average monthly costs per caregiver, EUR:<br>Expenses associated with health professionals for the child:	–	Caregivers' occupational status (Spain only), n (%):<br>- Employee: 16 (40)<br>- Self-employed: 3 (7.5)<br>- Unpaid work: 2 (5)<br>- Unemployed (for health reasons): 1 (2.5)<br>- Unemployed (for other reasons): 6 (15)

Publication	Patient population	Sample size	Currency (year)	Direct costs (medical and non-medical)	Indirect costs	Total costs and cost drivers	Resource use
					- Spain: 1,384.10<br>- Mexico: 151.58<br>Expenses associated with assistive technology:<br>- Spain: 44,734.12<br>- Mexico: 3,487.66<br>Expenses associated with health professionals for the caregiver:<br>- Spain: 213.30<br>- Mexico: 75.98<br>Subsidies for expenses associated with health professionals for the child:<br>- Spain: 161.36<br>- Mexico: 49.52<br>Subsidies for assistive technology:<br>- Spain: 2,884.75<br>- Mexico: 249.00<br>Subsidies for expenses associated with health professionals for the caregiver:<br>- Spain: 0.00<br>- Mexico: 22.05<br>Percentage of family income		- Retired: 1 (2.5)<br>- Housework: 5 (12.5)<br>- Student: 5 (12.5)<br>- Disabled: 1 (2.5)<br><br>Hours spent on care per patient, average (mid-range): 44.62 (40)<br><br>Percentage of annual income related to caregivers' health expenses (Spain only), mean (SD) %:<br>- Expenses associated with services provided by health professionals for the child: 4.61 (9.61)<br>- Expenses related to assistive technology: 8.68 (17.23)<br>- Expenses associated with services provided by health professionals for the caregiver: 0.73 (2.05)<br><br>HCRU, mid-range (%):<br>- Family income spent on health professionals for the child: 32.91<br>- Household income spent on assistive technology: 33.63<br>- Family income spent on health professionals for the caregiver: 34.49

Publication	Patient population	Sample size	Currency (year)	Direct costs (medical and non-medical)	Indirect costs	Total costs and cost drivers	Resource use
					spent on health professionals for the child:<br>- Spain: 0.04<br>- Mexico: 0.05<br>Percentage of household income spent on assistive technology:<br>- Spain: 0.08<br>- Mexico: 0.07<br>Percentage of family income spent on health professionals for the caregiver:<br>- Spain: 0.00<br>- Mexico: 0.03<br><br>Percentage of annual income related to caregivers' health expenses, mean (SD):<br>Expenses associated with services provided by health professionals for the child:<br>- Spain: 4.61 (9.61)<br>- Mexico: 5.89 (13.73)<br>Expenses related to assistive technology:		

Publication	Patient population	Sample size	Currency (year)	Direct costs (medical and non-medical)	Indirect costs	Total costs and cost drivers	Resource use
					- Spain: 8.68 (17.23)<br>- Mexico: 7.42 (18.02)<br>Expenses associated with services provided by health professionals for the caregiver:<br>- Spain: 0.73 (2.05)<br>- Mexico: 3.45 (11.20)		
Schreiber-Katz, 2014[63]<br>Cross-sectional	DMD patients aged ≥16 years and/or their caregivers (parents)	248	EUR (2013)	Annual cost in EUR per patient, mean (range)<br><br>Medical costs:<br>- Outpatient treatment: 457 (0-4,644)<br>- Inpatient treatment: 1,613 (0-131,454)<br>- Rehabilitation program (in-/outpatient): 1,130 (0-30,053)<br>- Drug treatment: 330 (0-5,530)<br>- Rehabilitation services (e.g. physiotherapy): 4,732 (0-31,974)<br>- Medical aids: 10,209 (0-103,977)<br>- Respiratory management: 875 (0-12,087)<br><br>Non-medical costs:<br>- Housing situation: 4,043 (0-238,800)<br>- Personal assistance for school and work attendance: 883 (0-28,800)<br>- Travel expenses: 1,102 (0-17,376)<br>- Advocate support costs: 27 (0-	Annual cost in EUR per patient/caregiver, mean (range):<br>- Caused by patients' absenteeism and reduced working time: 21,463 (0-43,740)<br>- Caused by caregivers' absenteeism and reduced working time: 7,220 (0-324,000)	Mean annual total costs in EUR per patient:<br><br>Direct costs:<br>- Medical costs: 19,346<br>- Non-medical costs: 30,884<br>- Total: 50,230<br><br>Indirect costs: 28,683<br><br>Direct and indirect costs: 78,913	Employment status, patients; caregivers (%):<br>Non-working: 49; 8<br>Currently working:<br>- Self-employed 2; 7<br>- Employed 37; 56<br>Quit working life: 12; 29<br>- In clinical severity stage I: NA; 17<br>- In stage II: 0; 52<br>- In stage III: 25; 15<br>- In stage IV: 50; 13<br>- In stage V: 25; 3<br>Reduced working time: 6; 38<br>- In stage I: 10; 22<br>- In stage II: 11; 55<br>- In stage III: 11; 13<br>- In stage IV: 67; 19<br>- In stage V: 11; 3<br><br>HCRU, % patients:<br>- Outpatient medical consultation: 77<br>- Consultation of specialised outpatient clinics: 24<br>- Psychological assistance: 10

Publication	Patient population	Sample size	Currency (year)	Direct costs (medical and non-medical)	Indirect costs	Total costs and cost drivers	Resource use
				8,333)<br>- Investments in house adaptions: 3,059 (0-93,636)<br>- Investments in automobile adaptions: 408 (0-16,697)<br>- Other expenditures (e.g. artificial nutrition, alternative therapies): 83 (0-12,000)<br>- Informal care costs: 21,279 (0-223,380)			- Nutrition counselling: 5<br>- Sleep laboratory investigation: 9<br>- In-patient treatment: 13<br>- In-/outpatient rehabilitation programs: 8<br>- Drug treatment: 62<br>- Rehabilitation services: 92<br>- Medial aids: 85<br>- Respiratory management: 3<br><br>Subjectively care-related HCRU of caregivers, % caregivers:<br>- Care-related medical problems: 57<br>- Medical treatment needed: 74<br>- Medical rehabilitation needed: 12
SMC submission, 2023[64]<br>Cost-utility analysis	Patients aged ≥2 years old with DMD	–	GBP (2021)	Ataluren cost per year (calculated using weight range 12-70kg): £123,224 - £739,344<br>Ataluren cost per three-monthly cycle (based on an average weight of 35kg): £84,774	–	–	–
Soelaeman, 2021[65]<br>Cross-sectional	Primary, female caregivers of male patients <18 years old with DMD	86	USD (2020)	–	Total annual productivity loss in USD per caregiver, mean (range):<br>- Patients without LOA: -41 (-3,593, 3511)<br>- Patients with 0-3 years of LOA: -13,828 (-20,124, -7533)<br>- Patients with 0-3 years of LOA: -23,995 (-29,058, -	–	Labor market outcomes (caregivers):<br><br>Worked in the past year, n (%): 63 (67.7)<br>- Patients without LOA: 39 (75.0)<br>- Patients with 0-3 years of LOA: 10 (58.8)<br>- Patients with ≥4 years of LOA: 14 (58.3)<br><br>Weeks worked in the past year, mean (SE):<br>- Patients without LOA: 47.3 (1.6)

Publication	Patient population	Sample size	Currency (year)	Direct costs (medical and non-medical)	Indirect costs	Total costs and cost drivers	Resource use
					18,931)		- Patients with 0-3 years of LOA: 48.7 (1.9)<br>- Patients with ≥4 years of LOA: 40.6 (3.8)<br><br>Hours worked per week, mean (SE):<br>- Patients without LOA: 33.2 (2.4)<br>- Patients with 0-3 years of LOA: 28.9 (4.0)<br>- Patients with ≥4 years of LOA: 26.8 (3.7)<br><br>Loss in work hours/week, mean (95% CI):<br>- Patients without LOA: -1.0 (3.2, 1.3)<br>- Patients with 0-3 years of LOA: -10.7 (-14.8, -6.7)<br>- Patients with ≥4 years of LOA: -15.0 (-18.5, -11.5)<br><br>Loss in annualised work hours, mean (95% CI):<br>- Patients without LOA: -1.5 (-111.1, 108)<br>- Patients with 0-3 years of LOA: -459.0 (-658.2, -259.8)<br>- Patients with ≥4 years of LOA: -809.4 (-977, -641.8)
Thayer, 2017[66]<br>Retrospective cohort study	Male patients with DMD aged <30 years old with ≥1 medical insurance claim	75	USD (2010)	Annual healthcare cost in USD per patient, mean (SD)<br>- Ambulatory: 4,838 (5,105)<br>- Inpatient: 11,274 (33,859)<br>- Emergency room: 517 (1,706)<br>- Other (includes items such as durable medical equipment and ambulance): 5,901 (9,695)<br>- Medical cost: 22,530 (39,041)<br>- Pharmacy cost: 1,487 (2,861)	–	Total annual healthcare costs in USD per patient at baseline, mean (SD): 24,017 (39,369)<br><br>Mean estimated annual total healthcare costs: 23,005<br>- Patients aged 0-13 years (n=451): 21,553<br>- Patients aged 14-29 years (n=374): 40,132	Annual HCRU per patient, mean (SD):<br>- Office visit: 11.92 (16.92)<br>- Outpatient visit: 6.42 (6.39)<br>- Inpatient admission: 0.23 (0.47)<br>- Emergency room visit: 0.68 (1.22)<br>- Unique medications: 2.95 (2.47)<br>- Medication dispensing per

Publication	Patient population	Sample size	Currency (year)	Direct costs (medical and non-medical)	Indirect costs	Total costs and cost drivers	Resource use
							year (prescription fills): 16.31 (18.81)
Villa, 2022[67]<br>Cross-sectional	Paediatric cardiologists directly involved with DMD care	31	–	–	–	–	How often cardiologists see DMD patients, n (%):<br>- At the time of their diagnosis: 28 (90)<br>For patients <10 years old:<br>- Every 2 years: 10 (32)<br>- Every year: 16 (52)<br>- Every 6 months: 5 (16)<br>For patients >10 years old:<br>- Every year: 11 (35)<br>- Every 6 months: 20 (65)<br><br>Use of cardiac MRI for DMD patients, n (%) cardiologists:<br>-At clinical evaluation: 27 (87)<br>- By 10 years old: 17 (55)<br>- By 13 years old: 10 (32)<br>With sedation: 2 (6)<br>Serially: 13 (42)<br>- Yearly: 6 (19)<br>- Every 2 years: 7 (23)<br><br>Use electrophysiologic monitoring for DMD patients, n (%) cardiologists:<br>- Holter monitoring: 26 (84)<br><br>Use of ACEi/ARB for DMD patients, n (%) cardiologists:<br>- Routine component of therapy: 31 (100)<br>- By 10 years old: 21 (68)<br>- Using angiotensin receptor neprolysin inhibitor: 7 (23)<br><br>Use of beta-blockers for DMD patients, n (%) cardiologists:<br>- Routine component of therapy: 31 (100)

Publication	Patient population	Sample size	Currency (year)	Direct costs (medical and non-medical)	Indirect costs	Total costs and cost drivers	Resource use
							Use of aldosterone antagonists for DMD patients, n (%) cardiologists:<br>- Routine component of therapy: 28 (90)<br>- Prophylactically by 10 years old: 3 (10)<br><br>Used implantable cardioverter defibrillator for DMD, n (%) cardiologists: 17 (55)<br>- Never used for primary prevention: 11 (35)<br><br>Providers who would consider implanting a ventricular assist device in DMD according to device intent, n (%):<br>- Destination therapy: 23 (74)<br>- Bridge to transplant: 3 (10)<br>- Would not consider ventricular assist device: 5 (16)<br><br>Used left ventricular assist device for DMD, n (%) cardiologists: 7 (23)<br>Providers who would consider listing a patient with DMD for heart transplant, n (%): 16 (52)
Vry, 2016[68]<br>Cross-sectional	Patients of any age with DMD	1,062	–	–	–	–	Indicators of patient care for the whole cohort and different age groups in the cohort:<br><br>Patients receiving physiotherapy:<br>- All patients (n=1,015):

Publication	Patient population	Sample size	Currency (year)	Direct costs (medical and non-medical)	Indirect costs	Total costs and cost drivers	Resource use
							72.8%<br>- Young children (<6 years of age; n=819): 78.1%<br>- Adults (≥18 years of age; n=196): 67.9%<br><br>Patients (≥10 years of age) receiving echocardiography according to guidelines (once yearly):<br>- All patients (n=635): 77.9%<br>- Young children (n=439): 82.2%<br>- Adults (n=196): 67.4%<br><br>Patients who reported unplanned hospital admissions within past 2 years:<br>- All patients (n=1,062): 13.6%<br>- Young children (n=861): 8.9%<br>- Adults (n=201): 25.4%<br><br>Non-ambulatory patients reporting current steroid use:<br>- All patients (n=552): 21.0%<br>- Germany: 21.6%<br>- UK: 35.7%<br><br>Patients ≥9 years of age reporting current or past steroid use:<br>- All patients (n=704): 65.2%<br>- Germany: 72.8%<br>- UK: 83.6%<br><br>Patients visiting a neuromuscular center at least annually:<br>- All patients (n=1,027): 81.9%

Publication	Patient population	Sample size	Currency (year)	Direct costs (medical and non-medical)	Indirect costs	Total costs and cost drivers	Resource use
							- Germany: 88.5%<br>- UK: 92.1%<br><br>Patients receiving physiotherapy:<br>- All patients (n=1,015): 76.2%<br>- Germany: 92.0%<br>- UK: 48.4%<br><br>Sufficient instructions to perform exercises in patients without current physiotherapy:<br>- All patients (n=238): 53.3%<br>- Germany: 21.9%<br>- UK: 70.6%<br><br>Patients (≥ 10 years of age) receiving echocardiography at least annually:<br>- All patients (n=633): 77.9%<br>- Germany: 90.7%<br>- UK: 81.0%<br><br>Ambulatory patients (≥6 years of age), receiving lung function testing according to international care recommendations:<br>- All patients (n=336): 62.8%<br>- Germany: 74.2%<br>- UK: 66.3%<br><br>Non-ambulatory patients (≥6 years of age), receiving lung function testing according to international care recommendations:<br>- All patients (n=547): 30.5%<br>- Germany: 44.1%<br>- UK: 45.5%

Publication	Patient population	Sample size	Currency (year)	Direct costs (medical and non-medical)	Indirect costs	Total costs and cost drivers	Resource use
							Non-ambulatory patients reporting spinal inspection:<br>- All patients (n=540): 20.2%<br>- Germany: 32.2%<br>- UK: 26.0%<br><br>Adherence to guidelines for cardiac and pulmonary care:<br><br>Lung function testing in ambulatory patients ≥6 years (n=331):<br>- Check-ups according to recommendation (annually): 63.7%<br>- Less frequent check-ups: 12.9%<br>- No check-ups: 23.3%<br><br>Lung function testing in non-ambulatory patients ≥6 years (n=546):<br>- Check-ups according to recommendation (bi-annually): 30.6%<br>- Less frequent check-ups: 59.3%<br>- No check-ups: 10.1%<br><br>Echocardiography in patients ≥10 years (n=635):<br>- Check-ups according to recommendation (at least once yearly): 77.9%<br>- Less frequent check-ups: 17.7%<br>- No check-ups: 4.4%<br><br>Care indictors in patients regularly and irregularly seeking care at a neuromuscular center:

Publication	Patient population	Sample size	Currency (year)	Direct costs (medical and non-medical)	Indirect costs	Total costs and cost drivers	Resource use
							No regular (at least once yearly) cardiac checkups:<br>- Regular visitors (at least once yearly; n=841): 21.0%<br>- Irregular visitors (less than once yearly; n=186): 52.3%<br><br>No regular (at least once yearly) pulmonary check-ups:<br>- Regular visitors (n=841): 30.9%<br>- Irregular visitors (n=186): 71.1%<br><br>Patients ≥9 years of age reporting current or past corticosteroid use:<br>- Regular visitors (n=841): 73.0%<br>- Irregular visitors (n=186): 37.2%<br><br>Patients who reported unplanned admissions to hospital:<br>- Regular visitors (n=841): 13.8%<br>- Irregular visitors (n=186): 15%<br><br>Mean (SD) duration of hospital stay for the unplanned admissions:<br>- Regular visitors (n=841): 9.3 (3.7)<br>- Irregular visitors (n=186): 23.0 (11.8)<br><br>Reasons for patients never seeking at a neuromuscular center, n:<br>- Centers were too far away: 49/109

Publication	Patient population	Sample size	Currency (year)	Direct costs (medical and non-medical)	Indirect costs	Total costs and cost drivers	Resource use
							- Not aware centers existed: 19/109<br>- Not need a neuromuscular specialist: 8/109
White, 2023[69]<br>Retrospective cohort study	Patients with DMD receiving SoC corticosteroids/ glucocorticoids for ≥12 months or who discontinued use of corticosteroids/ glucocorticoids within the past 12 months	103	–	–	–	–	Glucocorticoid experience, n (%):<br>Currently receiving glucocorticoids: 100 (97)<br>- Prednisone/prednisolone: 55 (53)<br>- Deflazacort: 45 (44)<br>Discontinued glucocorticoid in the past 12 months: 3 (3)<br><br>Treatment history of current prednisone users, n (%):<br>- Previously treated with deflazacort: 8 (15)<br>- Glucocorticoid naïve: 47 (85)<br><br>Treatment history of current deflazacort users, n (%):<br>- Previously treated with prednisone: 20 (44)<br>- Glucocorticoid naïve: 25 (56)<br><br>Treatment categories of all currently managed patients in the past 12 months, %:<br>- Currently taking glucocorticoids: 55<br>- Previously taken glucocorticoids: 28<br>- Glucocorticoid naïve: 17<br><br>Physicians who did not use glucocorticoids because of the cost: 7%<br>Patients/caregivers who refused glucocorticoids

Publication	Patient population	Sample size	Currency (year)	Direct costs (medical and non-medical)	Indirect costs	Total costs and cost drivers	Resource use
							because of the cost: 33%<br><br>Prednisone dosing, n (%):<br>- Indicated dose: 23 (30)<br>- Higher than indicated: 29 (37)<br>- Lower than indicated: 26 (33)<br><br>Deflazacort dosing, n (%):<br>- Indicated dose: 20 (37)<br>- Higher than indicated: 9 (17)<br>- Lower than indicated: 25 (46)<br><br>Surgery post-diagnosis, %:<br>- Yes: 10<br>- No: 90<br><br>Outpatient/lab visit over the past 12 months, %:<br>- Doctors visits for routine appointments: 90<br>- Lab visits for tests: 77<br>- Doctors visits for urgent care: 28<br>- Visits for medication administration: 24<br><br>Average outpatient/lab visits per patient over the past 12 months:<br>- Doctors visits for routine appointments: 4.6<br>- Lab visits for tests: 1.9<br>- Doctors visits for urgent care: 2.6<br>- Visits for medication administration: 4.4<br><br>Inpatient/ER visit over the past 12 months, %:

Publication	Patient population	Sample size	Currency (year)	Direct costs (medical and non-medical)	Indirect costs	Total costs and cost drivers	Resource use
							- ER (not including inpatient admission): 21<br>- Inpatient Hospitalisation (not including ER): 12
Bonarrigo, 2023[70]<br>Cross-sectional	Male patients with DMD aged ≥10 years old, or their parent /caregiver	147 (15 patients and 132 parents/ caregivers)	–	–	–	–	Insurance Type, n (%):<br>Total:<br>- Private: 102 (69.4)<br>- Public: 40 (27.2)<br>DMD patients:<br>- Private: 6 (40.0)<br>- Public: 9 (60.0)<br>Parents/caregivers:<br>- Private: 96 (72.7)<br>- Public: 31 (23.5)<br><br>Familiar with using standers for individuals with DMD, n (%): 120 (81.6)<br>- 108 parents/caregivers and 12 individuals with DMD<br><br>Currently using a stander for DMD, n (%): 42 (28.6)<br>- 38 parent/caregivers and 4 individuals with DMD<br>- Power standing feature in a wheelchair (including Permobil F5 and Permobil C400): 13<br>- Sit-to-stand stander: 27<br>- Type of stander not reported: 2<br><br>Respondents' methods of obtaining standers, n (%):<br>Total (n=42):<br>Funding sources:<br>- Insurance: 27 (64.3)<br>- Out-of-pocket: 7 (16.7)<br>- School: 5 (11.9)<br>- Loaner/Donor: 7 (16.7)<br>- Other: 1 (2.4)

Publication	Patient population	Sample size	Currency (year)	Direct costs (medical and non-medical)	Indirect costs	Total costs and cost drivers	Resource use
							- Multiple: 6 (14.3)<br>Third parties who helped user to obtain stander:<br>- Physical Therapist: 31 (73.8)<br>- Occupational Therapist: 3 (7.2)<br>- Medical equipment company: 19 (45.2)<br>- Other: 5 (11.9)<br>- Medical equipment company without therapist involvement: 6 (14.3)<br>- Medical equipment company with therapist involvement: 13 (30.95)<br><br>Power stander in wheelchair: (n=13):<br>Funding sources:<br>- Insurance: 11 (84.6)<br>- Out-of-pocket: 2 (15.4)<br>- School: 1 (7.7)<br>- Loaner/Donor: 1 (7.7)<br>- Other: 0 (0.0)<br>- Multiple: 2 (15.4)<br>Third parties who helped user to obtain stander:<br>- Physical Therapist: 11 (84.6)<br>- Occupational Therapist: 1 (7.7)<br>- Medical equipment company: 9 (69.2)<br>- Other: 1 (7.7)<br>- Medical equipment company without therapist involvement: 2 (15.4)<br>- Medical equipment company with therapist involvement: 7 (53.9)<br><br>Sit-to-stand stander (n=27):

Publication	Patient population	Sample size	Currency (year)	Direct costs (medical and non-medical)	Indirect costs	Total costs and cost drivers	Resource use
							Funding sources:<br>- Insurance: 15 (55.6)<br>- Out-of-pocket: 4 (14.8)<br>- School: 4 (14.8)<br>- Loaner/Donor: 6 (22.2)<br>- Other: 1 (3.7)<br>- Multiple: 4 (14.8)<br>Third parties who helped user to obtain stander:<br>- Physical Therapist: 18 (66.7)<br>- Occupational Therapist: 2 (7.4)<br>- Medical equipment company: 8 (29.6)<br>- Other: 4 (14.8)<br>- Medical equipment company without therapist involvement: 4 (14.8)<br>- Medical equipment company with therapist involvement: 4 (14.8)<br><br>Frequency of stander use, mean (SD) [range] days/week: 4.5 (1.7) [1-7]<br>Sit-to-stand, n:<br>- <5 days/week: 13<br>- ≥5 days/week: 13<br>Power stander in wheelchair, n:<br>- <5 days/week: 4<br>- ≥5 days/week: 9<br><br>Intensity (amount upright) of stander use, n:<br>Sit-to-stand:<br>- Fully upright: 13<br>- Nearly upright: 3<br>- Moderately upright: 5<br>- Mildly upright: 6<br>Power stander in wheelchair:<br>- Fully upright: 6

Publication	Patient population	Sample size	Currency (year)	Direct costs (medical and non-medical)	Indirect costs	Total costs and cost drivers	Resource use
							- Nearly upright: 4<br>- Moderately upright: 2<br>- Mildly upright: 1<br><br>Time in stander throughout the day, n:<br>Sit-to-stand:<br>- All at once: 22<br>- Multiple times: 5<br>Power stander in wheelchair:<br>- All at once: 8<br>- Multiple times: 5<br><br>Amount of stander use per day, mean (SD) [range] minutes: 44.8 (30.2) [10-180]<br>Sit-to-stand:<br>- ≥60 minutes: 10<br>- <60 minutes: 17<br>Power stander in wheelchair:<br>- ≥60 minutes: 5<br>- <60 minutes: 8<br><br>Location of stander use, n:<br>At home:<br>- Total: 38<br>- Sit-to-stand: 24<br>- Power stander in wheelchair: 12<br>- Other stander type: 2<br>At school: 9<br>During therapy appointments: 1<br>Other locations: 7<br>Multiple locations:<br>- Sit-to-stand: 2<br>- Power stander in wheelchair: 7
Broomfield, 2023[71]<br>Retrospective cohort study	Patients with DMD	1,121	–	–	–	–	Steroid users, n: 724<br>Underwent spinal surgery, n: 242<br>Ventilation users, n: 357

Publication	Patient population	Sample size	Currency (year)	Direct costs (medical and non-medical)	Indirect costs	Total costs and cost drivers	Resource use
							Events in all patients, n (%):<br>- First steroid use: 479 (46.7)<br>- Spinal surgery: 155 (15.0)<br>- Ventilation: 286 (27.1)<br><br>Events in patients under 18, n (%):<br>- First steroid use: 323 (63.2)<br>- Spinal surgery: 36 (7.1)<br>- Ventilation: 61 (11.9)<br><br>Events in patients over 18, n (%):<br>- First steroid use: 252 (41.3)<br>- Spinal surgery: 206 (33.8)<br>- Ventilation: 292 (47.9)
Castro, 2023[39]<br>Prospective cohort study	Male patients with DMD	207 (118 USA and 89 Spain)	–	–	–	–	Disease Stage key:<br>1 (early ambulatory)<br>2 (late ambulatory)<br>3 (transitional - cannot walk 10m, cannot stand from supine but can remain standing)<br>4 (non-ambulatory - HTMF, no ventilator, unable to stand)<br>5 (non-ambulatory - no HTMF, no ventilator, Brooke >4)<br>6 (non-ambulatory - HTMF, nighttime ventilation, Brooke ≤4)<br>7 (non-ambulatory - no HTMF, nighttime ventilation, Brooke >4)<br>8 (non-ambulatory - full ventilation, FVC <30%)<br><br>Mean HCRU in the last 12 months

Publication	Patient population	Sample size	Currency (year)	Direct costs (medical and non-medical)	Indirect costs	Total costs and cost drivers	Resource use
							Mean (SD) scheduled physician consultations<br>- Stage 1: 2.50 (1.97)<br>- Stage 2: 3.45 (2.78)<br>- Stage 3: 3.70 (1.52)<br>- Stage 4: 3.03 (2.10)<br>- Stage 5: 4.93 (3.36)<br>- Stage 6: 4.39 (3.13)<br>- Stage 7: 4.72 (3.94)<br>- Stage 8: 5.00 (3.04)<br><br>Mean (SD) scheduled nurse specialist consultations:<br>- Stage 1: 1.35 (1.23)<br>- Stage 2: 1.48 (1.78)<br>- Stage 3: 2.53 (2.70)<br>- Stage 4: 1.59 (1.72)<br>- Stage 5: 3.29 (3.15)<br>- Stage 6: 2.44 (1.89)<br>- Stage 7: 3.28 (5.11)<br>- Stage 8: 4.00 (3.43)<br><br>Mean (SD) Hospitalisations:<br>- Stage 1: 0.21 (0.59)<br>- Stage 2: 0.57 (1.13)<br>- Stage 3: 0.58 (0.68)<br>- Stage 4: 0.47 (0.72)<br>- Stage 5: 0.93 (0.92)<br>- Stage 6: 1.06 (1.00)<br>- Stage 7: 1.22 (1.44)<br>- Stage 8: 1.78 (1.92)<br><br>Mean (SD) surgeries:<br>- Stage 1: 0.03 (0.17)<br>- Stage 2: 0.05 (0.22)<br>- Stage 3: 0.28 (0.60)<br>- Stage 4: 0.16 (0.37)<br>- Stage 5: 0.57 (0.65)<br>- Stage 6: 0.33 (0.49)<br>- Stage 7: 0.22 (0.55)<br>- Stage 8: 0.56 (0.73)<br><br>Mean (SD) tests:

Publication	Patient population	Sample size	Currency (year)	Direct costs (medical and non-medical)	Indirect costs	Total costs and cost drivers	Resource use
							- Stage 1: 4.06 (3.55)<br>- Stage 2: 2.74 (3.97)<br>- Stage 3: 4.98 (6.15)<br>- Stage 4: 3.84 (4.48)<br>- Stage 5: 5.14 (5.16)<br>- Stage 6: 7.00 (8.00)<br>- Stage 7: 5.28 (4.76)<br>- Stage 8: 6.11 (5.23)<br><br>Mean (SD) treatments:<br>- Stage 1: 1.97 (1.85)<br>- Stage 2: 2.24 (2.21)<br>- Stage 3: 2.6 (1.93)<br>- Stage 4: 3.06 (2.34)<br>- Stage 5: 3.57 (1.87)<br>- Stage 6: 2.89 (2.27)<br>- Stage 7: 3.61 (2.00)<br>- Stage 8: 3.00 (1.66)
Innis, 2023[72]<br>Partitioned survival model	Patients with DMD	–	USD (NR)	–	Base case results (lifetime horizon):<br><br>Patients treated with SoC (corticosteroids and medical management):<br>Income (undiscounted model): $63,096<br>- Loss vs US male population: $7,378,226 (99.2%)<br>Income (discounted model): $35,085<br>- Loss vs US male population: $2,113,808 (98.4%)<br><br>Patients treated	–	Average working years over lifetime:<br>Patients treated with SoC: 0.88<br>- Loss vs US male population: 35.85 (97.6%)<br>Patients treated with Delandistrogene Moxeparvovec + SoC:11.25<br>- Loss vs US male population: 25.48 (69.4%)<br>- Gain vs SoC: 10.37

Publication	Patient population	Sample size	Currency (year)	Direct costs (medical and non-medical)	Indirect costs	Total costs and cost drivers	Resource use
					with Delandistrogene Moxeparvovec + SoC:<br>Income (undiscounted): $1,689,248<br>- Loss vs US male population: $5,752,074 (77.3%)<br>- Gain vs SoC: $1,626,152<br>Income (discounted): $601,610<br>- Loss vs US male population: $1,547,283 (72.0%)<br>- Gain vs SoC: $566,525		
Strober, 2023a[73]<br>Cross-sectional	Male patients with DMD aged ≤18 years	425	–	–	–	–	HCRU in the past 12 months<br><br>Hospitalisations due to DMD, mean (SD):<br>- Total: 0.2 (0.54)<br>- Patients 0-12 years old (n=264): 0.2 (0.50)<br>- Patients aged 13-18 years old (n=161): 0.2 (0.59)<br><br>Hospitalised due to DMD, n (%):<br>- Total: 63 (14.8)<br>- Patients 0-12 years old: 34 (12.9)<br>- Patients aged 13-18 years old: 29 (18.0)<br><br>Hospitalisations involving

Publication	Patient population	Sample size	Currency (year)	Direct costs (medical and non-medical)	Indirect costs	Total costs and cost drivers	Resource use
							ICU stay, n (%):<br>- Total: 9 (14.3)<br>- Patients 0-12 years old: 3 (8.8)<br>- Patients aged 13-18 years old: 6 (20.7)<br><br>Number of patients who had surgery for DMD-related issue, n (%):<br>- Total: 11 (17.5)<br>- Patients 0-12 years old: 2 (5.9)<br>- Patients aged 13-18 years old: 9 (31.0)<br><br>Number of different physicians seen, mean (SD):<br>- Total: 4.0<br>- Patients 0-12 years old: 3.7 (2.0)<br>- Patients aged 13-18 years old: 4.5 (2.8)<br><br>Number of consultations with healthcare professionals, mean (SD):<br>- Total: 21.3<br>- Patients 0-12 years old: 16.9 (31.0)<br>- Patients aged 13-18 years old: 28.5 (47.7)<br><br>Number of tests conducted to monitor condition, mean (SD):<br>- Total: 11.3<br>- Patients 0-12 years old: 9.7 (12.6)<br>- Patients aged 13-18 years old: 13.7 (13.0)<br><br>Patients using mobility

Publication	Patient population	Sample size	Currency (year)	Direct costs (medical and non-medical)	Indirect costs	Total costs and cost drivers	Resource use
							aids/supports, %:<br>Any:<br>- Total: 68.4%<br>- Patients 0-12 years old: 61.2%<br>- Patients aged 13-18 years old: 84.5%<br>Orthoses:<br>- Patients 0-12 years old: 32.2%<br>- Patients aged 13-18 years old: 42.4%<br>Electric wheelchair:<br>- Patients 0-12 years old: 9.8%<br>- Patients aged 13-18 years old: 46.6%<br>Manual wheelchair:<br>- Patients 0-12 years old: 12.9%<br>- Patients aged 13-18 years old: 16.8%<br>Stroller/pram:<br>- Patients 0-12 years old: 14.4%<br>- Patients aged 13-18 years old: 0%<br>Electric scooter:<br>- Patients 0-12 years old: 1.9%<br>- Patients aged 13-18 years old: 5.0%<br><br>Patients with home modifications:<br>- Total: 63.5%<br>- Patients 0-12 years old: 52.2%<br>- Patients aged 13-18 years old: 83.2%<br><br>Physician-reported home modifications, % patients:

Publication	Patient population	Sample size	Currency (year)	Direct costs (medical and non-medical)	Indirect costs	Total costs and cost drivers	Resource use
							Adapted bathroom:<br>- Patients 0-12 years old: 47.9%<br>- Patients aged 13-18 years old: 62.4%<br>Adapted bedroom:<br>- Patients 0-12 years old: 36.1%<br>- Patients aged 13-18 years old: 51.4%<br>Fitted grab bars/railings:<br>- Patients 0-12 years old: 37.8%<br>- Patients aged 13-18 years old: 44.0%<br>Installation of ramps:<br>- Patients 0-12 years old: 20.2%<br>- Patients aged 13-18 years old: 29.4%<br>Fitted a stair lift:<br>- Patients 0-12 years old: 16.8%<br>- Patients aged 13-18 years old: 22.9%

Strober, 2023b[74]<br><br>Cross-sectional	Caregivers of patients with DMD	77 (48 Europe, 26 Japan, 3 USA)	–	–	–	–	Hours of DMD care per week, mean (SD):<br>- Total: 43.4 (32.2)<br>- Europe: 41.6 (27.0)<br>- USA: 33.3 (32.2)<br><br>Proportion of caregivers with a change to their employment due to caring for the patient with DMD:<br>- Total: 35.3%<br>- Europe: 52.4%<br>- USA: 50.0%<br><br>Proportion of caregivers reducing working hours:<br>- Total: 23.5%<br>- Europe: 33.3%<br>- USA: 50.0%<br><br>Proportion of patients with professional (paid) caregivers:<br>- Total: 37.3%<br>- Europe: 45.7%<br>- USA: 66.7%<br><br>Work Productivity and Activity Impairment of caregivers<br>Overall work impairment:<br>- Total: 44.1%<br>- Europe: 47.2%<br>- USA: 11.8%<br><br>Absenteeism:<br>- Total: 6.5%<br>- Europe: 7.0%<br>- USA: 2.3%<br><br>Presenteeism:<br>- Total: 38.8%<br>- Europe: 41.4%<br>- USA: 10.0%

Publication	Patient population	Sample size	Currency (year)	Direct costs (medical and non-medical)	Indirect costs	Total costs and cost drivers	Resource use
							Activity impairment:<br>- Total: 54.1%<br>- Europe: 47.6%<br>- USA: 40.0%

Table A10 Results of the quality assessment of HCRU & cost studies using the checklist published by Molinier et al. (2008) [87] (n=29 full-text publications)

Publication	1. Was a clear definition of the illness given?	2. Were epidemiologi-cal sources carefully described?	3. Were direct/indirect costs sufficiently disaggregated?	4. Were activity data sources carefully described?	5. Were activity data appropriately assessed?	6. Were the sources of all cost values analytically described?	7. Were unit costs appropriately valued?	8. Were the methods adopted carefully explained?	9. Were the major assumptions tested in a sensitivity analysis?	10. Was the presentation of study results consistent with the methodology of the study?
**Bach, 2015[40]**	Yes	N/A	No	Yes	Yes	Yes	Yes	Yes	N/A	Yes
**Bonarrigo, 2023[70]**	Yes	N/A	N/A	Yes	Yes	N/A	N/A	Yes	N/A	Yes
**Broomfield, 2023[71]**	Yes	Yes	N/A	N/A	N/A	N/A	N/A	Yes	N/A	Yes
**Cavazza, 2016[16]**	Yes	N/A	Yes	Yes	Yes	Yes	Yes	Yes	N/A	Yes
**Conway, 2022[41]**	Yes	N/A	No	Yes	Partial yes	Yes	Yes	Yes	No	Yes
**Donaldson, 2021[42]**	Yes	N/A	N/A	Yes	Yes	N/A	N/A	Yes	N/A	Yes
**Flores, 2020[43]**	Yes	Yes	No	Yes	Yes	Yes	No	Yes	No	Yes
**Hurtvitz, 2021[44]**	Yes	Yes	N/A	Yes	Yes	N/A	N/A	Yes	N/A	Yes
**Iff, 2022[45]**	Yes	N/A	No	Yes	Yes	Yes	N/A	Yes	N/A	Yes
**Iff, 2023[46]**	Yes	N/A	N/A	Yes	Yes	N/A	N/A	Yes	Yes	Yes
**Klimchak, 2021[49]**	Yes	N/A	No	Yes	Yes	Yes	No	Yes	Yes	Yes
**Klimchak, 2023 [50]**	Yes	Yes	No	Yes	Yes	Yes	Yes	Yes	Yes	Yes
**Landfelt, 2014[17]**	Yes	Partial yes	Partial yes	Yes	Yes	Yes	Yes	Yes	N/A	Yes
**Landfelt, 2015[52]**	Yes	N/A	N/A	Yes	Yes	N/A	N/A	Yes	N/A	Yes
**Landfeldt, 2017a[51]**	Yes	N/A	Yes	Partial yes	Partial yes	Yes	No	Yes	Yes	Yes

Publication	1. Was a clear definition of the illness given?	2. Were epidemiologi-cal sources carefully described?	3. Were direct/indirect costs sufficiently disaggregated?	4. Were activity data sources carefully described?	5. Were activity data appropriately assessed?	6. Were the sources of all cost values analytically described?	7. Were unit costs appropriately valued?	8. Were the methods adopted carefully explained?	9. Were the major assumptions tested in a sensitivity analysis?	10. Was the presentation of study results consistent with the methodology of the study?
Landfeldt, 2017b[53]	Yes	Yes	No	Yes	Yes	Yes	N/A	Partial yes	No	Yes
Lang, 2019[54]	Yes	N/A	No	Yes	Yes	Yes	Yes	Yes	N/A	Yes
Lin, 2019 (updated 2022)[55]	Yes	Yes	Yes	N/A	N/A	Yes	Yes	Yes	Yes	Yes
Magliano, 2014[56]	Yes	N/A	N/A	Yes	Yes	N/A	N/A	Yes	N/A	Yes
NICE submission, 2016[58]	Yes	Yes	Yes	N/A	N/A	Yes	Yes	Yes	Yes	Yes
NICE submission, 202 [59]	Yes	Yes	No	N/A	N/A	Yes	Yes	Yes	Yes	Partial yes
Reynolds, 2023[61]	Partial yes	N/A	No	N/A	N/A	Yes	N/A	Yes	N/A	Yes
Rodriguez, 2022[62]	Yes	N/A	Partial yes	Partial yes	Yes	Partial yes	Partial yes	Partial yes	N/A	Yes
Schreiber-Katz, 2014[63]	Yes	N/A	Yes	Yes	Yes	Yes	Yes	Yes	N/A	Yes
SMC submission, 2023[64]	Yes	Yes	No	N/A	N/A	Yes	No	Yes	Yes	Yes
Soelaeman, 2021[65]	Yes	N/A	Yes	Yes	Yes	Yes	N/A	Yes	N/A	Yes
Thayer, 2017[66]	Yes	N/A	No	Yes	Yes	Yes	Yes	Yes	N/A	Yes
Villa, 2022[67]	Yes	N/A	N/A	Yes	Yes	N/A	N/A	Yes	N/A	Yes

Publication	1. Was a clear definition of the illness given?	2. Were epidemiologi-cal sources carefully described?	3. Were direct/indirect costs sufficiently disaggregated?	4. Were activity data sources carefully described?	5. Were activity data appropriately assessed?	6. Were the sources of all cost values analytically described?	7. Were unit costs appropriately valued?	8. Were the methods adopted carefully explained?	9. Were the major assumptions tested in a sensitivity analysis?	10. Was the presentation of study results consistent with the methodology of the study?
**Vry, 2016[68]**	Yes	N/A	N/A	Yes	Yes	N/A	N/A	Partial yes	N/A	Yes

HCRU healthcare resource use, *N/A* not applicable, *NICE* National Institute for Health and Care Excellence, *SMC* Scottish Medicines Consortium.

3. Economic evaluations SLR

The objective of this SLR was to identify economic evaluations in patients with DMD.

3.1.1. Methods

Table A11 Electronic databases included in the economic evaluation SLR updates

Database	Platform	2023 SLR update		2024 SLR update	
		Span of search	Date searched	Span of search	Date searched
Embase	Ovid	8th July 2018 to 11th October 2023	12th October 2023	12th October 2023 to 9th January 2024	10th January 2024
MEDLINE Daily, In-Process & Other Non-indexed citations, and e-pub ahead-of-print		8th July 2018 to 11th October 2023	12th October 2023	12th October 2023 to 9th January 2024	10th January 2024
EconLit		8th July 2018 to 9th October 2023	12th October 2023	12th October 2023 to 28th December 2023	10th January 2024

Table A12 Eligibility criteria (PICOS) – economic evaluation SLR updates

Characteristics	Inclusion criteria	Exclusion criteria
Population	• Patients of any age with DMD, with any pathogenic mutation, deletion, or duplication[a]<br>• Mixed populations: if ≥80% of patients meet the eligibility criteria or outcomes are reported separately for these patients	• Mixed populations: if <80% of patients meet the eligibility criteria or outcomes are not reported separately for these patients
Intervention/ comparators	• Any treatment (actual or hypothetical) for DMD[b]	• Genetic tests or other diagnostic/screening tests, surgery, exercise or devices, or aquatic therapy
Outcomes	• Model characteristics, including:<br>○ Type, perspective, time horizon, discounting, cycle lengths, key assumptions, and sources of model inputs<br>• Costs<br>• Costs per outcome<br>• Quality adjusted life years	• Outcomes not listed

Characteristics	Inclusion criteria	Exclusion criteria
	• Life years gained<br>• Incremental cost-effectiveness ratio	
Study design	• Cost-effectiveness analysis<br>• Cost-utility analysis<br>• Cost-minimisation analysis (cost-comparison analysis)<br>• Cost-consequence analysis<br>• Cost-benefit analysis<br>• Cost offset analysis<br>• Budget impact analysis	• Reviews/editorials/commentaries/letters<br>• Case reports<br>• SLRs/NMAs[c]<br>• *In vitro*/animal studies<br>• Cost analyses/cost of illness studies
Date limits	• **2023 SLR update:** 8th July 2018 – present<br>• **2024 SLR update:** 12th October 2023 – present	• **2023 SLR update:** prior to 8th July 2018<br>• **2024 SLR update:** prior to 12th October 2023
Countries	No restriction	–
Languages	English language publications	Non-English language publications

[a]Note that the de novo SLR included studies of steroid sparing in patients with any disease, however these studies were not considered relevant for the SLR updates, as a sufficient number of studies in DMD patients were identified to focus on this population alone. Consequently, steroid sparing studies were excluded during full-text screening.
[b]The search strings included generic terms for classes of interventions in addition to terms for specific interventions. In the SLR updates, the following new interventions, that have emerged since the de novo SLR was performed, have been added to the search strings: delandistrogene moxeparvovec, fordadistrogene movaparvovec, and drisapersen.
[c]Relevant SLRs/NMAs were included at title/abstract screening stage so their bibliographic reference lists could be hand-searched for relevant studies.
DMD, Duchenne muscular dystrophy; NMA, network meta-analysis; SLR, systematic literature review.

3.1.2. Results

Across the three iterations of the economic evaluations SLR, a total of 13 publications reporting on 12 unique studies were included: four full manuscripts, five conference abstracts with three conference posters identified, and four HTA submissions. The flow of publications through the SLR is depicted in the PRISMA flow diagram in Figure A1.

Fig. A1 PRISMA flow diagram – economic evaluation SLR

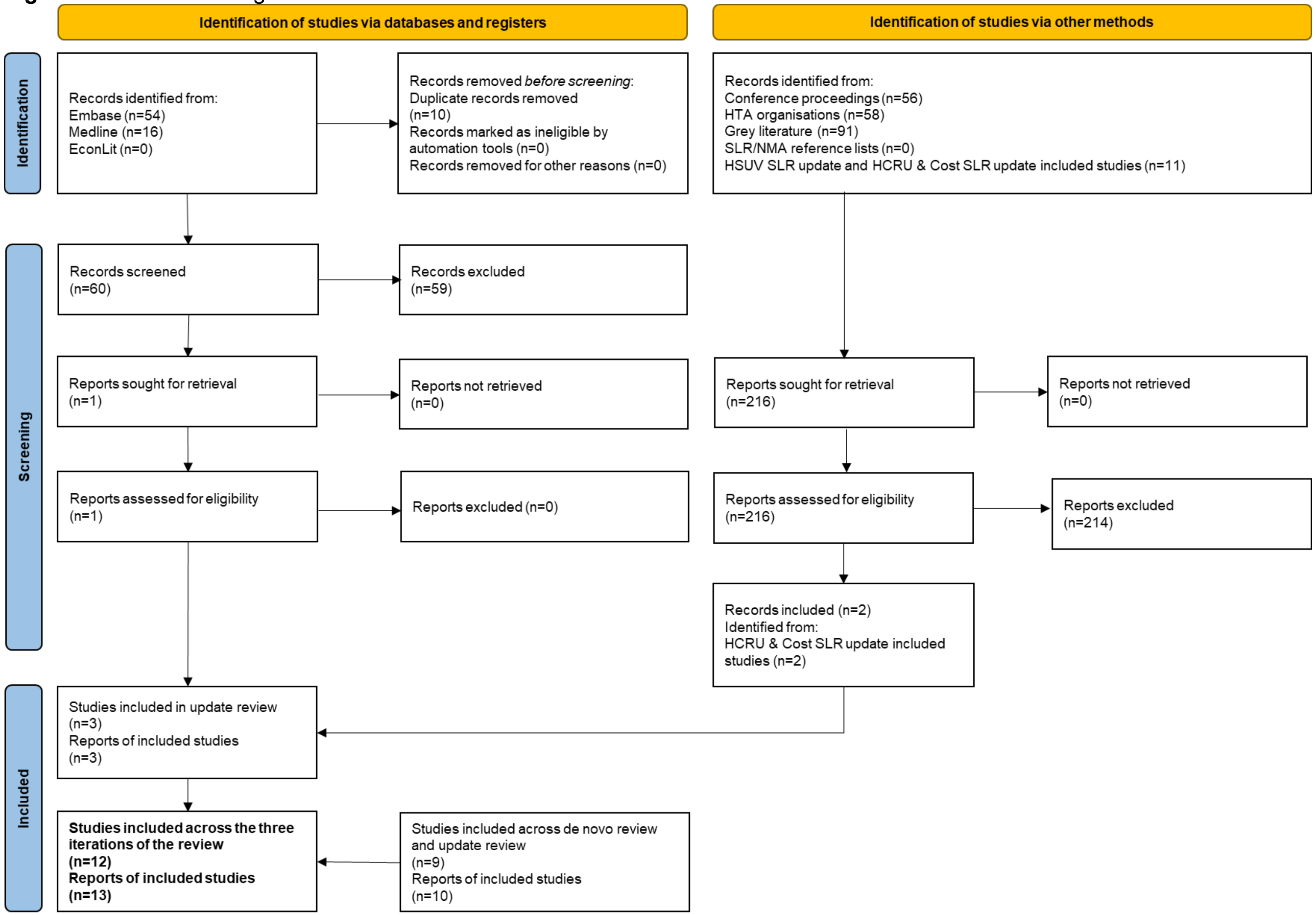

HCRU healthcare resource use; *HSUV* health state utility value, *HTA* health technology assessment, *NMA* network meta-analysis, *PRISMA* Preferred Reporting Items for Systematic Reviews and Meta-Analyses, *SLR* systematic literature review.

3.1.3. Overview of included studies

A summary of the included studies is reported in **Error! Reference source not found.**.

Table A13 Summary of the included economic evaluations (n=13)

Publication intervention/ comparator	Summary of model	Patient population	QALYs (intervention, comparator)	Costs (currency) (intervention, comparator)	ICER (per QALY gained)
**De novo SLR (n=2)**					
Landfeldt, 2017 [51]<br>Hypothetical intervention vs SoC	Cost-effectiveness analysis. Markov model with 25 health states (DMDSAT score 23 to 0 and dead). 95-year time horizon. UK NHS perspective. 1-year cycle length.	Patients with DMD over the age of 5 years old	Total QALYs: hypothetical intervention: 8.13; SoC: 7.07	Total costs (GBP 2015): hypothetical intervention: £1,737,960; SoC: £217,510	£1,442,710
	Cost-effectiveness analysis. Markov model with 5 health states (early ambulatory, late ambulatory, early non-ambulatory, late non-ambulatory, death). 95-year time horizon. UK NHS perspective. 1-year cycle length.		Total QALYs: hypothetical intervention: 7.96; SoC: 7.17	Total costs (GBP 2015): hypothetical intervention: £1,768,370; SoC: £244,120	£1,939,590
	Cost-effectiveness analysis. Markov model with 4 health states (no ventilation support, night-time ventilation support, night and day ventilation support, death). 95-year time horizon. UK NHS perspective. 1-year cycle length.		Total QALYs: hypothetical intervention: 6.39; SoC: 5.96	Total costs (GBP 2015): hypothetical intervention: £1,809,160; SoC: £284,640	£3,574,770
NICE submission, 2016 (HST3) [58]<br>Ataluren vs Soc	Cost-consequence. Markov model with 6 health states (ambulatory, non-ambulatory, non-ambulatory [VA], non-ambulatory [scoliosis], non-ambulatory [VA + scoliosis], death). Lifetime time horizon. NHS	Patients with DMD aged 5 years or older who could walk	Total QALYs: ataluren: 6.86; SoC: 3.80	Total costs (GBP 2014): ataluren: £5,744,175; SoC: £199,194	£1,818,026
			Incremental QALYs: stepped decline method: 8.19; linear	Incremental costs: stepped decline method: £5,532,819;	£675,557 and £714,908 (using the

Publication intervention/ comparator	Summary of model	Patient population	QALYs (intervention, comparator)	Costs (currency) (intervention, comparator)	ICER (per QALY gained)
	and personal social services perspective. 3-month cycle length.		extrapolation method: 11.75	linear extrapolation method: £8,400,164	stepped decline and linear extrapolation methods respectively)
			Incremental QALYs: 1.913 to 8.562	-	-
**October 2023 SLR update (n=8)**					
Carlton, 2018 [75] Deflazacort	Budget impact model over a 3-year time horizon. US commercial health plans perspective.	Patients with DMD aged 5 to 24 years old	-	Estimated incremental PMPM pharmacy cost due to deflazacort (USD): $0.008/$0.012/$0.0116 for years 1/2/3.	-
Klimchak, 2023a [50] Delandistrogene moxeparvovec + SoC vs Soc alone	Cost-utility analysis. Patient simulation model with 5 health states (early ambulatory, late ambulatory, early non-ambulatory, late non-ambulatory, death). Lifetime time horizon. US healthcare system perspective.	4-year-olds with DMD	Total QALYs (undiscounted): delandistrogene moxeparvovec + SoC: 30.55; SoC: 13.39.	Total costs (undiscounted) (USD 2021): Delandistrogene moxeparvovec + SoC: $1,164,783; SoC: $1,105,932	Incremental costs: $58,851<br>Incremental QALYs gained: 7.31
Klimchak, 2023b [50] Hypothetical intervention	Cost-utility analysis. Partitioned survival model with 3 health states (early non-ambulatory, late non-ambulatory, death).	13-year-old patient with early non-ambulatory stage DMD	Incremental QALYs gained:<br>10-year pause in disease progression: 1.32<br>20-year pause in disease progression: 2.30	Incremental costs (USD):<br>10-year pause in disease progression: $193,300<br>20-year pause in disease progression: $337,100<br>40-year pause in disease progression: $523,600	-

Publication intervention/ comparator	Summary of model	Patient population	QALYs (intervention, comparator)	Costs (currency) (intervention, comparator)	ICER (per QALY gained)
			40-year pause in disease progression: 3.57		
Lin, 2019 (updated 2022) [55]<br>Deflazcort + BSC vs prednisone + BSC	Cost-utility analysis. Partitioned survival model with 5 health states (early ambulatory, late ambulatory, early non-ambulatory, late non-ambulatory, death). Lifetime time horizon. Healthcare and societal sector perspective. Annual cycle length.	All patients with DMD over the age of 5 years old	Deflazcort + supportive care:<br>Health sector perspective: 8.40<br>Modified societal perspective: 8.40<br>Prednisone + supportive care:<br>Health sector perspective: 6.88<br>Modified societal perspective: 6.88	Total costs (USD 2017):<br>Deflazcort + supportive care:<br>Health sector perspective: $1,010,000<br>Modified societal perspective: $1,830,000<br>Prednisone + supportive care:<br>Health sector perspective: $464,000<br>Modified societal perspective: $1,240,000	Health sector perspective: $663,000<br>Modified societal perspective: $692,000
NCPE submission, 2016 [76]<br>Ataluren vs BSC	Cost-utility analysis. Markov model with 6 health states (ambulatory, non-ambulatory, non-ambulatory [scoliosis], non-ambulatory [requires assisted ventilation], non-ambulatory [requires assisted ventilation and scoliosis], death). Lifetime time horizon.	All patients with DMD	Incremental QALYs gained:<br>Base case: 5.284<br>Using NCPE preferred assumptions: 2.928	Incremental costs (EUR):<br>Base case: €5,303,434<br>Using NCPE preferred assumptions: €6,820,784	Base case: €1,003,637<br>Using NCPE preferred assumptions: €2,329,281
NICE submission, 2022 (HST22) [59]<br>Alaturen vs BSC	Cost-utility analysis. Partitioned survival model with 5 health states (ambulatory, non-ambulatory [predicted FVC >50%], non-ambulatory [predicted FVC <50%], non-ambulatory [predicted FVC <30%], death). 70-year lifetime time horizon. NHS and personal social	Ambulatory patients with DMD aged 2 years and older	Total QALYs (undiscounted): ataluren: 73.87; BSC: 50.74	Total costs: redacted	List price: £336,555<br>Re-weighted ICER for HST guidance: £145,514

Publication intervention/ comparator	Summary of model	Patient population	QALYs (intervention, comparator)	Costs (currency) (intervention, comparator)	ICER (per QALY gained)
	services perspective. 3-month cycle length.				
Quach, 2019 [59]<br>Deflazacort vs prednisone	Cost-utility analysis. Partitioned survival model. Lifetime time horizon. United States health sector and a modified societal perspective.	All patients with DMD	-	-	Health sector perspective (USD): $790,000<br>Modified societal perspective (USD): $829,000
SMC submission, 2023 (SMC2327) [64]<br>Alaturen vs BSC	Cost-utility analysis. Partitioned survival model with 6 health states (ambulatory, loss of ambulation [fully wheelchair bound], predicted FVC >50%, predicted FVC <50%, FVC < 1 litre, death). Lifetime time horizon. NHS Scotland and social care perspective. 3-month cycle length.	All patients with DMD ages 2 years and older	Total QALYs: ataluren: 47.17; BSC: 38.55	Total costs (GBP): ataluren: £5,200,244; BSC: £693,797	£522,664
**January 2024 SLR update (n=3)**					
Broomfield, 2023 [71]<br>Hypothetical intervention vs SoC	Cost-effectiveness analysis. Natural history model with 5 health states (early ambulatory, late ambulatory, early non-ambulatory, late non-ambulatory, death). Lifetime horizon.	All patients with DMD	Incremental QALYs: 0.772	Incremental costs (GBP): £1,517,000	£1,964,000
Innis, 2023 [72]<br>Delandistrogene moxeparvovec vs SoC	Cost-effectiveness analysis. Partitioned survival model with 5 health states (early ambulatory, late ambulatory, early non-ambulatory, late non-ambulatory, death). Lifetime time horizon. Annual cycle length.	All patients with DMD	-	-	-

Publication intervention/ comparator	Summary of model	Patient population	QALYs (intervention, comparator)	Costs (currency) (intervention, comparator)	ICER (per QALY gained)
Shehata, 2023 [36]<br>Ataluren vs SoC<br>Eteplirsen vs SoC	Cost-utility analysis. Partitioned survival model with 5 health states (early ambulatory, late ambulatory, early non-ambulatory, late non-ambulatory, death). 55-year time horizon. Societal perspective. 3-month cycle length.	All patients with DMD	Ataluren vs SoC model<br>Undiscounted QALYS:<br>Ataluren: 8.94, SoC: 7.59<br>Discounted QALYs:<br>Ataluren: 6.83, SoC: 5.96	Ataluren vs SoC model<br>Undiscounted costs (EGP 2021):<br>Ataluren: 58,806,706, SoC: 1,608,307<br>Discounted costs (EGP 2021):<br>Ataluren: 46,210,767, SoC: 1,115,550.06	Ataluren vs SoC model (EGP):<br>51,745,605
			Eteplirsen vs SoC model<br>Undiscounted QALYS:<br>Ataluren: 8.74, SoC: 7.59<br>Discounted QALYs:<br>Ataluren: 6.75, SoC: 5.96	Eteplirsen vs SoC model<br>Undiscounted costs (EGP 2021):<br>Eteplirsen: 70,079,768, SoC: 1,608,307<br>Discounted costs (EGP 2021):<br>Eteplirsen: 55,966,962, SoC: 1,098,856	Eteplirsen vs SoC model (EGP):<br>69,652,533

BSC best supportive care, *DMD* Duchenne muscular dystrophy, *DMDSAT* Duchenne muscular dystrophy Functional Ability Self-Assessment Tool, *EGP* Egyptian Pounds, *EUR* Euro, *FVC* forced vital capacity, *NICE* National Institute for Health and Care Excellence, *GBP* Great British Pound, *HST* highly specialised technology appraisal, *ICER* incremental cost-effectiveness ratio, *NHS* National Health Service, *PMPM* per-member-per-month, *QALY* quality adjusted life year, *SoC* standard of care, *UK* United Kingdom, *US* United States, *USD* United States Dollar, *VA* alveolar ventilation.

4. References

1. Broomfield J, Hill M, Chandler F, et al. Developing a Natural History Model for Duchenne Muscular Dystrophy. *Pharmacoecon Open*. Jan 2024;8(1):79-89. doi:10.1007/s41669-023-00450-x
2. Arber M, Garcia S, Veale T, Edwards M, Shaw A, Glanville JM. Performance of Ovid Medline search filters to identify health state utility studies. *Int J Technol Assess Health Care*. Jan 2017;33(4):472-480. doi:10.1017/S0266462317000897
3. McMaster University Health Information Research Unit. Search Filters for MEDLINE in Ovid Syntax and the PubMed translation. Accessed October 10, 2025, https://hiru.mcmaster.ca/hiru/HIRU_Hedges_MEDLINE_Strategies.aspx.
4. Domaradzki J, Walkowiak D. Quality of life and caregiving burden associated with parenting a person with Duchenne/Becker muscular dystrophy in Poland. *Orphanet J Rare Dis*. Nov 30 2024;19(1):450. doi:10.1186/s13023-024-03481-7
5. Xu RH, Dai Y, Ng SSM, Tsang HWH, Zhang S, Dong D. Assessing validity of the EQ-5D-5L proxy in children and adolescents with Duchenne muscular dystrophy or spinal muscular atrophy. *Eur J Health Econ*. Feb 2024;25(1):103-115. doi:10.1007/s10198-023-01574-x
6. Xu RH, Jiang R, Yang C, Dong D. A Qualitative Examination of the Content Validity of the EQ-5D-5L and EQ-5D-Y-3L in Adult and Paediatric Patients With Duchenne Muscular Dystrophy. *Health Expect*. Oct 2025;28(5):e70431. doi:10.1111/hex.70431
7. Do LA, Sedita LE, Klimchak AC, Salazar R, Kim DD. Cataloging health state utility estimates for Duchenne muscular dystrophy and related conditions. *Health Qual Life Outcomes*. Sep 2 2024;22(1):72. doi:10.1186/s12955-024-02287-2
8. Szabo SM, Griffin E, Iannaccone ST, Gooch KL, Audhya IF. The EQ-5D and Health Utilities Index for assessing health-related quality-of-life impact in Duchenne muscular dystrophy: Evaluating the relevance and interpretation of descriptive systems from patient and caregiver perspectives. *Advances in Patient-Reported Outcomes*. 2025/09/01/ 2025;1(3):100209. doi:https://doi.org/10.1016/j.apro.2025.100209
9. Posner N, Manjelievskaia J, Talaga AK, et al. Real-world treatment and health care utilization among patients with Duchenne muscular dystrophy by race and ethnicity in a Medicaid population. *J Manag Care Spec Pharm*. Feb 1 2025;31(2):205-213. doi:10.18553/jmcp.2025.31.2.205
10. Rudolfsen JH, Vissing J, Werlauff U, et al. Burden of Disease of Duchenne Muscular Dystrophy in Denmark - A National Register-Based Study of Individuals with Duchenne Muscular Dystrophy and their Closest Relatives. *J Neuromuscul Dis*. 2024;11(2):443-457. doi:10.3233/jnd-230133
11. Morgan CL, Godfrey J, Chandler F, Reuben E, Currie CJ. Epidemiology and healthcare resource utilisation associated with Duchenne muscular dystrophy. *Journal of Rare Diseases*. 2024/08/12 2024;3(1):23. doi:10.1007/s44162-024-00044-z
12. Innis B, Jarvis J, Renteria T, Audhya I. Household costs in the United States for accommodating functional impairments associated with Duchenne muscular dystrophy: results from a caregiver survey. *Orphanet J Rare Dis*. Jun 12 2025;20(1):301. doi:10.1186/s13023-025-03794-1
13. Diesing J, Kirschner J, Pechmann A, et al. Epidemiology, disease burden and costs of Duchenne muscular dystrophy in Germany: an observational, retrospective health claims data analysis. *Orphanet J Rare Dis*. Aug 13 2025;20(1):429. doi:10.1186/s13023-025-03906-x
14. Ghawaa Y, Lin AC, Guo JJ. The Utilization, Reimbursement, and Cost of Targeted Therapies for Duchenne Muscular Dystrophy (DMD) in US Medicaid Programs: A Descriptive Trend Analysis from 2017 to 2022. *Pharmaceut Med*. Nov 2025;39(6):445-452. doi:10.1007/s40290-025-00587-6
15. Broomfield J, Abrams KR, Freeman S, Latimer N, Rutherford MJ, Crowther MJ. Modeling the multi-state natural history of rare diseases with heterogeneous individual patient data: A simulation study. *Stat Med*. Jan 15 2024;43(1):184-200. doi:10.1002/sim.9949
16. Cavazza M, Kodra Y, Armeni P, et al. Social/economic costs and health-related quality of life in patients with Duchenne muscular dystrophy in Europe. *Eur J Health Econ*. 2016;17(Suppl 1):19-29. doi:10.1007/s10198-016-0782-5
17. Landfeldt E, Lindgren P, Bell CF, et al. The burden of Duchenne muscular dystrophy. *Neurology*. 2014;83(6):529-536. doi:doi:10.1212/WNL.0000000000000669
18. Landfeldt E, Mayhew A, Eagle M, et al. Development and psychometric analysis of the Duchenne muscular dystrophy Function Abiliity Self-Assessment Tool (DMDSAT). *Neuromuscul Disord*. 2015;25(12):937-944. doi:10.1016/j.nmd.2015.09.012

19. Landfeldt E, Lindgren P, Bell CF, et al. Health-related quality of life in patients with Duchenne muscular dystrophy: A multinational, cross-sectional study. *Dev Med Child Neurol*. 2016;58(5):508-515. doi:10.1111/dmcn.12938
20. Landfeldt E, Lindgren P, Bell CF, et al. Quantifying the burden of caregiving in Duchenne muscular dystrophy. *J Neurol*. 2016;263(5):906-915. doi:10.1007/s00415-016-8080-9
21. Landfeldt E, Edström J, Buccella F, Kirschner J, Lochmüller H. Duchenne muscular dystrophy and caregiver burden: a systematic review. *Dev Med Child Neurol*. Oct 2018;60(10):987-996. doi:10.1111/dmcn.13934
22. Magnetta DA, Kang J, Wearden PD, Smith KJ, Feingold B. Cost-Effectiveness of Ventricular Assist Device Destination Therapy for Advanced Heart Failure in Duchenne Muscular Dystrophy. *Pediatr Cardiol*. 2018;39(6):1242-1248.
23. Pangalila RF, van den Bos GA, Stam HJ, van Exel NJ, Brouwer WB, Roebroeck ME. Subjective caregiver burden of parents of adults with Duchenne muscular dystrophy. *Disabil Rehabil*. 2012;34(12):988–996.
24. Andreozzi V, Labisa P, Mota M, et al. Quality of life and informal care burden associated with duchenne muscular dystrophy in Portugal: the COIDUCH study. *Health Qual Life Outcomes*. 2022;20(1):36. doi:https://dx.doi.org/10.1186/s12955-022-01941-x
25. Audhya I, Szabo SM, Bever A, O'Sullivan F, Malone D, Feeny D. Estimating Health State Utilities in Duchenne Muscular Dystrophy (DMD) Using the EQ-5D and Health Utilities Index (HUI). presented at: Annual Congress of the World Muscle Society; 2022;
26. Audhya IF, Szabo SM, Bever A, et al. Estimating health state utilities in Duchenne muscular dystrophy using the health utilities index and EQ-5D-5L. *JPRO*. 2023;7(1):132. doi:https://dx.doi.org/10.1186/s41687-023-00671-y
27. Audhya FI, Szabo S, Bever AE, et al. Time Trade-Off Utility Values for Health States Characterizing Progressive Muscular Degeneration in Duchenne Muscular Dystrophy (DMD). *Value Health*. 2022;25(Suppl 7):S574. doi:https://dx.doi.org/10.1016/j.jval.2022.04.1519
28. Audhya IF, Szabo SM, Bever A, et al. Estimating health state utilities in Duchenne muscular dystrophy using the health utilities index and EQ-5D-5L. *J Patient Rep Outcomes*. 2023;7(1):132. doi:https://dx.doi.org/10.1186/s41687-023-00671-y
29. Szabo S, Filipovic Audhya I, Griffin E, et al. PCR192 Content Validity of the EQ-5D and Health Utilities Index (HUI) to Assess Health-Related Quality-of-Life (HRQoL) Impact in Duchenne Muscular Dystrophy (DMD). *Value Health*. 2023;26(Suppl 12):S486. doi:https://dx.doi.org/10.1016/j.jval.2023.09.2629
30. Crossnohere N, Fischer R, Bridges J. Health state utility values for stages of functional decline in Duchenne muscular dystrophy: an international study. *Value Health*. 2020;23(Suppl 1):S344. doi:https://dx.doi.org/10.1016/j.jval.2020.04.1307
31. Crossnohere NL, Fischer R, Lloyd A, Prosser LA, Bridges JFP. Assessing the Appropriateness of the EQ-5D for Duchenne Muscular Dystrophy: A Patient-Centered Study. *Med Decis Making*. 2021;41(2):209-221. doi:https://dx.doi.org/10.1177/0272989X20978390
32. Gallop K, Foerster D, Lawrence C, Denjean E, Van Der Wild J, Acaster S. Estimation of Health Utilities for NON-Ambulatory Duchenne Muscular Dystrophy (DMD) Patients and Their Caregivers. *Value Health*. 2020;23(Suppl 2):S602. doi:https://dx.doi.org/10.1016/j.jval.2020.08.1192
33. Landfeldt E, Lindberg C, Sejersen T. Improvements in health status and utility associated with ataluren for the treatment of nonsense mutation Duchenne muscular dystrophy. *Muscle Nerve*. 2020;61(3):363-368. doi:https://dx.doi.org/10.1002/mus.26787
34. Landfeldt E, Zhang R, Childs AM, et al. Assessment of face validity of a disease model of nonsense mutation Duchenne muscular dystrophy: a multi-national Delphi panel study. *J Med Econ*. 2022;25(1):808-816. doi:https://dx.doi.org/10.1080/13696998.2022.2085444
35. Rowen D, Powell P, Mukuria C, Carlton J, Norman R, Brazier J. Deriving a Preference-Based Measure for People With Duchenne Muscular Dystrophy From the DMD-QoL. *Value Health*. 2021;24(10):1499-1510. doi:https://dx.doi.org/10.1016/j.jval.2021.03.007
36. Shehata ZH, Rabea H, El Sherif R, Abdelrahim ME, Dawoud DM. Estimating Societal Cost of Illness and Patients' Quality of Life of Duchenne Muscular Dystrophy in Egypt. *Value Health Reg Issues*. 2023;33:10-16. doi:https://dx.doi.org/10.1016/j.vhri.2022.08.006
37. Szabo S, Filipovic Audhya I, Bever A, et al. The Association between Patient- and Caregiver-Reported Utility Values in Duchenne Muscular Dystrophy (DMD). *Value Health*. 2023;26(Suppl 6):S7. doi:https://dx.doi.org/10.1016/j.jval.2023.03.043
38. Xu RH, Dai Y, Ng SSM, Tsang HWH, Zhang S, Dong D. Assessing validity of the EQ-5D-5L proxy in children and adolescents with Duchenne muscular dystrophy or spinal muscular atrophy. *Eur J Health Econ*. 2023;25(1):103-115. doi:https://dx.doi.org/10.1007/s10198-023-01574-x

39. Castro D, Evans J, Jones C, et al. Introduction to the Duchenne Muscular Dystrophy Burden-of-Illness Study Expanded (US AND SPAIN). *Value Health*. 2023;26(Suppl 12):S68. doi:https://dx.doi.org/10.1016/j.jval.2023.09.363
40. Bach JR, Tran J, Durante S. Cost and physician effort analysis of invasive vs. noninvasive respiratory management of Duchenne muscular dystrophy. *Am J Phys Med Rehabil*. 2015;94(6):474-482. doi:https://dx.doi.org/10.1097/PHM.0000000000000228
41. Conway KM, Grosse SD, Ouyang L, Street N, Romitti PA. Direct costs of adhering to selected Duchenne muscular dystrophy Care Considerations: Estimates from a midwestern state. *Muscle Nerve*. 2022;65(5):574-580. doi:https://dx.doi.org/10.1002/mus.27505
42. Donaldson A, Guntrum D, Ciafaloni E, Statland J. Achieving Life Milestones in Duchenne/Becker Muscular Dystrophy: A Retrospective Analysis. *Neurol Clin Pract*. 2021;11(4):311-317. doi:https://dx.doi.org/10.1212/CPJ.0000000000000970
43. Flores D, Ribate MP, Montolio M, Ramos FJ, Gomez M, Garcia CB. Quantifying the economic impact of caregiving for Duchenne muscular dystrophy (DMD) in Spain. *Eur J Health Econ*. 2020;21(7):1015-1023. doi:https://dx.doi.org/10.1007/s10198-020-01197-6
44. Hurvitz MS, Bhattacharjee R, Lesser DJ, Skalsky AJ, Orr JE. Determinants of usage and nonadherence to noninvasive ventilation in children and adults with Duchenne muscular dystrophy. *J Clin Sleep Med*. 2021;17(10):1973-1980. doi:https://dx.doi.org/10.5664/jcsm.9400
45. Iff J, Zhong Y, Gupta D, et al. Disease Progression Stages and Burden in Patients with Duchenne Muscular Dystrophy Using Administrative Claims Supplemented by Electronic Medical Records. *Adv Ther*. Jun 2022;39(6):2906-2919. doi:10.1007/s12325-022-02117-1
46. Iff J, Zhong Y, Tuttle E, Gupta D, Paul X, Henricson E. Real-world evidence of eteplirsen treatment effects in patients with Duchenne muscular dystrophy in the USA. *J Comp Eff Res*. 2023;12(9):e230086. doi:https://dx.doi.org/10.57264/cer-2023-0086
47. Iff J, Tuttle E, Gerrits C, Gupta D, Zhong Y. DMD - THERAPY: P.291 Real-world evidence of eteplirsen treatment effects on Duchenne muscular dystrophy related health outcomes using claims data in the United States. *Neuromuscul Disord*. 2020;30(Suppl 1):S131-S132. doi:https://dx.doi.org/10.1016/j.nmd.2020.08.288
48. Innis B, Henry A, Zein M, et al. EE401 Characterizing the Impact on Work Productivity in Patients with Duchenne Muscular Dystrophy and Caregivers: An Economic Analysis. *Value Health*. 2023;26(6):S133. doi:10.1016/j.jval.2023.03.702
49. Klimchak AC, Szabo SM, Qian C, Popoff E, Iannaccone S, Gooch KL. Characterizing demographics, comorbidities, and costs of care among populations with Duchenne muscular dystrophy with Medicaid and commercial coverage. *J Manag Care Spec Pharm*. 2021;27(10):1426-1437. doi:https://dx.doi.org/10.18553/jmcp.2021.27.10.1426
50. Klimchak AC, Sedita LE, Rodino-Klapac LR, et al. Assessing the value of delandistrogene moxeparvovec (SRP-9001) gene therapy in patients with Duchenne muscular dystrophy in the United States. *J Mark Access Health Policy*. 2023;11(1):2216518. doi:10.1080/20016689.2023.2216518
51. Landfeldt E, Alfredsson L, Straub V, Lochmüller H, Bushby K, Lindgren P. Economic Evaluation in Duchenne Muscular Dystrophy: Model Frameworks for Cost-Effectiveness Analysis. *PharmacoEconomics*. Feb 2017;35(2):249-258. doi:10.1007/s40273-016-0461-5
52. Landfeldt E, Lindgren P, Bell CF, et al. Compliance to Care Guidelines for Duchenne Muscular Dystrophy. *J Neuromuscul Dis*. 2015;2:63-72. doi:10.3233/JND-140053
53. Landfeldt E, Eagle M, Straub V, Lochmuller H, Bushby K, Lindgren P. Mortality Cost of Duchenne Muscular Dystrophy. *Glob Reg Health Technol Assess*. 2017;4(1):e100-e103. doi:10.5301/grhta.5000260
54. Lang SM, Alsaied T, Moore RA, Rattan M, Ryan TD, Taylor MD. Conservative gadolinium administration to patients with Duchenne muscular dystrophy: decreasing exposure, cost, and time, without change in medical management. *Int J Cardiovasc Imaging*. 2019;35(12):2213-2219. doi:https://dx.doi.org/10.1007/s10554-019-01670-1
55. Lin G, Agboola F, Otuonya I. Deflazacort, eteplirsen, and golodirsen for Duchenne muscular dystrophy: effectiveness and value-evidence report. *Institute for Clinical and Economic Review*. 2019:115-133.
56. Magliano L, D'Angelo MG, Vita G, et al. Psychological and practical difficulties among parents and healthy siblings of children with Duchenne vs. Becker muscular dystrophy: an Italian comparative study. *Acta Myol*. 2014;33(3):136-43.
57. Mujwara D, McGonigal R, Ford J, Hayes M, Ayyagari R. EE523 Assessing the Economic and Quality of Life Impact of Treatment in Duchenne Muscular Dystrophy. *Value Health*. 2022;25(12):S158. doi:10.1016/j.jval.2022.09.764

58. National Institute for Health and Care Excellence (NICE). Ataluren for treating Duchenne muscular dystrophy caused by a nonsense mutation in the dystrophin gene. Highly specialised technologies guidance (HST3) [ID 428]. Accessed October 10, 2025, https://www.nice.org.uk/guidance/hst22.
59. National Institute for Health and Care Excellence (NICE). Ataluren for treating Duchenne muscular dystrophy with a nonsense mutation in the dystrophin gene. Highly specialised technologies guidance (HST22) [ID1642]. Accessed October 10, 2025, https://www.nice.org.uk/guidance/hst22.
60. Posner N, Manjelievskaia J, Talaga AK, et al. All-Cause Healthcare Costs By Race Among Medicaid-Insured Males with Duchenne Muscular Dystrophy Using U.S. Real-World Data. *Value Health*. 2023;26(Suppl 6):S96. doi:https://dx.doi.org/10.1016/j.jval.2023.03.505
61. Reynolds EL, Gallagher G, Hill CE, et al. Costs and Utilization of New-to-Market Neurologic Medications. *Neurology*. 2023;100(9):E884-E898. doi:https://dx.doi.org/10.1212/WNL.0000000000201627
62. Rodriguez AA, Amayra I, Lopez-Paz JF, et al. The Role of Associations in Reducing the Emotional and Financial Impact on Parents Caring for Children with Duchenne Muscular Dystrophy: A Cross-Cultural Study. *Int J Environ Res Public Health*. 2022;19(19):12334. doi:https://dx.doi.org/10.3390/ijerph191912334
63. Schreiber-Katz O, Klug C, Thiele S, et al. Comparative cost of illness analysis and assessment of health care burden of Duchenne and Becker muscular dystrophies in Germany. *Orphanet J Rare Dis*. 2014;9:210. doi:https://dx.doi.org/10.1186/s13023-014-0210-9
64. Scottish Medicines Consortium (SMC). Ataluren (Translarna®) for Duchenne muscular dystrophy (SMC2327). Accessed October 10, 2025, https://www.scottishmedicines.org.uk/medicines-advice/ataluren-translarna-uo-pathway-smc2327/.
65. Soelaeman RH, Smith MG, Sahay K, et al. Labor market participation and productivity costs for female caregivers of minor male children with Duchenne and Becker muscular dystrophies. *Muscle Nerve*. 2021;64(6):717-725. doi:https://dx.doi.org/10.1002/mus.27429
66. Thayer S, Bell C, Mc CM. The direct cost of managing a rare disease: Assessing medical and pharmacy costs associated with duchenne muscular dystrophy in the United States. *J Manag Care Spec Pharm*. 2017;23(6):633-641. doi:https://dx.doi.org/10.18553/jmcp.2017.23.6.633
67. Villa C, Auerbach SR, Bansal N, et al. Current Practices in Treating Cardiomyopathy and Heart Failure in Duchenne Muscular Dystrophy (DMD): Understanding Care Practices in Order to Optimize DMD Heart Failure Through ACTION. *Pediatr Cardiol*. 2022;43(5):977-985. doi:https://dx.doi.org/10.1007/s00246-021-02807-7
68. Vry J, Gramsch K, Rodger S, et al. European Cross-Sectional Survey of Current Care Practices for Duchenne Muscular Dystrophy Reveals Regional and Age-Dependent Differences. *J Neuromuscul Dis*. 2016;3(4):517-527. doi:https://dx.doi.org/10.3233/JND-160185
69. White T, Wasson L, Hadker N, et al. Medical Chart Audit Study to Demonstrate Shortcomings of Corticosteroids in Treatment of Duchenne Muscular Dystrophy. *Value Health*. 2023;26(Suppl 6):S248. doi:https://dx.doi.org/10.1016/j.jval.2023.03.1366
70. Bonarrigo K, McGuire M, Dorich JM, et al. Use of supported standing in males with Duchenne muscular dystrophy: Individual and family perspectives. *J Pediatr Rehabil Med*. 2023;16(3):553-569. doi:https://dx.doi.org/10.3233/PRM-220026
71. Broomfield J, Abrams K, Latimer N, Guglieri M, Rutherford M, Crowther M. Natural history of Duchenne muscular dystrophy in the United Kingdom: A descriptive study using the Clinical Practice Research Datalink. *Brain Behav*. 2023;13(12):e3331. doi:https://dx.doi.org/10.1002/brb3.3331
72. Innis B, Henry A, Klimchak AC, Patel S, Gooch K, Audhya I. EE392 The Potential Impact of Delandistrogene Moxeparvovec on Work Productivity on Individuals With Duchenne Muscular Dystrophy in the United States (US). *Value Health*. 2023;26(12):S126. doi:10.1016/j.jval.2023.09.658
73. Strober J, Ishigaki K, Merla V, et al. Healthcare Resource Utilization for Pediatric and Adolescent Duchenne Muscular Dystrophy Patients: Analysis of Real-World Data. *Value Health*. 2023;26(Suppl 12):S529. doi:https://dx.doi.org/10.1016/j.jval.2023.09.2847
74. Strober J, Ishigaki K, Merla V, et al. The Impact of Duchenne Muscular Dystrophy on Caregiver Employment: A Survey in Europe, Japan, and the United States. *Value Health*. 2023;26(Suppl 12):S476. doi:https://dx.doi.org/10.1016/j.jval.2023.09.2580
75. Carlton R, Regan T, Narayanan S. The economic impact of deflazacort for the treatment of duchenne muscular dystrophy. *J Manag Care Spec Pharm*. 2018;24(4-A SUPPL.):S90-91.
76. National Centre for Pharmacoeconomics (NCPE). Cost-effectiveness of Ataluren (Translarna®) for the treatment of Duchenne muscular dystrophy resulting from a nonsense mutation in the dystrophy gene in ambulatory patients aged 5 years and older. Accessed October 10, 2025, https://www.ncpe.ie/wp-content/uploads/2014/12/NCPE-summary-of-ataluren-1.pdf.
